# Unassisted solar lignin valorisation using a compartmented photo-electro-biochemical cell

**DOI:** 10.1038/s41467-019-13022-7

**Published:** 2019-11-12

**Authors:** Myohwa Ko, Le Thanh Mai Pham, Young Jin Sa, Jinwoo Woo, Trang Vu Thien Nguyen, Jae Hyung Kim, Dongrak Oh, Pankaj Sharma, Jungki Ryu, Tae Joo Shin, Sang Hoon Joo, Yong Hwan Kim, Ji-Wook Jang

**Affiliations:** 1 School of Energy and Chemical Engineering, Ulsan National Institute of Science and Technology (UNIST), Ulsan, 44919 Republic of Korea; 2 Department of Energy Engineering, Ulsan National Institute of Science and Technology (UNIST), Ulsan, 44919 Republic of Korea; 3 UNIST Central Research Facilities & School of Natural Science, Ulsan National Institute of Science and Technology (UNIST), 50 UNIST-gil, Ulsan, 44919 Republic of Korea; 4 Present Address: Department of Chemistry, Kwangwoon University, 20 Gwangwoon-ro, Nowon-gu, Seoul, 01897 Republic of Korea

**Keywords:** Biocatalysis, Electrocatalysis, Solar fuels

## Abstract

Lignin is a major component of lignocellulosic biomass. Although it is highly recalcitrant to break down, it is a very abundant natural source of valuable aromatic carbons. Thus, the effective valorisation of lignin is crucial for realising a sustainable biorefinery chain. Here, we report a compartmented photo-electro-biochemical system for unassisted, selective, and stable lignin valorisation, in which a TiO_2_ photocatalyst, an atomically dispersed Co-based electrocatalyst, and a biocatalyst (lignin peroxidase isozyme H8, horseradish peroxidase) are integrated, such that each system is separated using Nafion and cellulose membranes. This cell design enables lignin valorisation upon irradiation with sunlight without the need for any additional bias or sacrificial agent and allows the protection of the biocatalyst from enzyme-damaging elements, such as reactive radicals, gas bubbles, and light. The photo-electro-biochemical system is able to catalyse lignin depolymerisation with a 98.7% selectivity and polymerisation with a 73.3% yield using coniferyl alcohol, a lignin monomer.

## Abstract (web summary)

The effective valorisation of lignin is crucial for realizing a sustainable biorefinery. Here, the authors report that a compartmented photo-electro-biochemical design enables unassisted, selective, and stable solar lignin valorisation without the need for any additional bias or chemicals.

## Introduction

Biomass is considered a promising replacement for fossil fuels because it is the most abundant carbon source in nature and is carbon neutral^1,2^. There has been remarkable progress on biomass conversion technologies, especially the conversion of sugar or starch crops to biofuels and various chemicals. However, the use of edible biomass as a feedstock is controversial in terms of ethics and cost^3^. Hence, waste biomass, so-called lignocellulosic biomass, such as wood residues, straw, and crop stover, have recently emerged as promising carbon sources^3^.

Lignin is one of the three major components of lignocellulosic biomass, together with cellulose and hemicellulose. It is nature’s most abundant source of aromatic carbon compounds and can be potentially transformed into high-value products^4–11^. However, because of its complex/irregular chemical structure and currently limited processing technology, more than 99% of lignin is abandoned or burned^12^. To make lignocellulosic biomass a more compatible renewable carbon source, an effective method for lignin valorisation is vital. The key issue here is finding effective strategies for the selective cleavage of carbon–oxygen bonds (C–O–C), more specifically, the β-ether (β-O-4) bond connecting the three basic aromatic units (coumaryl alcohol, coniferyl alcohol, and sinapyl alcohol); this is a critical step for the depolymerisation of lignin to yield valuable aromatic chemicals and feedstocks^13–17^.

Various treatment technologies including physical (ball milling, ultrasonication, plasma irradiation, and microwave heating), chemical (using an organic or inorganic acid), or catalytic (with a heterogeneous catalyst) methods have been used for lignin valorisation^18–21^. However, these processes are usually energy-intensive, requiring high temperatures and pressures, and environmentally unfriendly, producing chemical waste. Most importantly, selective carbon–oxygen bond breakage is not plausible using these conventional methods. However, microorganisms including fungi and bacteria have been selectively degrading lignin for more than 300 million years, although the detailed mechanism remains elusive^22^. In particular, the lignin peroxidase isozyme H8 (LiPH8) biocatalyst from white rot fungi has received much attention because of its exceptional ability for the selective cleavage of β-O-4 bonds in lignin^23–25^. However, a critical limitation of biocatalyst systems is that hydrogen peroxide (H_2_O_2_), which acts as an electron acceptor, must be provided from an external source, and its high concentration is detrimental to enzyme stability, limiting the scale-up of these systems.

The integration of the H_2_O_2_ generation system with the biocatalytic conversion system allows for continuous production and utilisation of H_2_O_2_, which would allow the use of low concentrations of H_2_O_2_ and the production of large-scale devices. The current method of producing H_2_O_2_ is predominantly based on the anthraquinone process, which consists of multi-step processes and requires additional separation steps in the presence of high pressure H_2_ and expensive precious metal catalysts^26^. Direct reaction of H_2_ and O_2_ on a catalyst is a simple and clean process, but this requires high-pressure gases (H_2_ and O_2_)^27,28^. Therefore, the anthraquinone process and direct H_2_O_2_ synthesis are not compatible with biocatalytic systems. In contrast, electrochemical H_2_O_2_ production via two-electron O_2_ reduction is a simple, clean, safe, and low-cost process^29–31^. Furthermore, this process operates under ambient pressure and temperatures, enabling its facile integration with the biocatalytic system. However, a critical limitation remains: additional electrical energy must be supplied to operate this integrated system.

On the other hand, photocatalysis can also produce H_2_O_2_ in a straightforward, clean manner and can be operated under ambient conditions like electrocatalytic H_2_O_2_ production. On the irradiation of the photocatalyst with light, excited electrons are produced in the conduction band, and these are then utilised for O_2_ reduction, producing H_2_O_2_. Recently, several groups have combined this photocatalytic H_2_O_2_ system with biocatalysts and applied them for various chemical reactions. For example, a modified powder-type TiO_2_ photocatalyst was utilised for H_2_O_2_ generation, and it was integrated with a biocatalytic system of peroxygenases for the selective oxyfunctionalisations of carbon–hydrogen bonds, which has been one of the major challenges in organic synthesis^32–35^. However, this integrated system (biocatalytic system with photocatalytically generated H_2_O_2_) has been rarely applied for lignin valorisation, and most of the research has been focused on developing an efficient powder-type photocatalyst, such as Nb_2_O_5_/TiO_2_ heterojunction, metal-doped TiO_2_ or graphene–TiO_2_ nanocomposite for direct lignin conversion rather than for H_2_O_2_ production^12,36^. One of the important limitations of the integrated system is that, for efficient H_2_O_2_ generation using powder-type photocatalysts, additional chemicals are required to scavenge the remaining hole in the valence band. Most crucially, the reactive oxygen species (ROS) generated during the photochemical reaction not only destabilise the biocatalyst but also cause the random cracking/breaking of the lignin structure, thus decreasing the selectivity of lignin conversion dramatically.

**Fig. 1:**
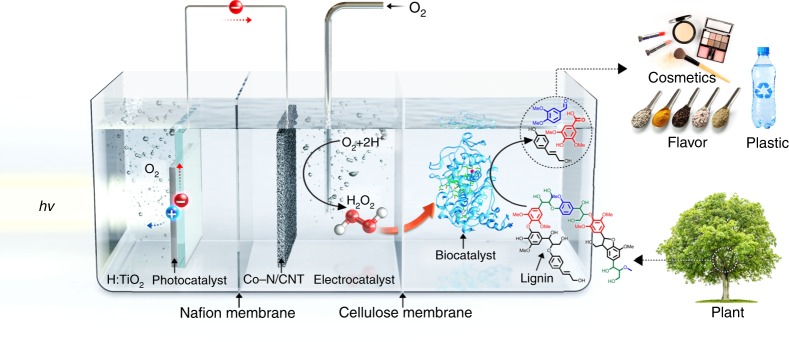
Schematic illustration of the three-compartment photo-electro-biochemical reactor. The photo-electro-biochemical reactor consists of three separate compartments. The first compartment (left-hand side) is the anodic part of the photo-electrochemical cell where water oxidation takes place. The cell comprises a cubic reactor with a quartz window on the left-hand side for the photochemical reactions on the TiO_2_ photoanode surface. Rutile TiO_2_ nanowire films grown on fluorine-doped tin oxide (FTO) glass substrate are used as the photoanode. Hydrogen treatment was performed to enhance the photocatalytic activity of TiO_2_ photoanode (H:TiO_2_). The photoanode is connected to the Co–N/carbon nanotube (CNT) cathode through a copper wire for in situ H_2_O_2_ generation in the second compartment (middle part). A Nafion proton exchange membrane separates the anodic and cathodic parts of the photo-electrochemical cell. The use of a Nafion membrane also helps to prevent the oxidation of photogenerated H_2_O_2_ on the highly photoactive H:TiO_2_ photoanode surface. To carry out the continuous O_2_ reduction to H_2_O_2_ on the Co–N/CNT cathode surface, the second compartment is also equipped with continuous O_2_ gas supply. However, the presence of •O_2_^−^ radicals, a high H_2_O_2_ concentration, and continuous O_2_ bubbling have adverse effects on the biocatalytic activity of enzymes. Therefore, the photo-electrochemical and biochemical cells are separated by a cellulose membrane. The schematic of the second and third compartments shows the gradual increase in the concentration of photogenerated H_2_O_2_ and continuous flow to the biocatalytic system (third compartment), which are ideal conditions for selective lignin conversion

Here, we propose a compartmented photo-electro-biochemical cell, in which three catalytic systems (a photocatalyst for photovoltage generation, an electrocatalyst for H_2_O_2_ production, and a biocatalyst for lignin valorisation) are integrated for selective lignin valorisation without the need for electrical energy or additional chemicals (Fig. 1). Importantly, the compartmentalisation of the three catalytic systems using Nafion and cellulose membranes stabilises the biocatalyst from enzyme-damaging elements, such as ROS, the high concentration of H_2_O_2_, shear stress from gas (O_2_) bubbles, and light. Moreover, because the lignin and photocatalyst are separated from each other, the dark-coloured native lignin does not reduce the light adsorption efficiency of the photocatalyst, a problem that has plagued the use of a photocatalyst for lignin valorisation (Fig. 1). The compartmented photo-electro-biochemical cell can catalyse the depolymerisation of lignin dimer with high selectivity (>95%) and the polymerisation of a benchmark lignin monomer, coniferyl alcohol, in 73.3% yield. The catalytic performance of the three-compartment system is superior to those of single-compartment and two-compartment catalytic systems.

## Results

### Design of the photo-electro-biochemical system

The designed photo-electro-biochemical system is composed of three compartments (photocatalyst anode, electrocatalyst cathode, and biocatalyst part), as shown in Fig. 1. Each cell is filled with an appropriate solution, and the cells are separated by Nafion and cellulose membranes. In the first compartment, a semiconductor photocatalyst receives the solar energy, and photoexcitation generates charge carriers. The photogenerated hole in the valence band oxidises water to O_2_, and the electrons move to the second compartment, where the electrocatalyst selectively reduces O_2_ to H_2_O_2_. The produced H_2_O_2_ is transferred through the size-selective cellulose membrane to the third compartment, where lignin valorisation via the biocatalyst occurs with the aid of the permeated H_2_O_2_. Importantly, the two membranes between each catalytic system prevent the permeation of O_2_ bubbles and ROS, such as hydroxyl radicals (•OH) and superoxide anions (•O^2−^), thus protecting the biocatalyst. In addition, the compartmented design ensures that the biocatalyst is not exposed to a high concentration of H_2_O_2_ and intense light because of the gradual permeation of H_2_O_2_ through the cellulose membrane and the protection of incident light by a back cover of the photoelectrode (Supplementary Fig. 1a, b), respectively. These features of the photo-electro-biochemical process allow for a continuous and stable lignin valorisation (Fig. 1).

### Unassisted photo-electrochemical H_2_O_2_ production

**Fig. 2:**
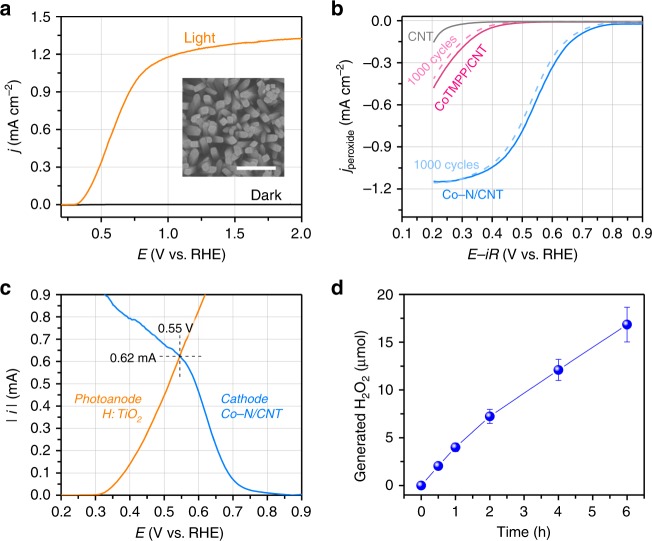
Photo-electrocatalytic production of H_2_O_2_ in the three-compartment reactor. **a** Photocurrent density of the H:TiO_2_ electrode under illumination (orange line) and in the dark (black line) in 0.1 M phosphate borate solution at pH 4.5. Inset: SEM image of H:TiO_2_. Sacle bar is 1 µm. **b** Linear sweep voltammetry (LSV) curves of Co–N/CNT (sky blue), CoTMPP/CNT (pink), and CNT (grey) catalysts before (solid line) and after (dashed line) potential cycling tests. The current densities for H_2_O_2_ production are shown and were obtained via rotating ring disk electrode measurements at an electrode rotation speed of 1,600 rpm in O_2_-saturated 0.1 M phosphate borate solution at pH 4.5. **c** LSV curves of H:TiO_2_ photoanode (orange line) under simulated 1 sun (AM1.5 G) illumination in 0.1 M phosphate borate solution at pH 4.5 and LSV curves of Co–N/CNT cathode (sky blue line) in O_2_-saturated 0.1 M phosphate borate solution at pH 4.5. **d** Amount of H_2_O_2_ generated over time in 0.1 M phosphate borate solution at pH 4.5 during the operation of the integrated three-compartment reactor. Error bars indicate the standard deviation

As a photoanode for water oxidation, a rutile TiO_2_ nanowire film was used. This film was hydrothermally grown on a fluorine-doped tin oxide (FTO) glass substrate^37^ and was subsequently annealed in a hydrogen atmosphere to improve the charge transfer properties of TiO_2_ (denoted H:TiO_2_)^38^. The X-ray diffraction (XRD) pattern of H:TiO_2_ (Supplementary Fig. 2a) shows two diffraction peaks at 36.1° and 62.8°, which is consistent with that of rutile TiO_2_. There was no significant change in the UV–vis spectra of the TiO_2_ films upon hydrogen treatment (Supplementary Fig. 2b). The scanning electron microscopy (SEM) image of H:TiO_2_ (Supplementary Fig. 3) reveals that a homogeneous film was formed on the FTO substrate, consisting of vertically aligned nanowire arrays of 100–200 nm in diameter. Phosphate borate solution at pH 4.5 was utilised as an electrolyte. Under sunlight illumination, the photocurrent density of the bare TiO_2_ nanowire photoanode at 1.23 V (vs. reversible hydrogen electrode, RHE) was 0.97 mA cm^−2^, which is far higher than that of planar-type TiO_2_ electrode (0.11 mA cm^−2^) because of the improved charge transfer rate and increased surface area due to the nanowire morphology (Supplementary Fig. 4a). Upon hydrogen treatment of TiO_2_ (H:TiO_2_), the photocurrent density was further increased to 1.25 mA cm^−2^ with almost the same onset potential of 0.40 V (vs. RHE) and there was no sign of a decrease in its performance for 12 h (Fig. 2a and Supplementary Fig. 4).

The photovoltage generated at the anode is used to catalyse O_2_ reduction at the cathode, completing the unassisted production of H_2_O_2_. As H_2_O_2_ production electrocatalysts, cobalt porphyrins have shown high activity and selectivity for the two-electron oxygen reduction reaction (ORR)^39,40^. However, these molecular catalysts are incompatible with photocatalysis cells because of their instability^41^. In order to endow catalytic stability while preserving the intrinsic activity of the Co-porphyrin-based molecular catalysts, we used a silica-protective-layer-assisted synthesis^42,43^ (see the “Methods” section for detail). In the resulting catalyst, atomically dispersed Co–N_*x*_ sites are homogeneously generated on carbon nanotubes (denoted Co–N/CNT) without the aggregation of the Co species (Supplementary Fig. 5a, b). For comparison, a cobalt porphyrin molecular catalyst immobilised on the CNTs was also prepared (CoTMPP/CNT). The Co–N/CNT catalyst was found to contain 0.8 and 1.3 wt% of Co and N, respectively, as determined by inductively coupled plasma optical emission spectrometry and combustion elemental analysis, respectively. Co 2*p* and N 1*s* X-ray photoelectron spectroscopy (XPS) scans (Supplementary Fig. 6) suggest the presence of oxidised Co species (satellite peaks) and four types of N species on the surface of the Co–N/CNT catalyst. Extended X-ray absorption fine structure analysis (Supplementary Fig. 7) reveals that the Co–N coordination bonds (Co–N_*x*_ sites) of the cobalt porphyrin precursor were retained in the Co–N/CNT catalyst after the high-temperature treatment, and there is no observable Co–Co bond peak, indicating that the aggregation of Co metal was minimised by the silica-protective-layer strategy.

The ORR activity and selectivity of the Co–N/CNT and CoTMPP/CNT catalysts were investigated using the rotating ring disk electrode (RRDE) technique (Fig. 2b and Supplementary Fig. 8). The ORR polarisation curve shows that the Co–N/CNT catalyst started to generate the H_2_O_2_ production current from 0.78 V (vs. RHE) in phosphate borate solution at pH 4.5 (Fig. 2b). This onset potential is very close to thermodynamic equilibrium potential for two-electron ORR with an overpotential of only 0.04 V (Supplementary Note 1), indicating the excellent catalytic activity of the Co–N/CNT catalyst. In contrast, CoTMPP/CNT and pristine CNT required much larger overpotentials of 0.33 and 0.47 V (Fig. 2b), respectively, suggesting that the heat-treated Co–N_*x*_ structure results in high activity. RRDE and Koutecky–Levich analyses show the consistently high H_2_O_2_ selectivity (48–62%) of the Co–N/CNT catalyst (Supplementary Fig. 8b). The molecular CoTMPP/CNT catalyst shows a better H_2_O_2_ selectivity of 60–64% than Co–N/CNT, which is attributed to its well-defined Co–N_4_ structure. However, after being subjected to prolonged potential cycling tests, CoTMPP/CNT showed a decline in the activity for H_2_O_2_ production of around 20%, whereas the Co–N/CNT catalyst mostly maintained its activity (Fig. 2b). Scanning transmission electron microscopy (STEM) images of Co–N/CNT before and after the stability test were nearly the same, indicating that atomically dispersed Co species are not agglomerated or detached from CNT support (Supplementary Fig. 5). The Co–N/CNT catalyst is also active for electrochemical H_2_O_2_ production at low pH (in 0.1 M HClO_4_), extending the potential applicability of this catalyst to photocatalytic H_2_O_2_ production, as well as use with other photoanodes and biocatalysts (Supplementary Fig. 9).

Next, a large-area cathode composed of the Co–N/CNT catalyst was prepared on a piece of carbon paper for photo-electrocatalytic H_2_O_2_ production. The photo-electrocatalytic activities of the photoanode and cathode for water oxidation and the ORR, respectively, were tested in phosphate borate solution using linear sweep voltammetry (LSV) measurements. The operating current of the integrated photo-electrochemical cell could be estimated from the intersection of the LSV curves of the H:TiO_2_ photoanode and Co–N/CNT cathode (Fig. 2c). The intersection point in the LSV of H:TiO_2_ and Co–N/CNT was 0.62 mA, which far exceeds that of H:TiO_2_ and CoTMPP/CNT (0.09 mA), as well as those of H:TiO_2_ and pristine CNT (0.07 mA), highlighting the importance of an effective electrocatalyst for unassisted H_2_O_2_ production (Fig. 2c and Supplementary Fig. 10). To verify the stability of our integrated photo-electrochemical H_2_O_2_ production system, the amount of H_2_O_2_ generated on the Co–N/CNT cathode was estimated as a function of time using a colorimetric method with *N*,*N*-diethyl-*p*-phenylenediamine (DPD) (Fig. 2d). We observed continuous production of H_2_O_2_ over 6 h of reaction, and found that the H_2_O_2_ production rate can be easily increased by scaling up the size of electrodes and the reactor (Supplementary Fig. 11). In addition, the H_2_O_2_ produced at the cathode freely diffused into the biocatalyst cell through the cellulose membrane (Supplementary Fig. 12), enabling the utilisation of H_2_O_2_ by the biocatalyst for lignin depolymerisation and biopolymer synthesis.

### Photo-electro-biochemical lignin dimer depolymerisation

**Fig. 3:**
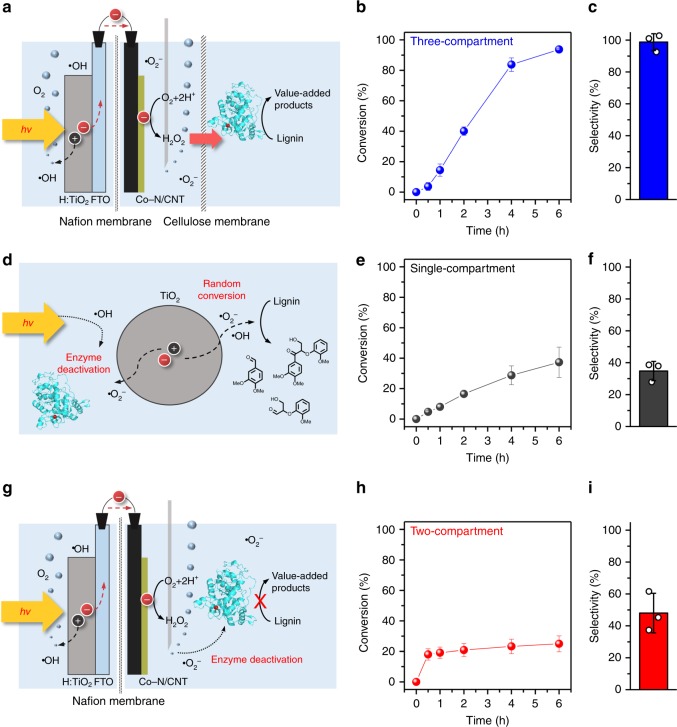
Reactor configurations and photo-electro-biochemical lignin dimer conversion. **a**, **d**, **g** Schematic representations of the three-compartment photo-electro-biochemical system (**a**), single-compartment powder-based photo-biochemical system (**d**), and two-compartment photo-electrochemical system (**g**) used for lignin dimer conversion. **b**, **e**, **h** Conversion and **c**, **f**, **i** selectivity of the three-compartment photo-electro-biochemical system (**b**, **c**), single-compartment powder-based photo-biochemical system (**e**, **f**), and two-compartment photo-electrochemical system (**h**, **i**). Error bars indicate the standard deviation

There are many reported lignin model compounds, such as ferulic acid, vanillyl alcohol isoeugenol, and benzyl alcohol^12,44^. We selected lignin dimer as a representative model compound to observe the specific cleavage of β-O-4. For the selective cleavage of the lignin dimer into 3,4-dimethoxybenzaldehyde (a derivative of vanillin), the LiPH8 biocatalyst was used, along with the photo-electrochemically generated H_2_O_2_ (Supplementary Fig. 13). Because our three-compartment cell design protects the biocatalyst from deactivation, the enzyme activity was maintained throughout the reaction (Fig. 3a and Supplementary Fig. 14). The depolymerisation conversion efficiency and selectivity for the lignin dimer were 93.7% and 98.7%, respectively, in the three-compartment photo-electro-biochemical system (Fig. 3b, c). A control reaction carried out in the absence of the LiPH8 enzyme in the three-compartment cell indicated low conversion and selectivity, suggesting that the biocatalyst is essential for selective lignin valorisation (Supplementary Fig. 15).

For comparison, we also tested lignin depolymerisation in single-compartment and two-compartment systems. In the single-compartment system, the powder-type TiO_2_ photocatalyst, the LiPH8 biocatalyst, and the lignin dimer were placed in the same compartment (Fig. 3d and Supplementary Fig. 1c). In this environment, lignin conversion can take place in two possible ways: direct lignin conversion by the charge carriers generated by the TiO_2_ photocatalyst or biocatalytic lignin conversion using LiPH8 with the aid of the photogenerated H_2_O_2_. The obtained lignin dimer conversion and selectivity were 37.3% and 34.8%, respectively (Fig. 3e, f). To investigate the role of the enzyme in the single-compartment system, we performed the lignin conversion experiment in the absence of LiPH8. Interestingly, a similar conversion and selectivity were obtained without LiPH8 (Supplementary Fig. 16) and very little H_2_O_2_ was generated in the single compartment (Supplementary Fig. 17), which indicates that the reaction mainly proceeds via direct photocatalytic lignin conversion by the TiO_2_ photocatalyst. The presence of many unidentified peaks using gas chromatography–mass spectroscopy (GC–MS) analysis also revealed that many side reactions take place upon the direct photocatalytic conversion of lignin (Supplementary Figs. 18–22). In the single-compartment system, solar light first excites the photocatalyst, generating holes and electrons that initiate the photochemical reaction and generate intermediate radical species (•OH and •O_2_^−^, respectively). These intermediate radicals can directly attack the biocatalyst, leading to a significant drop in activity. As for other proteins/enzymes, such as chymotrypsin, lysozyme, ribonuclease, and formate dehydrogenase, it was found that LiPH8 in this study also rapidly lost its activity upon irradiation and in the presence of bubbles of O_2_ gas (Supplementary Fig. 14)^45–47^. In particular, ultraviolet (UV) light directly destroys the protein structures^45^. Furthermore, gas bubbling also alters the protein structure upon adsorption of the biocatalyst at gas–liquid interfaces^46,47^. These factors (ROS, light, and O_2_ gas bubbling) are the main reasons for the low selectivity of the single-compartment system.

In the two-compartment system, the photoanode (H:TiO_2_) and electrocatalyst cathode (Co–N/CNT) were separated by a Nafion proton exchange membrane, but the cellulose membrane was removed between the cathode and biocatalyst parts (Fig. 3g and Supplementary Fig. 1d). This configuration prevents the direct photochemical lignin conversion observed in the single-compartment system and restrains the deactivation of LiPH8 biocatalyst by the photogenerated holes and •OH radicals in the anodic part. Furthermore, the remaining area was blackened and the H:TiO_2_ photoanode was covered from behind, as shown in Supplementary Fig. 1e, to shield the biocatalyst from UV light and inhibit its photo-deactivation as in the three-compartment system. The two-compartment cell showed increased selectivity of over 48% (Fig. 3i), which mainly arises from biocatalytic lignin conversion with the help of the in situ photogenerated H_2_O_2_ (Supplementary Fig. 23), because only 3% selectivity was obtained when parallel reaction was carried out in the absence of the LiPH8 biocatalyst (Supplementary Fig. 24). However, lignin dimer conversion was still very low (<25%) because the LiPH8 was exposed to O_2_ bubbles, which are detrimental to enzyme activity (Fig. 3h and Supplementary Fig. 14). Moreover, •O_2_^−^ radical intermediate generated during the O_2_ reduction also has an adverse effect on the LiPH8 enzyme activity.

In the three-compartment photo-electro-biochemical system, we verified that the concentrations of •OH and •O_2_^−^ radicals were very low in the enzyme cell compared to those in the anode and cathode cells, respectively (Supplementary Figs. 25 and 26), resulting in high conversion and selectivity of lignin dimer depolymerisation. The gradual increase of H_2_O_2_ concentration in the three-compartment cell is also a reason for the high performance, as this helps to maintain low concentrations of H_2_O_2_ in the enzyme cell. When the initial concentrations were 1 and 10 mM, the conversion efficiencies were 82% and 21%, respectively. These values are lower than that of three-compartment system (93.7%) because of enzyme deactivation due to high H_2_O_2_ concentration (Supplementary Fig. 27). When we fed a small amount of H_2_O_2_ every 15 min, the conversion efficiency was increased, indicating that the continuous and low concentration of H_2_O_2_ supply is preferred for the efficient biocatalytic lignin degradation (Supplementary Fig. 28). Turnover frequency (TOF) of the enzyme was 0.036 s^−1^, which is relatively lower than that of the LiPH8 enzyme reported in the literature^48^, due to the rate of lignin conversion being limited by H_2_O_2_ concentration (Supplementary Fig. 29). These results show that TOF can be controlled in a stable manner by adjusting the H_2_O_2_ generation speed of the photo-electro-biochemical system.

### Photo-electro-biochemical biopolymer synthesis

**Fig. 4:**
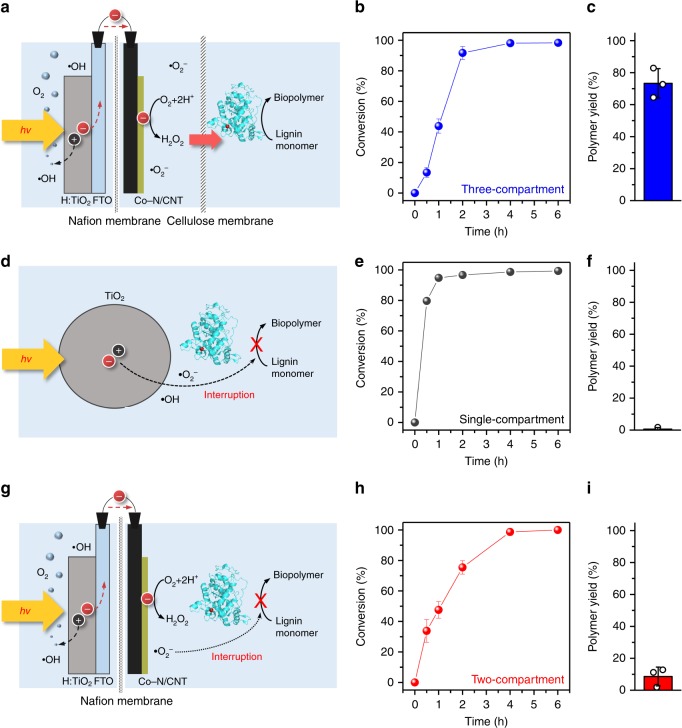
Reactor configurations and photo-electro-biochemical biopolymer synthesis. **a**, **d**, **g** Schematic representations of the: (**a**) three-compartment photo-electro-biochemical system, (**d**) single-compartment powder-based photo-biochemical system, and (**g**) two-compartment photo-electrochemical system used for biopolymer synthesis. **b**, **e**, **h** Conversion percentage of coniferyl alcohol over time and **c**, **f**, **i** polymer yield after 6 h of reaction of the three-compartment photo-electro-biochemical system (**b**, **c**), single-compartment powder-based photo-biochemical system (**e**, **f**), and two-compartment photo-electrochemical system (**h**, **i**). Error bars indicate the standard deviation

The three different types of reactors were also applied for the biopolymer synthesis using coniferyl alcohol, which is one of the three major units of lignin (Supplementary Fig. 30)^49^. Because current methods of polymer production depend on petrochemicals and, thus, have unavoidable negative environmental effects, polymer synthesis using the abundant, carbon neutral, and recalcitrant lignin biomass is a promising alternative. In the three-compartment photo-electro-biochemical system, biopolymer synthesis reaction was performed with horseradish peroxidase (HRP) biocatalyst with in situ solar H_2_O_2_ generation at ambient temperature and pressure (Fig. 4a). The conversion efficiency of coniferyl alcohol monomer and the yield of the biopolymer were 98.3% (Fig. 4b) and 73.3% (Fig. 4c), respectively.

This polymer was comprised of the common linkages between coniferyl alcohol molecules, such as β-O-4, β-β and β-5, and has a number average molecular weight of 1103, which were confirmed by two-dimensional-nuclear magnetic resonance spectroscopy (2D-NMR, Supplementary Fig. 31), and gel permeation chromatography, respectively.

Unlike the three-compartment system, the single-compartment and two-compartment systems yielded negligible amounts of biopolymers at the end of the reaction (Fig. 4f, i) but a large number of by-products were formed (Supplementary Figs. 32 and 33), although > 90% of the monomeric substrate was consumed during the dehydrogenative polymerisation reaction (Fig. 4e, h). Because the HRP biocatalyst shows good stability against O_2_ bubbling and solar irradiation (Supplementary Fig. 34), the low selectivity for polymerisation may originate from the generation of highly reactive •OH and •O_2_^−^ reaction intermediates on the TiO_2_ photocatalyst surface (Fig. 4d). These ROS can lead to further oxidation or the disproportionation of phenolic radicals rather than radical coupling and selective polymerisation (Supplementary Fig. 30)^50^. Thus, the majority of the products in the single-compartment reactor are soluble organic compounds rather than biopolymers (Supplementary Figs. 32 and 33). In the case of the two-compartment cell, even though •OH radicals do not interrupt the biochemical reactions, the presence of •O_2_^−^ radicals and O_2_-coordinating cobalt porphyrin form a superoxide-like structure^51^, which can disrupt the polymerisation reaction/biopolymer formation (Fig. 4g). This leads to the formation of a significant amount of soluble by-products, although the amount produced is lower than that produced in the single-compartment system (Supplementary Figs. 32 and 33). A variety of by-products were detected in the GC–MS analysis (Supplementary Figs. 32 and 33). This indicates that the formation of undesirable highly reactive •O_2_^−^ and •OH radicals in these systems resulted in low-molecular-weight by-products rather than selective polymerisation. However, in the three-compartment photo-electro-biochemical system, the interaction between ROS radicals and the substrate was limited by separating the electrocatalytic (cathodic) and biocatalytic compartments with a cellulose membrane, as experimentally demonstrated earlier (Supplementary Figs. 25 and 26). This results in the selective oxidation of phenolic compounds, and successive radical coupling leads to a high polymer yield. Control reactions performed in the absence of the HRP biocatalyst using all three systems showed no polymer product formation, demonstrating the pivotal role of biocatalyst for biopolymer synthesis (Supplementary Figs. 35–37).

## Discussion

In summary, we have demonstrated that a compartmented photo-electro-biochemical system that integrates a photocatalyst, an electrocatalyst, and a biocatalyst, is effective for selective and stable lignin dimer valorisation under solar irradiation without the need for any additional bias or sacrificial agent. In this design, the placement of appropriate membranes as separators between cells protects the biocatalyst from detrimental conditions generated during the reaction, thus preserving its stability and activity. This photo-electro-biochemical system can catalyse lignin dimer cleavage with a 93.7% conversion efficiency and 98.7% selectivity, which far surpasses those of single-compartment (37.3% and 34.8%) and two-compartment (25.0%, 48.1%) systems. The system was further applied for sustainable polymer synthesis using a lignin monomer, coniferyl alcohol, with a 73.3% yield and 98.3% of conversion efficiency; however, the polymer yields of the single-compartment and the two-compartment systems were only ca. 0% and 8.6%, respectively. This unassisted selective lignin valorisation technology could convert waste lignin to value-added aromatics and polymer without the need for any additional energy and chemicals, possibly overcoming the problems associated with current biomass upgradation, such as its low cost effectiveness and limited processing technology. We believe that the development and scaling-up of this technology will be a milestone for the replacement of petrochemicals with biochemicals.

## Methods

### Preparation of TiO_2_ photoanode

A rutile TiO_2_ nanowire film was hydrothermal grown on FTO glass^37^. First, 15 mL of hydrochloric acid (35%, Samchun Chemical) was diluted with 15 mL deionised (DI) water and mixed with 0.5 mL titanium (IV) butoxide (97%, Aldrich) in a 100 mL beaker. This clear solution and a clean FTO glass substrate were transferred to a Teflon-lined stainless-steel autoclave (125 mL). The sealed autoclave was heated in an oven at 150 °C for 5 h and then cooled to room temperature (RT) slowly. After rinsing with DI water, calcination was performed at 550 °C for 5 h to increase the crystallinity. Finally, the sample was annealed in a hydrogen atmosphere at 350 °C for 30 min^38^. In addition, an epoxy resin was added behind the TiO_2_ electrode to block the light transmitted through the TiO_2_ photoanode. A planar TiO_2_ film was made on FTO substrate using a radiofrequency sputtering device (SRN-120, SORONA) and calcination was performed at 550 °C for 5 h.

### Characterisation of TiO_2_/FTO

XRD patterns of TiO_2_ and H:TiO_2_ films were recorded using a PANalytical X’Pert PRO diffractometer from 10° to 70° in 2*θ* at a scanning speed of 1° min^−1^. SEM images were obtained using a Cold FE-SEM (SU8220, Hitach High-Technologies). Optical properties were determined by UV–vis diffuse reflectance spectroscopy using a UV/Vis/NIR spectrophotometer (UV-3600, Shimadzu).

### Synthesis of acid-treated carbon nanotubes (AT-CNT)

Before catalyst synthesis, the CNTs were acid-treated to remove metallic impurities. First, 10.0 g of multi-walled CNTs (MR99, Carbon Nanotech Co., LTD) and 715 g of 6 M HNO_3_ (diluted from 60 wt% HNO_3_, Samchun Chemical) were mixed and stirred at 80 °C for 12 h. The CNT slurry was filtered, washed with copious amounts of DI water, and dried at 60 °C. The HNO_3_-treated CNTs were subsequently washed with 700 g of 6 M HCl (diluted from 36 wt% HCl, Samchun Chemical) in the same manner.

### Synthesis of the Co–N/CNT catalyst

First, 0.75 g of AT-CNT and 1.50 g of 5,10,15,20-tetrakis(4-methoxyphenyl)-21*H*,23*H*-porphine cobalt(II) (CoTMPP, 98%, Porphyrin Systems) were mixed in an agate mortar for 15 min. The mixture was heated at 400 °C under 1 L min^−1^ N_2_ flow for 3 h (ramping rate: ca. 2.1 °C min^−1^). The heat-treated powder and 3.75 mL of tetraethyl orthosilicate (TEOS, 98%, Aldrich) were mixed in the mortar for 5 min. The same volume of formic acid (99.5%, Samchun Chemical) was added to the mixture to initiate TEOS polymerisation. The mixture was dried at 60 °C for 3 h. The dried solid was ground to fine powder and a part (0.60 g) was pyrolyzed at 900 °C under 1 L min^−1^ N_2_ flow for 3 h (ramping rate: ca. 2.1 °C min^−1^). To etch the silica and acid-soluble Co-species, the pyrolyzed powders were added to an acid solution of 4 M HF + 2 M HCl (diluted from 50 wt% HF (JT Baker) and 36 wt% HCl) with a similar volume of ethanol (94.5%, Samchun Chemical) and stirred at RT for 30 min. The suspension was filtered and washed with ethanol and DI water. Acid leaching was repeated once more in the same manner. Finally, the product was dried at 60 °C and collected. Then, the Co–N/CNT-based cathode was prepared by drop-casting catalyst ink on both sides of a carbon paper (0.2 mg cm^−2^).

### Synthesis of the CoTMPP/CNT catalyst

First, 75 mg of AT-CNT was dispersed in 50 mL of *N*,*N*-dimethylformamide (DMF, 99.5%, Samchun Chemical) and ultrasonicated for 1 h. During the ultrasonication, CoTMPP in a DMF solution was prepared (10 mg mL^−1^). The CoTMPP solution (2.3 mL; 23 mg of CoTMPP) was added to the AT-CNT suspension, ultrasonicated for 1 h, and stirred at RT for 12 h. The suspension was centrifuged at 8000 rpm and the supernatant was decanted. The precipitate was washed with DMF twice and anhydrous ethanol and dried at 60 °C. The nominal Co loading was 1 wt%.

### Characterisation of Co–N/CNT

XPS measurements were performed with a K-Alpha X-ray photoelectron spectrometer (Thermo Fisher Scientific) with a monochromatic Al *K*_α_ X-ray source (1486.6 eV). Co 2*p* and N 1*s* XPS spectra were deconvoluted using the XPSPeak41 software with the mixed (Gaussian 70, Lorentzian 30)-function after a Shirley-type background correction. The Co content in Co–N/CNT catalyst was analysed using an inductively coupled plasma-optical emission spectrometer (700-ES, Varian). STEM images were obtained using a JEOL JEM-2100F microscope at an acceleration voltage of 200 kV.

### X-ray absorption spectroscopy

X-ray absorption spectroscopy (XAS) was performed at beamline 6D of the Pohang Accelerator Laboratory. The storage ring was operated at an energy of 3 GeV and a beam current of 360 mA. The incident photons were monochromated by Si(111) double crystal, detuned by 30% to remove high-order spectral contamination, and calibrated using a standard Co foil. Co–N/CNT powder sample was pressed using a hand-held pelletiser. Finally, XAS spectra were obtained in transmission detection mode. We also measured the XAS spectrum of CoTMPP. The resulting XAS data were treated using the Athena programme to remove the background and for normalisation^52^.

### Electrochemical characterisation (RRDE)

Electrochemical measurements were conducted using an electrochemical workstation (CHI760E, CH Instruments). The three-electrode system was built for measurement using a graphite rod counter electrode, an Ag/AgCl (saturated KCl filled, RE-1B, ALS) reference electrode, and a catalyst-loaded RRDE (AFE7R9GCPT, Pine Research Instrumentation). Before each use, the RRDE was polished with aqueous 1.0 and 0.3 μm alumina suspensions and a microcloth. The Co–N/CNT catalyst ink was prepared by mixing 10 mg of catalyst, 100 μL of DI water, 13 μL of Nafion dispersion (5 wt%, D521, Dupont), and 1085 μL of anhydrous ethanol. The CoTMPP/CNT catalyst ink was prepared in the same manner but diluted by addition of 300 μL of DI water and 3300 μL of ethanol. The catalyst inks were homogenised using an ultrasonic bath. Then, 6 μL of the catalyst ink was deposited onto the RRDE disk (24 μL for the CoTMPP/CNT catalyst). The resulting catalyst loading was 0.2 mg cm^−2^. Electrochemical measurements were made both in the 0.1 M phosphate borate solution and 0.1 M HClO_4_ (diluted from 70% Veritas double distilled, GFS chemicals). After soaking the catalyst-loaded RRDE into a N_2_-saturated electrolyte, cyclic voltammetry (CV) was performed between 0.05 and 1.20 V (vs. RHE) at a scan rate of 100 mV s^−1^. Steady CV responses were observed within 20 cycles. The Pt ring of the RRDE was electrochemically cleaned in the same potential range with a scan rate of 500 mV s^−1^ for 10 cycles. Electrochemical impedance spectroscopy measurement was carried out at 0.68 V (vs. RHE) from 100,000 to 1 Hz at an electrode rotation speed of 1600 rpm in an O_2_-saturated electrolyte. The series resistance was determined at the high-frequency tail of the Nyquist plot for *iR*-compensation. The ORR polarisation curve was obtained by LSV from 1.2 to 0.2 V (vs. RHE) at a scan rate of 5 mV s^−1^ and at different electrode rotation speeds of 2025, 1600, 1225, and 900 rpm for Koutecky–Levich analysis, as shown below.

1 $$\frac{1}{{i}} = \frac{1}{{{i}_{\mathrm{{{k}}}}}} + \frac{1}{{0.62{nFAD}_{O}^{2/3}{v}^{ - 1/6}{C}_{O}{\omega }^{1/2}}} = \frac{1}{{{i}_{\mathrm{{{k}}}}}} + \frac{1}{{{B} \times {\omega }^{1/2}}}$$

Here, *i*, *i*_k_, *n*, *F*, *A*, *D*_O_, *v*, *C*_O_, and *ω* represent the measured current, the kinetic current, the electron transfer number, the Faraday constant (96,485 C mol^−1^), the diffusion coefficient of O_2_, the kinematic viscosity, the O_2_ concentration, and the electrode rotation speed, respectively. The plot of *i*^−1^ as a function of *ω*^−1/2^ gives a line with a slope of *B*^**−1**^, which is used to calculate *n*. Note that *D*_O_, *v*, and *C*_O_ values vary with the electrolyte: *D*_O_ (1.90 × 10^−5^ and 1.93 × 10^−5^ cm^2^ s^−1^), *v* (0.010 and 0.0101 cm^2^ s^−1^), and *C*_O_ (1.21 and 1.26 × 10^−6^ mol cm^−3^) for 0.1 M phosphate borate solution and 0.1 M HClO_4_, respectively^53,54^.

To measure H_2_O_2_ yield, the Pt ring potential was held at 1.3 V (vs. RHE) during the LSV measurements, and the H_2_O_2_ selectivity was then calculated according to the following equation,

2 $${{\mathrm{{H}}}}_2{{\mathrm{{O}}}}_2\,{{\mathrm{{selectivity}}}}\,\left( {\mathrm{\% }} \right) = \frac{{200}}{{1 + \frac{{{{N}} \times {i}_{{\mathrm{{d}}}}}}{{{i}_{{\mathrm{{r}}}}}}}}$$

where *i*_d_, *i*_r_, and *N* indicate the disk current, the ring current, and the collection efficiency (37%, provided by the manufacturer), respectively. To assess the durability of the catalysts, potential cycling tests were performed between 0.6 and 1.0 V (vs. RHE) with an electrode rotation of 1600 rpm at a scan rate of 50 mV s^−1^ for 1000 cycles in the O_2_-saturated electrolyte. After cycling, the ORR activity was measured in a fresh electrolyte.

### (Photo-)electrochemical measurements

LSV curves of the H:TiO_2_ were recorded using a digital multimeter (Ivium-n-Stat Multichannel potentiostat) with the Ag/AgCl reference electrode and a Pt wire counter electrode in 0.1 M phosphate borate solution at pH 4.5 from 0 to 2.00 V (vs. RHE) at a scan rate of 5 mV s^−1^. The front side of the photoanode (1.33 cm^2^) was illuminated with the solar simulator (10500, Abet Technologies), and the light intensity was adjusted to 100 mW cm^−2^ (AM1.5 G) at the sample position using a standard Si cell (PEC-SI01, Peccell Technologies, Inc.). To measure photoanode stability, the chronoamperometric current of H:TiO_2_ was measured at a constant applied voltage of 1.23 V (vs. RHE) during 12 h. LSV curves of the Co–N/CNT-based cathode (2.0 cm^2^) were also recorded from 1.2 to 0.05 V (vs. RHE) at a scan rate of 5 mV s^−1^ in O_2_-saturated 0.1 M phosphate borate solution at pH 4.5.

### Hydrogen peroxide detection

The concentration of H_2_O_2_ was estimated using the DPD method^55^. Depending on the concentration of produced H_2_O_2_, the samples were diluted with 0.1 M phosphate borate solution at pH 4.5 or 6.0 in the cases of lignin degradation and biopolymer synthesis, respectively, to avoid exceeding the detection limit of the DPD method. In the case of the powder system, the sample was filtered using a 0.45-μm polytetrafluoroethylene (PTFE) filter (SLCR013NL, Millipore). Furthermore, 0.05 g of *N*,*N*-diethyl-1,4-phenylene-diamine sulphate (DPD, ≥ 98.0%, Aldrich) was dissolved in 5 mL of 0.1 N H_2_SO_4_ and stored in the dark at 5 °C and 5 mg of peroxidase (POD, horseradish, Sigma) was dissolved in 5 mL DI water and kept at 5 °C. Finally, 2.7 mL of a 0.1 M sodium phosphate buffer (pH 6.0), 0.05 mL of DPD solution, 0.05 mL of POD solution, and 0.2 mL of sample were mixed. The absorbance of mixed solution at *λ* = 551 nm was measured using a UV/visible spectrophotometer (UV-2600, Shimadzu).

### Enzyme stability investigation

To determine the stability of the enzymes against light irradiation and O_2_ gas purging, LiPH8 and HRP enzymes in 0.1 M phosphate borate solution at pH 4.5 and 6.0 were subject to the same light irradiation and O_2_ gas purging as the actual lignin conversion experiments. Three sets of experiments (viz., without light irradiation and O_2_ purging, with light irradiation, and with O_2_ bubbling) were performed. The residual activity of the enzyme was measured at several points during the 3-h experiment using the oxidation activity of 189 µM 2,2′-azino-bis(3-ethylbenzthiazoline-6-sulfonic acid (ABTS) in the presence of 250 μM H_2_O_2_ and 0.1 M Britton–Robinson buffer of pH 3.0. The formation of the product was recorded at 420 nm within 1 min with *Ɛ*_420 nm_ = 36.7 mM^−1^ cm^−1^ using the UV/visible spectrophotometer.

### Detection of hydroxyl/superoxide radicals

The formation of •OH was measured using a fluorescence probe method with coumarin^56^. 0.2 mM coumarin (Aldrich) was added to the 0.1 M phosphate borate solution at the anode and enzyme cell in the three-compartment reactor, and the fluorescence spectra were measured using a fluorescence spectrophotometer (Cray Eclipse, Varian) with the excitation wavelength at 332 nm. Coloration of XTT reduction to XTT–formazam was used for •O_2_^−^ detection^57,58^. For this, 0.1 M phosphate borate solution containing 0.1 mM 2,3-bis(2-methoxy-4-nitro-5-sulfophehyl)-2*H*-tetrazolium-5-carboxanilide (XTT, Aldrich) was added in the cathode and enzyme cell, and the absorbance spectra were recorded from 650 nm to 350 nm using the UV/visible spectrophotometer.

### Enzyme preparation

The LiPH8 synthetic gene, including the seven-residue pro-sequence, was synthesised by Bioneer Company (South Korea). The gene coding protein sequence was retrieved from UniProtKB database (P06181). LiPH8 was expressed as inclusion body in *Escherichia coli* BL21 (DE3) and reactivated through in vitro refolding procedure as previously reported^59^ with slight modification. The inclusion body was added into the refolding solution containing 100 mM Tris–HCl pH 8.0, CaCl_2_ 2 mM, guanidine hydrochloride 0.36 M, l-glutathione oxidised form 0.7 mM and stirred overnight at 4 °C. The refolded solution was concentrated and buffer-exchanged with sodium acetate buffer 100 mM at pH 4.0 then pH 6.0 before subjected for purification. The anion-exchange chromatography with Mono Q 5/50 GL column (GE Healthcare Life Sciences Co., USA) was used for purification step in this study. The column was equilibrated with sodium acetate 10 mM, pH 6.0 (buffer A) and eluted with a linear gradient of buffer B—sodium acetate 500 mM, pH 6.0. The highest activity fraction of LiPH8 was tested with sodium dodecyl sulfate–polyacrylamide gel electrophoresis and used for this study. HRP type VI was purchased from Aldrich Co. (USA) and used without any further purification.

### Photo-electro-biochemical lignin conversion

The overall reaction was performed in an acryl reactor composed of an anode cell with a quartz window on one side, a cathode cell, and an enzyme cell. In the case of the three-compartment reactor, the anode cell was separated from cathode cell through a Nafion membrane (Nafion^®^ 117, 0.18 mm thick, Aldrich), and the cathode cell was separated from an enzyme cell through a cellulose membrane (Spectra/Por^®^, 6–8 kDa, Spectrum). For the lignin dimer conversion, 0.1 M phosphate borate solution of pH 4.5 was used as the electrolyte. The anode cell, cathode cell, and enzyme cell consisted of H:TiO_2_ photoanode (1.33 cm^2^) with 8 mL electrolyte, Co–N/CNT-based cathode (2.0 cm^2^) with 4 mL electrolyte, and 0.5 mM of lignin dimer and 0.8 μM of LiPH8 in 8 mL electrolyte, respectively. The photoanode and cathode were connected to each other with alligator clips and copper wire as an external circuit. In the case of the two-compartment reactor without an enzyme cell, the cathode cell was composed of the Co–N/CNT cathode, 0.5 mM of lignin dimer, and 0.8 μM of the LiPH8 in 4 mL electrolyte. In the single cell, Degussa P25 TiO_2_ powder (0.5 mg mL^−1^) used as a photocatalyst for H_2_O_2_ production, 0.5 mM of lignin dimer, and 0.8 μM of the LiPH8 enzyme were added together in 8 mL electrolyte. The photoanode (or P25 photocatalyst) was illuminated using a solar simulator (10500, Abet Technologies) at 100 mW cm^−2^ (AM1.5 G). O_2_ gas was continuously bubbled into the cathode electrolyte from 20 min before the reaction until the reaction was completed. The biopolymer synthesis was performed in the three kinds of the reactors as depolymerisation of lignin with slight modifications. The reactions were carried out with 0.5 mM of coniferyl alcohol as a substrate and 1.4 μM of the HRP as an enzyme in 0.1 M phosphate borate solution at pH 6.0.

### Identification of phenolic compounds

The completed reaction mixture was analysed by high-performance liquid chromatography (HPLC). The HPLC procedure was performed by injecting fractions using an Agilent 1200 HPLC system onto a reverse-phase Eclipse XDB-C18 column (4.6 × 150 mm, 5 μm, Agilent). Gradient separation was performed using 0.1% aqueous trifluoroacetic acid (solvent A) to methanol/acetonitrile (25:75; v/v; solvent B) with the following conditions: analysis time of 15 min flow of 1.5 mL min^−1^ and column temperature of 30 °C. The gradient programme was as follows: 0 min—15% B, 6 min—60% B, 11.5 min—100% B, and 13 min—0% B. Before injection, the sample was filtered through a hydrophilic 0.2-μm PTFE membrane filter. The products were identified relative to an authentic library of standards based on their retention times and UV absorption spectra. The quantification of the reaction was performed by HPLC based on a linear external standard curve (*R*^2^ > 0.95) of the respective compound. For the by-products, the phenolic derivatives were extracted with dichloromethane and analysed using a GC–MS (Agilent) with a DB-5MS (60 m × 250 μm × 0.25 μm) column. The oven temperature was elevated from 50 to 280 °C. The product mass spectrum was identified using an authentic library of standards.

### Characterisation of the polymer

The polymer was collected by centrifugation at 13,000 rpm within 10 min, washed three times with 2 N HCl solution, and dried under vacuum conditions. The polymer yield was determined by UV analysis at 280 nm based on a linear external standard curve (*R*^2^ > 0.95) of the respective synthesised dehydrogenative polymer from coniferyl alcohol. The dried polymer was dissolved in tetrahydrofuran (THF) at a concentration of 3 mg mL^−1^. The solution was filtered using a 0.2 μm PTFE filter and analysed by gel permeation chromatography, for which THF was used as eluent with a flow rate of 1 mL min^−1^. Samples were detected at 280 nm and polystyrene standard used for calibration curves. For 2D ^13^C–^1^H heteronuclear single quantum correlation (HSQC) analysis, the polymer was dissolved in 0.5 mL DMSO-d_6_ before NMR analysis. NMR spectra from the polymer were acquired on a 400 MHz FT-NMR (Bruker) instrument with a 5 mm BBO NMR probe. The central DMSO solvent peak was used as an internal reference (δH/δC 2.50/39.52 ppm). The HSQC parameters and NMR chemical assignments followed previously published methods^60^.

## Supplementary information

Supplementary Information

Peer Review

## Data Availability

All data are available from the corresponding author upon request.

## References

[1] Hoffert MI (2002). Advanced technology paths to global climate stability: energy for a greenhouse planet. Science.

[2] Ragauskas AJ (2006). The path forward for biofuels and biomaterials. Science.

[3] Thompson P (2012). The agricultural ethics of biofuels: the food vs. fuel debate. Agriculture.

[4] Calvo-Flores FG, Dobado JA (2010). Lignin as renewable raw material. ChemSusChem.

[5] Zakzeski J, Bruijnincx PCA, Jongerius AL, Weckhuysen BM (2010). The catalytic valorization of lignin for the production of renewable chemicals. Chem. Rev..

[6] Azadi P, Inderwildi OR, Farnood R, King DA (2013). Liquid fuels, hydrogen and chemicals from lignin: a critical review. Renew. Sustain. Energy Rev..

[7] Ragauskas AJ (2014). Lignin valorization: improving lignin processing in the biorefinery. Science.

[8] Beckham GT, Johnson CW, Karp EM, Salvachúa D, Vardon DR (2016). Opportunities and challenges in biological lignin valorization. Curr. Opin. Biotechnol..

[9] Rinaldi R (2016). Paving the way for lignin valorisation: recent advances in bioengineering, biorefining and catalysis. Angew. Chem. Int. Ed..

[10] Schutyser W (2018). Chemicals from lignin: an interplay of lignocellulose fractionation, depolymerisation, and upgrading. Chem. Soc. Rev..

[11] Sun Z, Fridrich B, de Santi A, Elangovan S, Barta K (2018). Bright side of lignin depolymerization: toward new platform chemicals. Chem. Rev..

[12] Li S-H, Liu S, Colmenares JC, Xu Y-J (2016). A sustainable approach for lignin valorization by heterogeneous photocatalysis. Green Chem..

[13] Rahimi A, Azarpira A, Kim H, Ralph J, Stahl SS (2013). Chemoselective metal-free aerobic alcohol oxidation in lignin. J. Am. Chem. Soc..

[14] Rahimi A, Ulbrich A, Coon JJ, Stahl SS (2014). Formic-acid-induced depolymerization of oxidized lignin to aromatics. Nature.

[15] Van den Bosch S (2015). Reductive lignocellulose fractionation into soluble lignin-derived phenolic monomers and dimers and processable carbohydrate pulps. Energy Environ. Sci..

[16] Tien M, Kirk TK (1983). Lignin-degrading enzyme from the hymenomycete *Phanerochaete chrysosporium* burds. Science.

[17] Shuai L (2016). Formaldehyde stabilization facilitates lignin monomer production during biomass depolymerization. Science.

[18] Silva GGD, Couturier M, Berrin J-G, Buléon A, Rouau X (2012). Effects of grinding processes on enzymatic degradation of wheat straw. Bioresour. Technol..

[19] Li H, Qu Y, Yang Y, Chang S, Xu J (2016). Microwave irradiation—a green and efficient way to pretreat biomass. Bioresour. Technol..

[20] Yuan Z (2015). Process intensification effect of ball milling on the hydrothermal pretreatment for corn straw enzymolysis. Energy Convers. Manag..

[21] Wu M (2015). Separation and characterization of lignin obtained by catalytic hydrothermal pretreatment of cotton stalk. Ind. Crops Prod..

[22] Floudas D (2012). The Paleozoic origin of enzymatic lignin decomposition reconstructed from 31 fungal genomes. Science.

[23] Smith AT, Doyle WA, Dorlet P, Ivancich A (2009). Spectroscopic evidence for an engineered, catalytically active Trp radical that creates the unique reactivity of lignin peroxidase. Proc. Natl Acad. Sci. USA.

[24] Saez-Jimenez V (2015). Demonstration of lignin-to-peroxidase direct electron transfer: a transient-state kinetics, directed mutagenesis, EPR, and NMR study. J. Biol. Chem..

[25] Sáez-Jiménez V (2016). Role of surface tryptophan for peroxidase oxidation of nonphenolic lignin. Biotechnol. Biofuels.

[26] Campos-Martin JM, Blanco-Brieva G, Fierro JLG (2006). Hydrogen peroxide synthesis: an outlook beyond the anthraquinone process. Angew. Chem. Int. Ed..

[27] Edwards JK (2009). Switching off hydrogen peroxide hydrogenation in the direct synthesis process. Science.

[28] Liu Y, Chen F, Wang Q, Wang J, Wang J (2018). Direct unassisted hydrogen peroxide generation from oxygen and water on plasmonic Ag–graphene–Cu nanosandwitch. Appl. Catal. B.

[29] Fukuzumi S, Yamada Y, Karlin KD (2012). Hydrogen peroxide as a sustainable energy carrier: electrocatalytic production of hydrogen peroxide and the fuel cell. Electrochim. Acta.

[30] Seh ZW (2017). Combining theory and experiment in electrocatalysis: insights into materials design. Science.

[31] Sa YJ, Kim JH, Joo SH (2019). Active edge-site-rich carbon nanocatalysts with enhanced electron transfer for efficient electrochemical hydrogen peroxide production. Angew. Chem. Int. Ed..

[32] Zhang W (2017). Selective activation of C−H bonds in a cascade process combining photochemistry and biocatalysis. Angew. Chem. Int. Ed..

[33] Zhang W (2018). Selective aerobic oxidation reactions using a combination of photocatalytic water oxidation and enzymatic oxyfunctionalizations. Nat. Catal..

[34] van Schie MMCH (2019). Cascading g-C_3_N_4_ and peroxygenases for selective oxyfunctionalization reactions. ACS Catal..

[35] Willot SJP (2019). Expanding the spectrum of light-driven peroxygenase reactions. ACS Catal..

[36] Zhang N, Yang M-Q, Liu S, Sun Y, Xu Y-J (2015). Waltzing with the versatile platform of graphene to synthesize composite photocatalysts. Chem. Rev..

[37] Liu B, Aydil ES (2009). Growth of oriented single-crystalline rutile TiO_2_ nanorods on transparent conducting substrates for dye-sensitized solar cells. J. Am. Chem. Soc..

[38] Wang G (2011). Hydrogen-treated TiO_2_ nanowire arrays for photoelectrochemical water splitting. Nano Lett..

[39] Anson FC, Ni CL, Saveant JM (1985). Electrocatalysis at redox polymer electrodes with separation of the catalytic and charge propagation roles. reduction of dioxygen to hydrogen peroxide as catalyzed by cobalt(II) tetrakis(4-N-methylpyridyl)porphyrin. J. Am. Chem. Soc..

[40] Claude E, Addou T, Latour J-M, Aldebert P (1998). A new method for electrochemical screening based on the rotating ring disc electrode and its application to oxygen reduction catalysts. J. Appl. Electrochem..

[41] Bezerra CWB (2008). A review of Fe–N/C and Co–N/C catalysts for the oxygen reduction reaction. Electrochim. Acta.

[42] Sa YJ (2016). A general approach to preferential formation of active Fe–N_*x*_ sites in Fe–N/C electrocatalysts for efficient oxygen reduction reaction. J. Am. Chem. Soc..

[43] Sa YJ (2019). Heterogeneous Co–N/C Electrocatalysts with controlled cobalt site densities for the hydrogen evolution reaction: structure–activity correlations and kinetic insights. ACS Catal..

[44] Augugliaro V (2012). Synthesis of vanillin in water by TiO_2_ photocatalysis. Appl. Catal. B.

[45] McLaren AD, Luse RA (1961). Mechanism of inactivation of enzyme proteins by ultraviolet light. Science.

[46] Caussette, M., Gaunand, A., Planche, H. & Lindet, B. in *Progress in Biotechnology*, Vol. 15 (eds Ballesteros, A., Plou, F. J., Iborra, J. L. & Halling, P. J.) 393–398 (Elsevier, 1998).

[47] Bommarius AS, Karau A (2005). Deactivation of formate dehydrogenase (FDH) in solution and at gas–liquid interfaces. Biotechnol. Prog..

[48] Pham LTM, Kim SJ, Kim YH (2016). Improvement of catalytic performance of lignin peroxidase for the enhanced degradation of lignocellulose biomass based on the imbedded electron-relay in long-range electron transfer route. Biotechnol. Biofuels.

[49] Li Q (2015). Dehydrogenative polymerization of coniferyl alcohol in artificial polysaccharides matrices: effects of xylan on the polymerization. J. Agric. Food Chem..

[50] Awungacha Lekelefac C, Busse N, Herrenbauer M, Czermak P (2015). Photocatalytic based degradation processes of lignin derivatives. Int. J. Photoenergy.

[51] Zhao Z, Ozoemena KI, Maree DM, Nyokong T (2005). Synthesis and electrochemical studies of a covalently linked cobalt(II) phthalocyanine–cobalt(II) porphyrin conjugate. Dalton Trans..

[52] Ravel B, Newville M (2005). ATHENA, ARTEMIS, HEPHAESTUS: data analysis for X-ray absorption spectroscopy using IFEFFIT. J. Synchrotron Radiat..

[53] Xing, W., Yin, G. & Zhang, J. *Rotating Electrode Methods And Oxygen Reduction Electrocatalysts* (Elsevier, 2014).

[54] Marković NM, Gasteiger HA, Grgur BN, Ross PN (1999). Oxygen reduction reaction on Pt(111): effects of bromide. J. Electroanal. Chem..

[55] Bader H, Sturzenegger V, Hoigné J (1988). Photometric method for the determination of low concentrations of hydrogen peroxide by the peroxidase catalyzed oxidation of N,N-diethyl-p-phenylenediamine (DPD). Water Res..

[56] Zhang J, Nosaka Y (2013). Quantitative detection of OH radicals for investigating the reaction mechanism of various visible-light TiO_2_ photocatalysts in aqueous suspension. J. Phys. Chem. C.

[57] Sutherland MW, Learmonth BA (1997). The tetrazolium dyes MTS and XTT provide new quantitative assays for superoxide and superoxide dismutase. Free Radic. Res..

[58] Li Y, Zhang W, Niu J, Chen Y (2012). Mechanism of photogenerated reactive oxygen species and correlation with the antibacterial properties of engineered metal-oxide nanoparticles. ACS Nano.

[59] Doyle WA, Smith AT (1996). Expression of lignin peroxidase H8 in *Escherichia coli*: folding and activation of the recombinant enzyme with Ca^2+^ and haem. Biochem. J..

[60] Kim H, Ralph J (2010). Solution-state 2D NMR of ball-milled plant cell wall gels in DMSO-*d*_6_/pyridine-*d*_5_. Org. Biomol. Chem..

