# Supplementary material for: Unassisted solar lignin valorisation using a compartmented photo-electro-biochemical cell
Source: Nat Commun. 2019 Nov 12;10:5123. doi: 10.1038/s41467-019-13022-7 (PMC6851146; doi:10.1038/s41467-019-13022-7)
Supplement: Supplementary file 1 — Supplementary Information [file 41467_2019_13022_MOESM1_ESM.pdf]

Supplementary Information

Unassisted solar lignin valorisation using a compartmented photo-electro-biochemical cell

Myohwa Ko[1,5], Le Thanh Mai Pham[1,5], Young Jin Sa[1,4,5], Jinwoo Woo[1,2], Trang Vu Thien Nguyen[1], Jae Hyung Kim[1,2], Dongrak Oh[1], Pankaj Sharma[1], Jungki Ryu[1,2], Tae Joo Shin[3], Sang Hoon Joo[1,2,*], Yong Hwan Kim[1,*], and Ji-Wook Jang[1,*]

[1]School of Energy and Chemical Engineering, Ulsan National Institute of Science and Technology (UNIST), Ulsan 44919, Republic of Korea

[2]Department of Energy Engineering, Ulsan National Institute of Science and Technology (UNIST), Ulsan 44919, Republic of Korea

[3]UNIST Central Research Facilities & School of Natural Science, Ulsan National Institute of Science and Technology (UNIST), 50 UNIST-gil, Ulsan 44919, Republic of Korea

[4]Present address: Department of Chemistry, Kwangwoon University, 20 Gwanwoon-ro, Nowon-gu, Seoul 01897, Republic of Korea

[5]These authors contributed equally: Myohwa Ko, Le Thanh Mai Pham, and Young Jin Sa

*Email: jiwjang@unist.ac.kr (J.-W.J.); metalkim@unist.ac.kr (Y.H.K.); shjoo@unist.ac.kr (S.H.J.)

Supplementary note 1

The electrochemical equation for two-electron ORR is presented below.

$$O_2 + 2H^+ + 2e^- \rightarrow H_2O_2 \quad (1)$$

The thermodynamic equilibrium potential (E^0) for this reaction is 0.70 V (vs. RHE), but, because H_2O_2 is absent at the start of the reaction, we observed a higher onset potential than the thermodynamic value according to the Nernst equation.

$$E = E^0 - \frac{RT}{2F}\ln(\frac{[H_2O_2]}{P_{O_2}}) \quad (2)$$

Here, the term for the proton concentration is excluded because the potential is presented with respect to the RHE, a pH-independent potential scale for proton-coupled electron transfer reactions. The redox potential becomes 0.82 and 0.79 V (vs. RHE) under 1 atm of O_2 at room temperature if we assume that $[H_2O_2]$ = 0.10 and 1.0 mM, respectively.

Supplementary figures

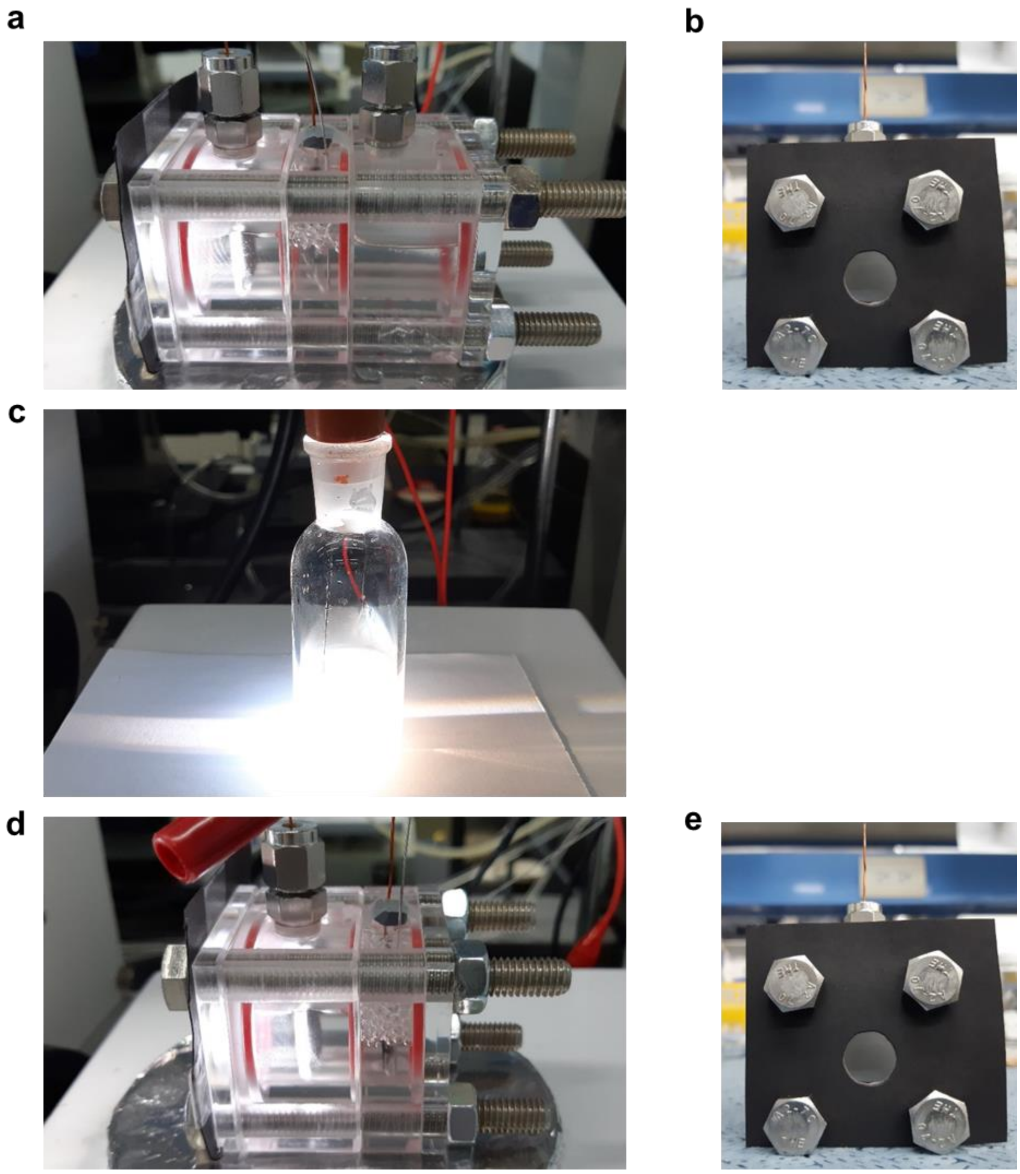

Supplementary Fig. 1 | Photographs of the reactors used in lignin valorisation experiments. a,**b**, Side and front, respectively, of the three-compartment reactor for electrode-based photo-electro-biochemical/photo-electrochemical studies. **c**, Single-compartment reactor for powder-based bio-photochemical/ photochemical studies. **d**,**e**, Side and front, respectively, of the two-compartment reactor for electrode-based photo-electro-biochemical/photo-electrochemical studies.

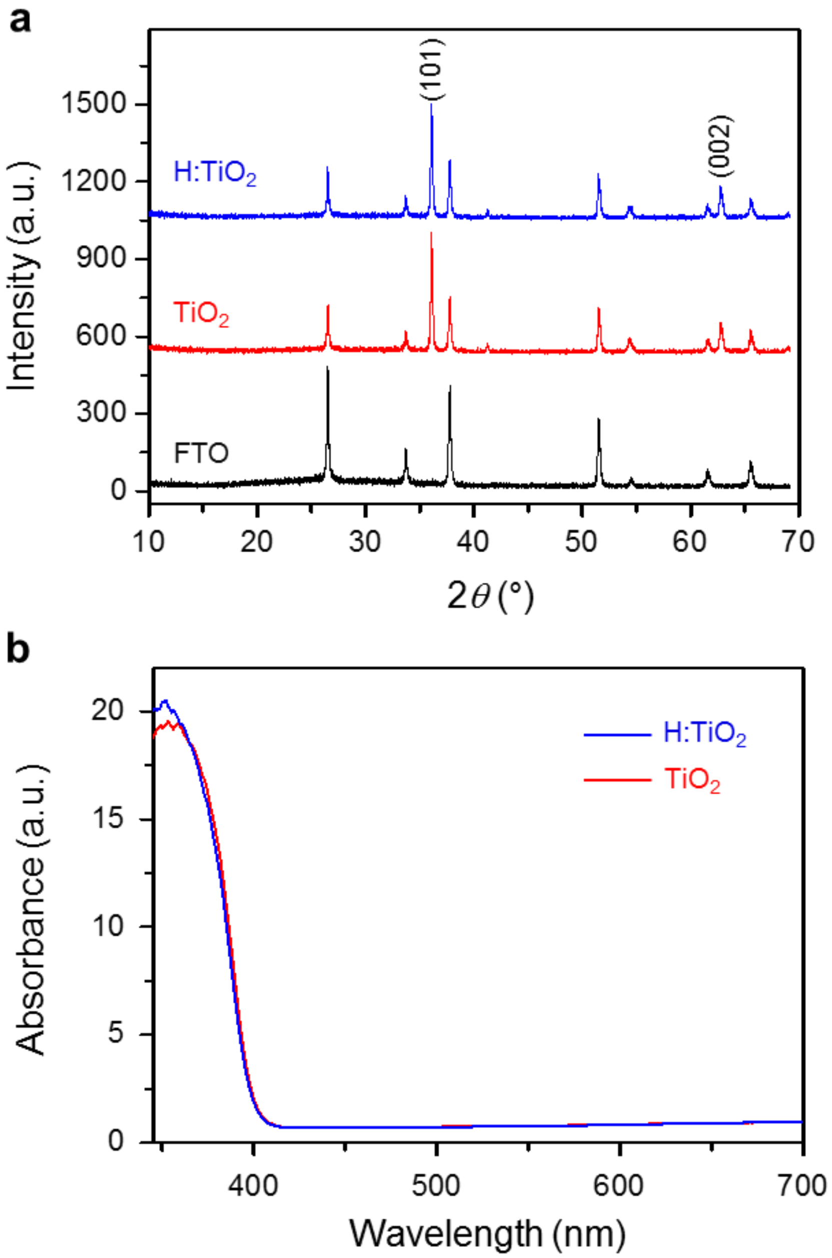

Supplementary Fig. 2 | XRD patterns and UV-vis spectra of H:TiO_2 and TiO_2. a, XRD patterns of H:TiO_2/FTO (blue line), TiO_2/FTO (red line) and FTO (black line) **b**, UV-vis diffusion reflectance spectra (DRS) of H:TiO_2/FTO (blue line) and TiO_2/FTO (red line).

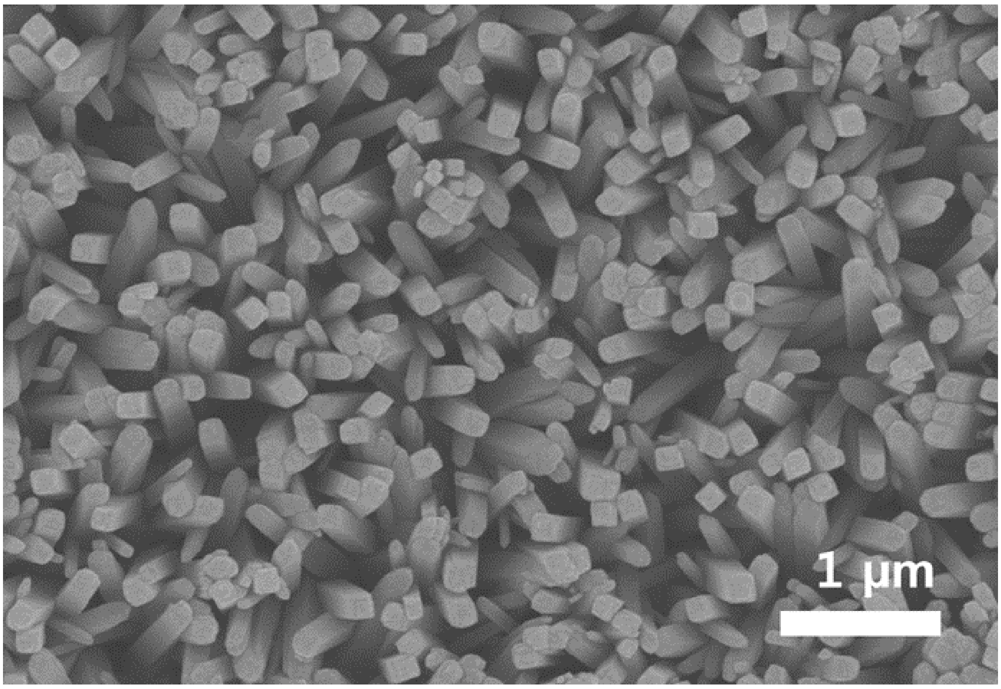

Supplementary Fig. 3 | SEM image of hydrogen-treated TiO_2/FTO photoanode (H:TiO_2).

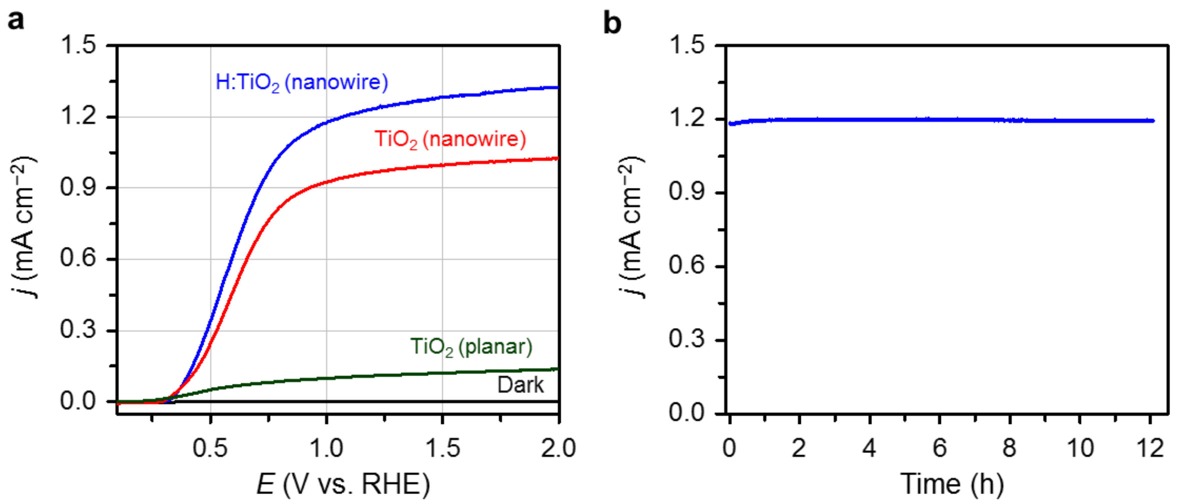

Supplementary Fig. 4 | Photoelectrochemical measurement of photoanodes. a, *I-V* curves hydrogen-treated $H:TiO_2$/FTO nanowire photoanode (blue line), TiO_2/FTO nanowire photoanode (red line) and TiO_2/FTO planar photoanode (green line) under simulated 1 sun (AM1.5G) illumination and dark (black line). **b**, Stability of the $H:TiO_2$ photoanode measured at a constant applied voltage of 1.23 (vs. RHE) in 0.1 M phosphate borate solution, pH 4.5 under simulated 1 sun (AM1.5G) illumination.

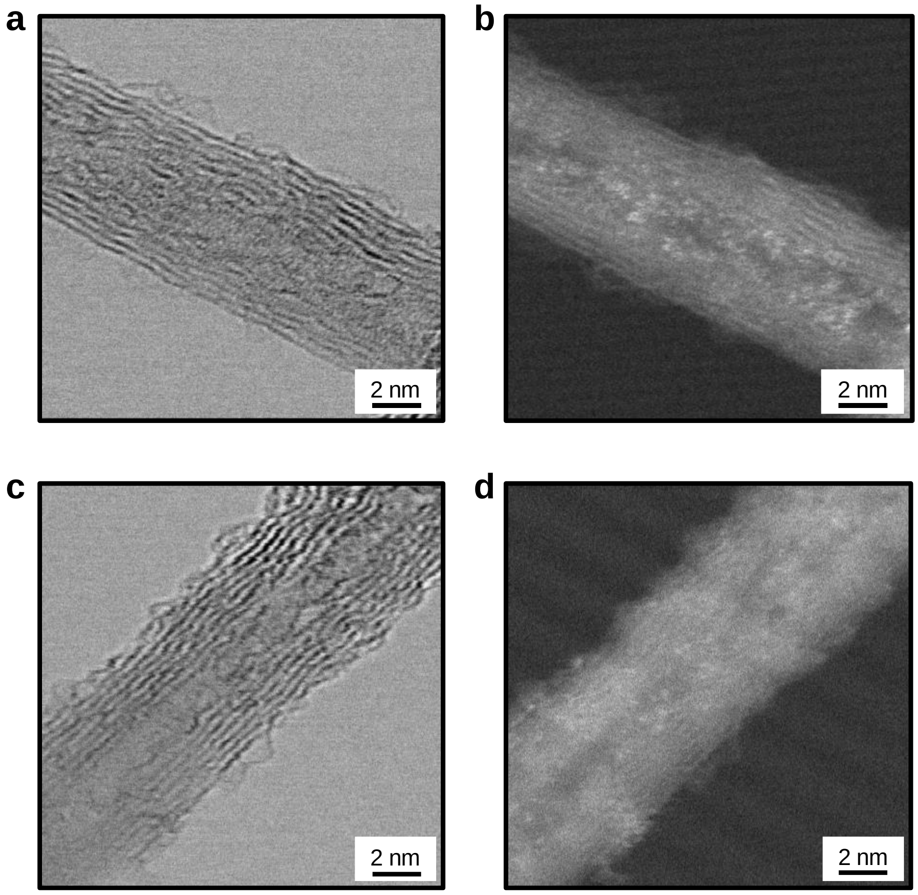

Supplementary Fig. 5 | Scanning transmission electron microscopy images of Co–N/CNT. Co–N/CNT structure **a**,**b**, before and **c**,**d**, after the potential cycling tests.

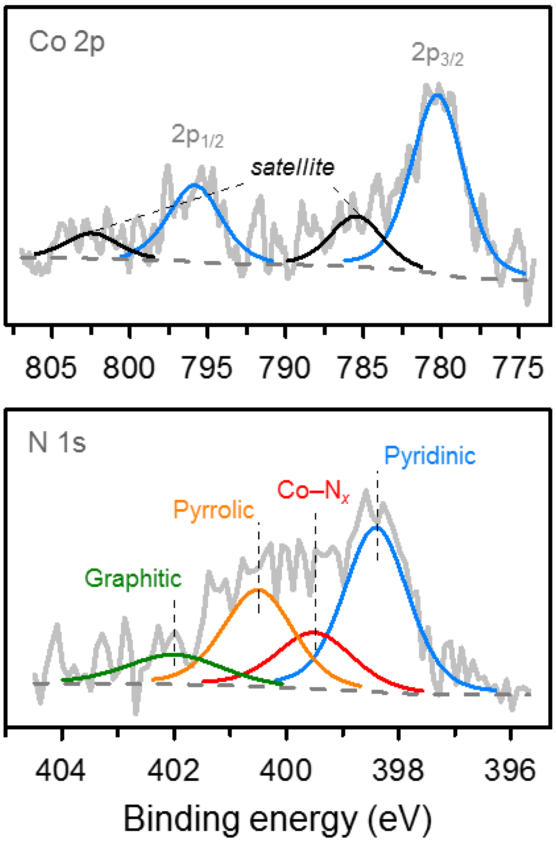

Supplementary Fig. 6 | Co 2p and N 1s XPS spectra and deconvoluted peaks of Co–N/CNT.

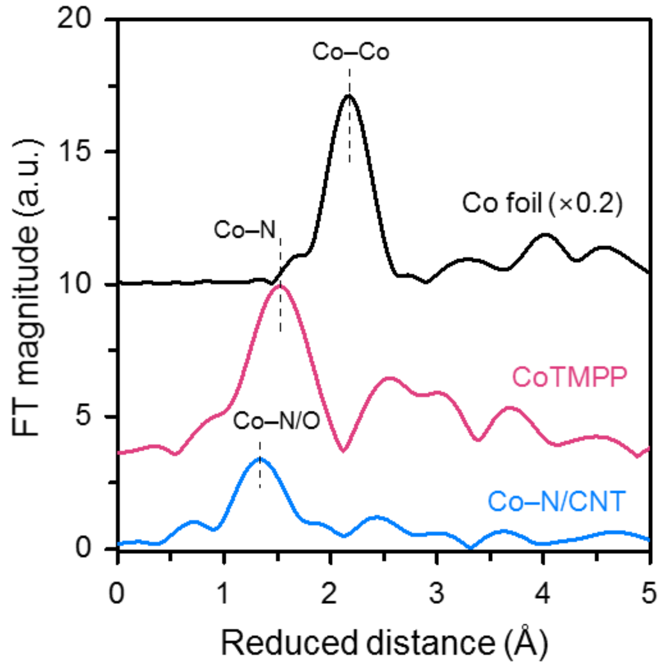

Supplementary Fig. 7 | The EXAFS spectra of Co–N/CNT, CoTMPP, and Co foil.

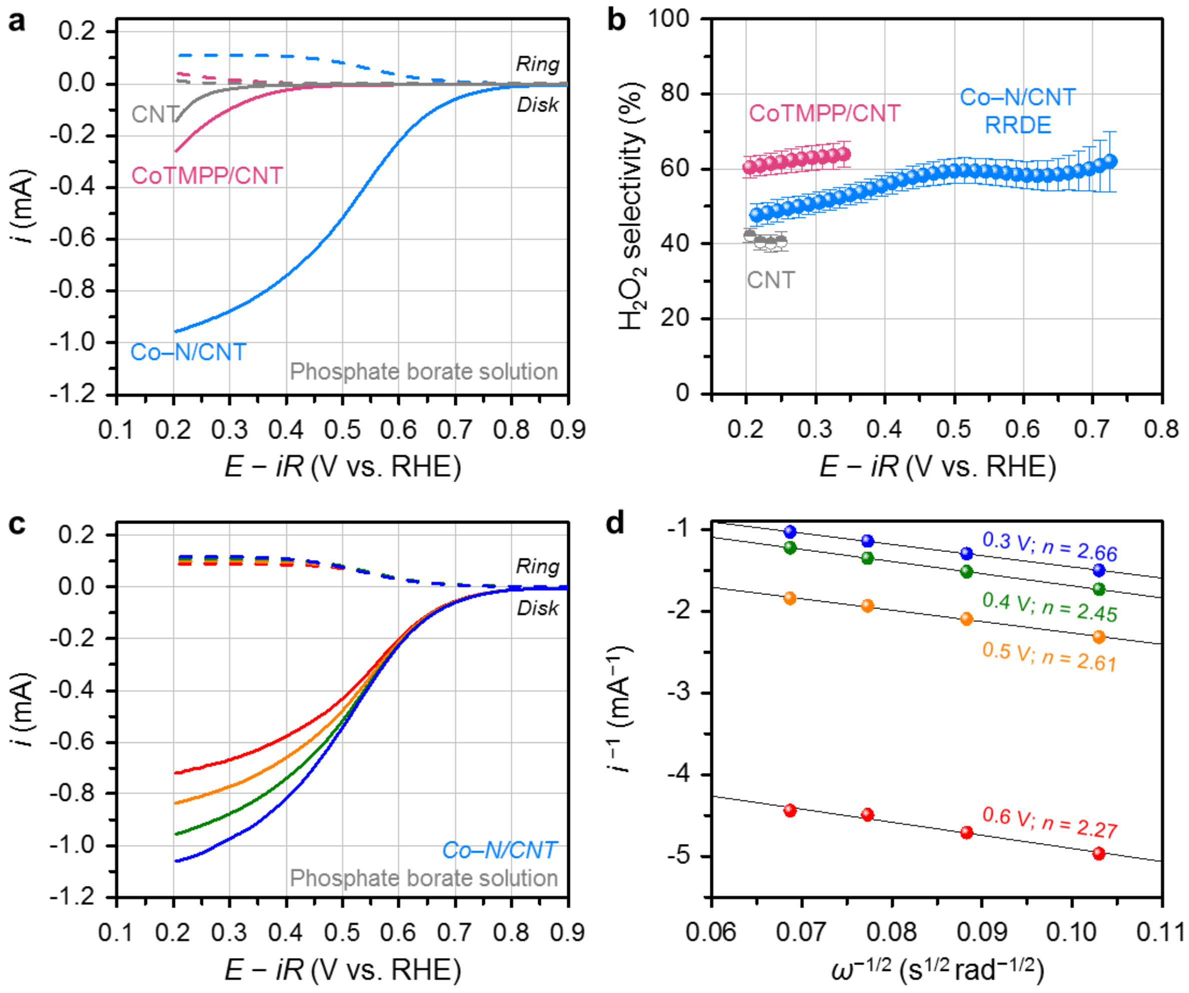

Supplementary Fig. 8 | ORR Activity and Selectivity. a, ORR polarization curves and **b**, H_2O_2 selectivity of Co–N/CNT, CoTMPP, and CNT measured in 0.1 M phosphate borate solution by the RRDE method. **c**, ORR polarization curves of Co–N/CNT measured in 0.1 M phosphate borate solution, pH 4.5, at different electrode rotation speeds and **d**, corresponding K–L plots.

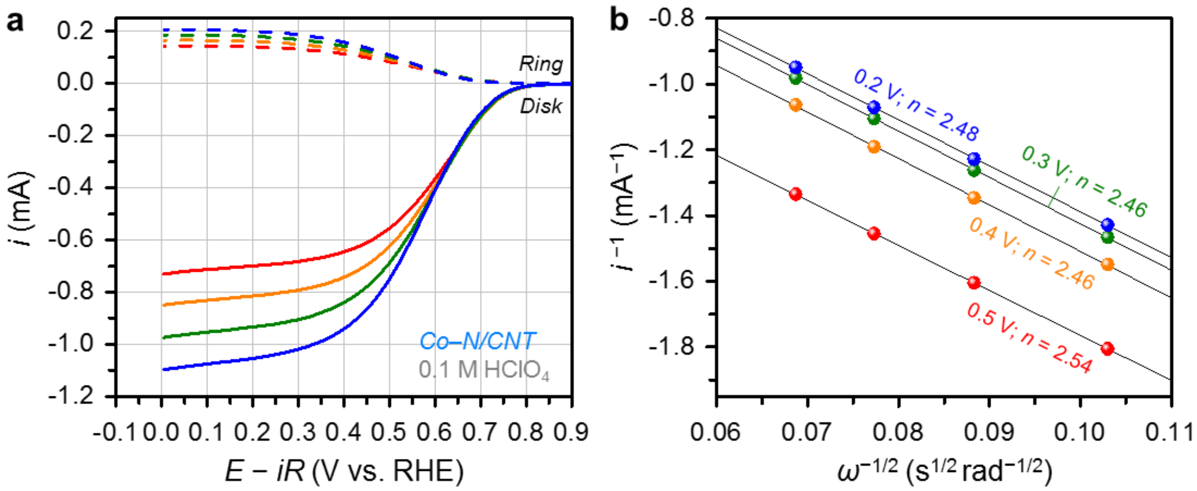

Supplementary Fig. 9 | K–L Analysis of Co–N/CNT in 0.1 M $HClO_4$. a, ORR polarisation curves of Co–N/CNT measured in 0.1 M $HClO_4$ at different electrode rotation speeds and **b**, the corresponding K–L plots.

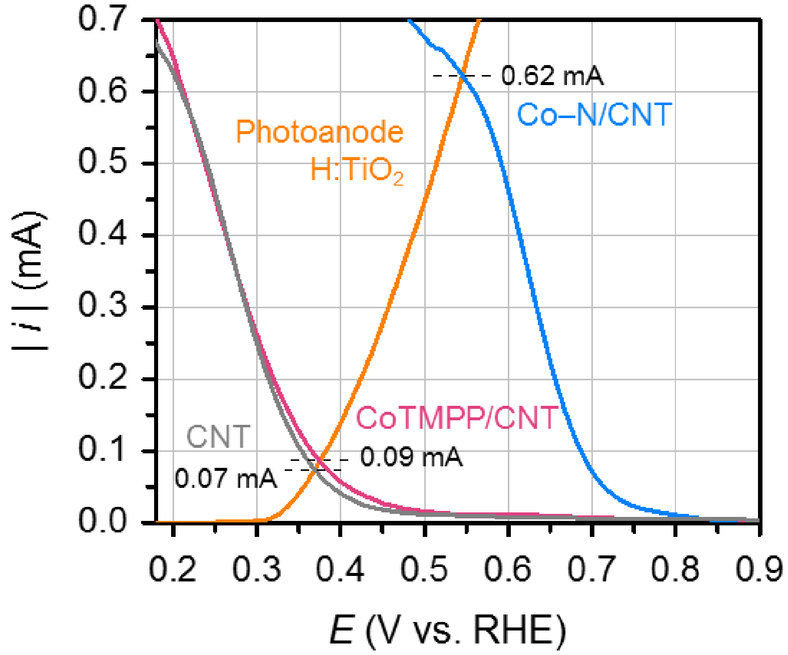

Supplementary Fig. 10 | *I-V* curve of H:TiO$_2$ photoanode and Co–N/CNT, CoTMPP/CNT, and CNT cathodes. H:TiO$_2$ photoanode under simulated 1 sun (AM1.5G) illumination in 0.1 M phosphate borate solution at pH 4.5 and *I-V* curves of Co–N/CNT, CoTMPP/CNT, and CNT cathodes in O$_2$-saturated 0.1 M phosphate borate solution at pH 4.5.

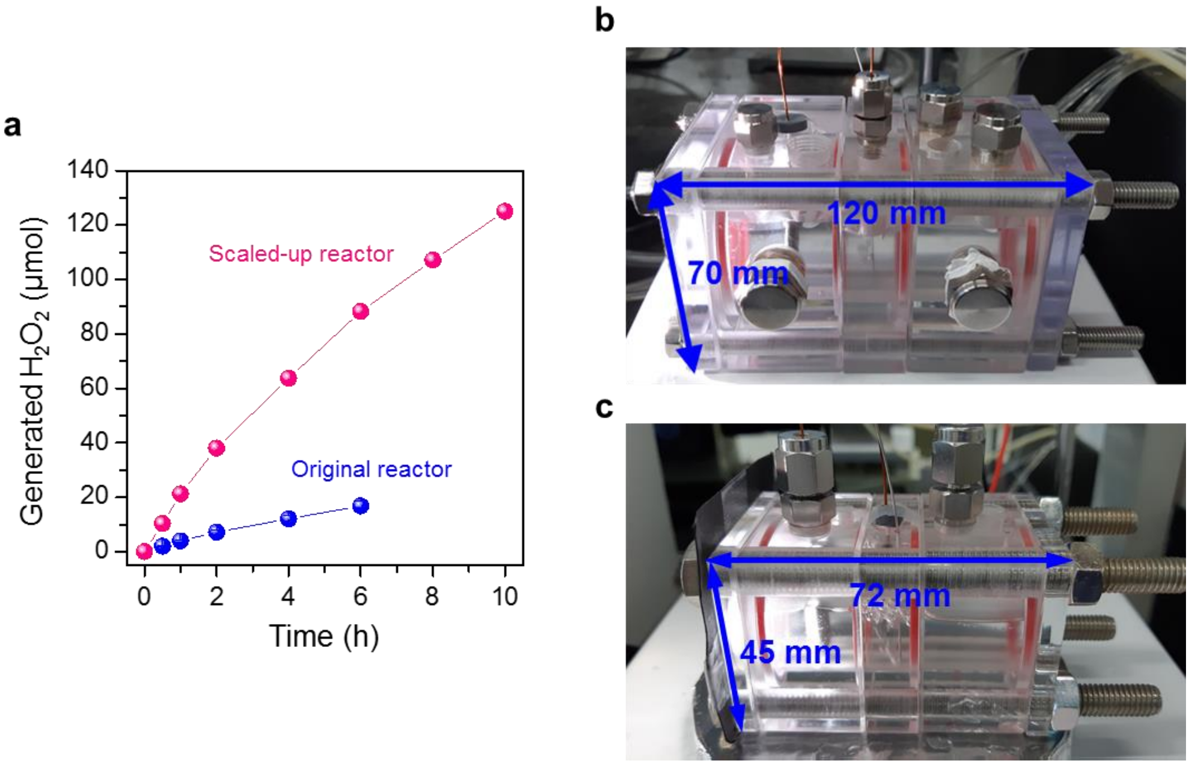

Supplementary Fig. 11 | Photo-electrocatalytic H_2O_2 production in the 5 times scaled-up reactor. a, Amount of H_2O_2 generated over time in 0.1 M phosphate borate solution, pH 4.5 under 1 sun (AM1.5G) illumination in different-sized reactors. **b**,**c**, Digital pictures of the scaled-up and original three-compartment reactors. Electrolyte volume: anode, cathode, and enzyme cells contained 40, 20, and 40 ml of electrolyte for the scaled-up reactor, respectively, while the original reactor contained 5 times lower amounts of electrolyte in each cell. Electrode areas: H:TiO_2 photoanode illuminated area was 8.0 cm^2 and Co–N/CNT cathode area was 10 cm^2 for the scaled-up reactor, while the original reactor had 6 and 5 times smaller photoanode and cathode, respectively. Light source: 300 W Xenon lamp (Newport, 66902) with AM1.5G filter for the scaled-up reactor, while the solar simulator (Abet Technologies, 10500) was used for the original reactor and 100 mW cm^{-2} (AM1.5G) of light intensity was used for both cases.

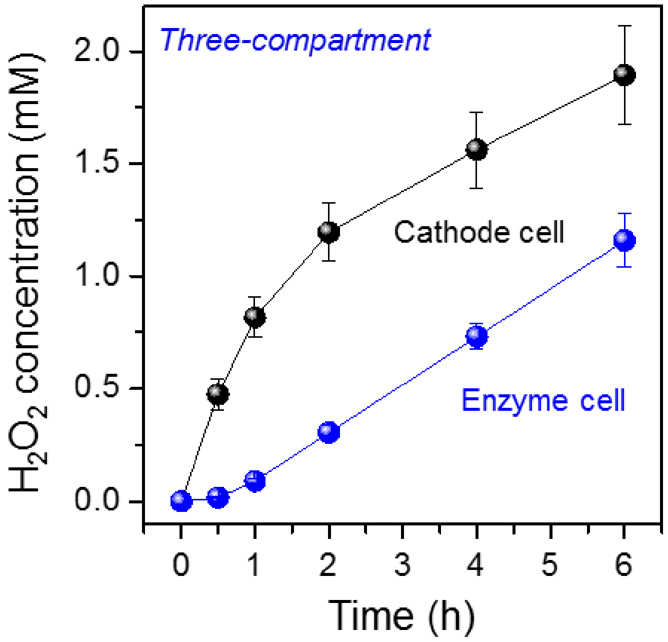

Supplementary Fig. 12 | Photo-electrochemical H_2O_2 production in the three-compartment cell. Changes in H_2O_2 concentration as a function of time in the cathode cell (4 mL) and enzyme cell (8 mL) under illumination. Experimental conditions: 0.1 M phosphate borate solution, pH 4.5, and solar simulator irradiation at 100 mW cm^{-2} (AM1.5G). Error bars indicate the standard deviation.

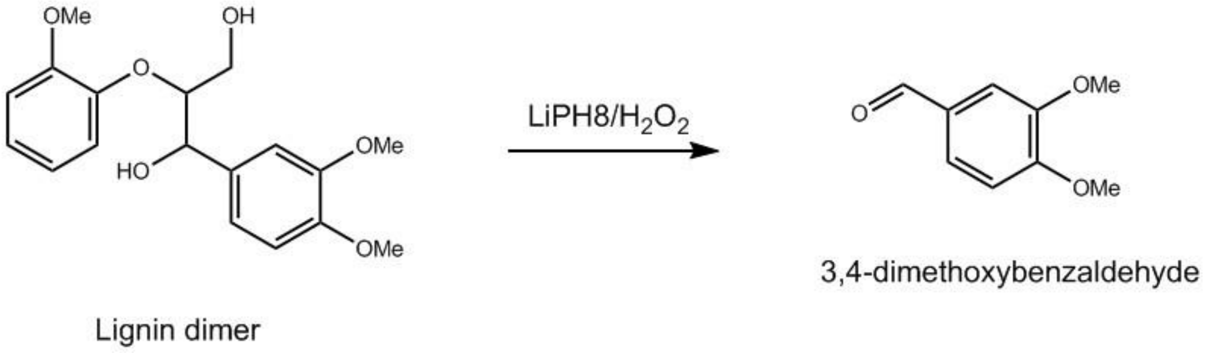

Supplementary Fig. 13 | Reaction scheme showing photo-electro-biochemical conversion of the lignin dimer to valuable product

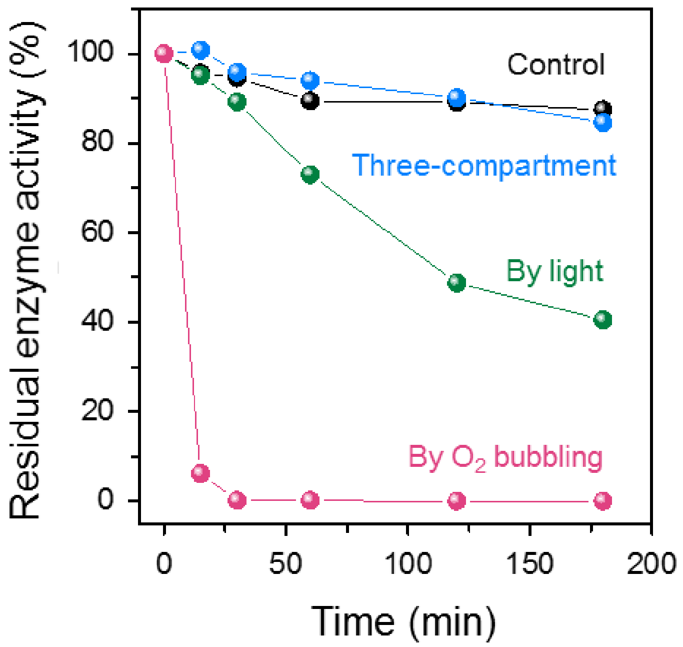

Supplementary Fig. 14 | Enzyme LiPH8 stability test. Effect of O_2 purging and exposure to light irradiation on the activity of LiPH8. Residual enzyme activity after incubation with stirring in phosphate borate solution, pH 4.5: without light irradiation and O_2 purging (black circles), with light irradiation (green circles), with O_2 purging (pink circles) in the two-compartment reactor. The blue circles represent the variation in enzyme activity with light irradiation and O_2 purging in three-compartment reactor. All experiments were performed with constant stirring at room temperature.

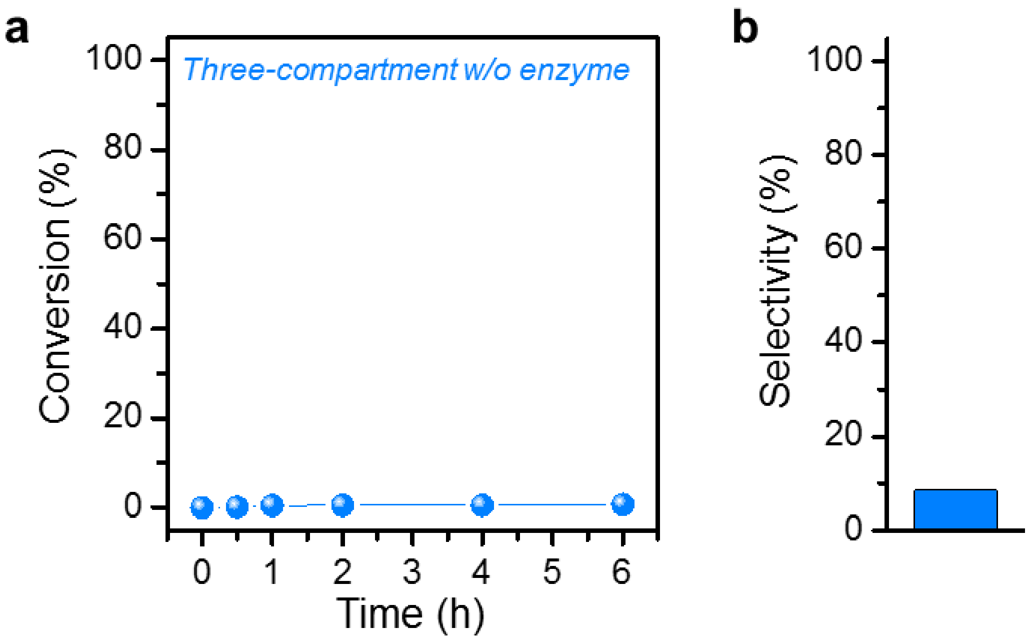

Supplementary Fig. 15 | Photo-electrochemical lignin degradation (i.e. without enzyme) in the three-compartment reactor. a, Lignin conversion and **b**, selectivity. Experimental conditions: 0.1 M phosphate borate solution, pH 4.5, and solar simulator irradiation at 100 mW cm^{-2} (AM1.5G).

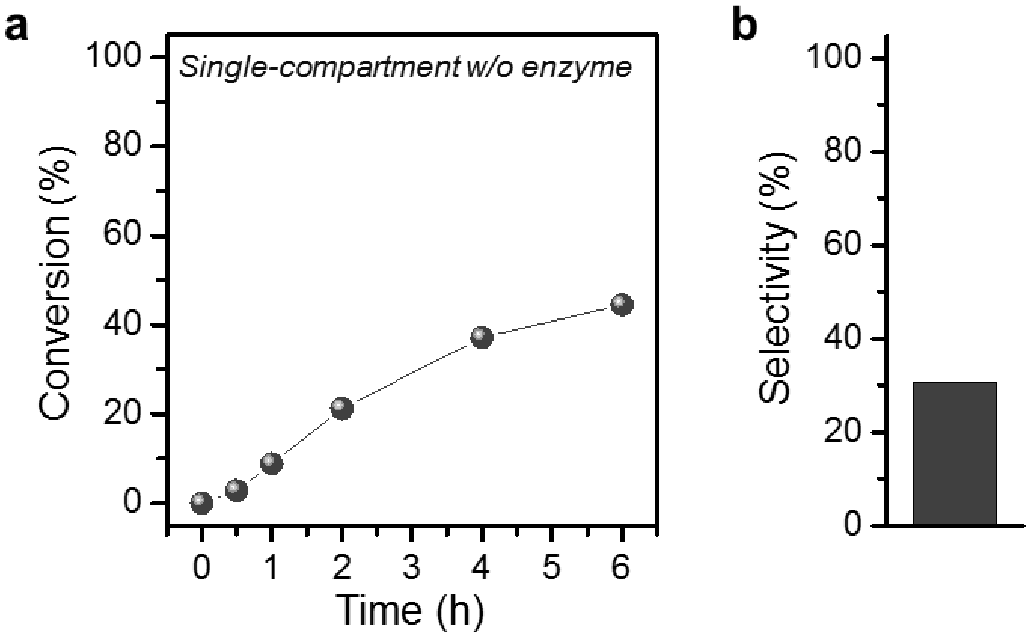

Supplementary Fig. 16 | Photochemical lignin degradation (i.e. without enzyme) in the single-compartment reactor. a, Lignin conversion and **b**, selectivity. Experimental conditions: 0.1 M phosphate borate solution, pH 4.5, reaction volume 8 mL, catalyst concentration 0.5 mg mL^{-1}, and solar simulator irradiation at 100 mW cm^{-2} (AM1.5G).

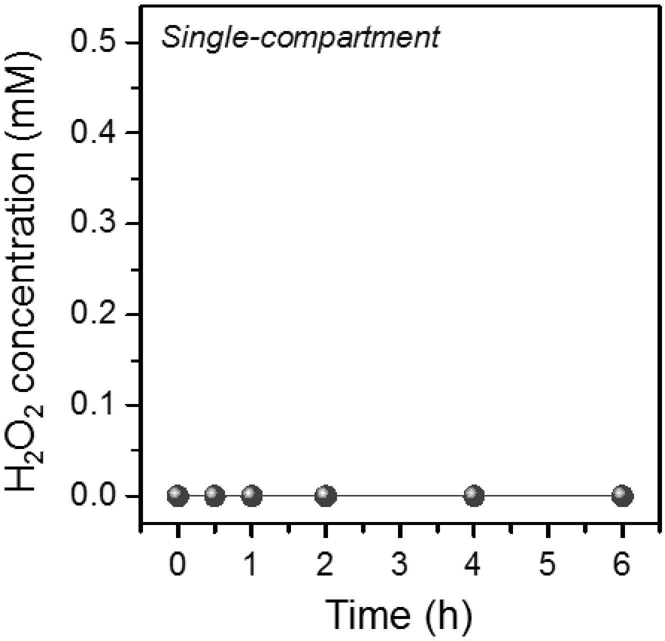

Supplementary Fig. 17 | Photochemical H_2O_2 production in the single-compartment reactor. Changes in H_2O_2 concentration as a function of time under illumination. Experimental conditions: 0.1 M phosphate borate solution, pH 4.5, reaction volume 8 mL, catalyst concentration 0.5 mg mL^{-1}, and solar simulator irradiation at 100 mW cm^{-2} (AM1.5G).

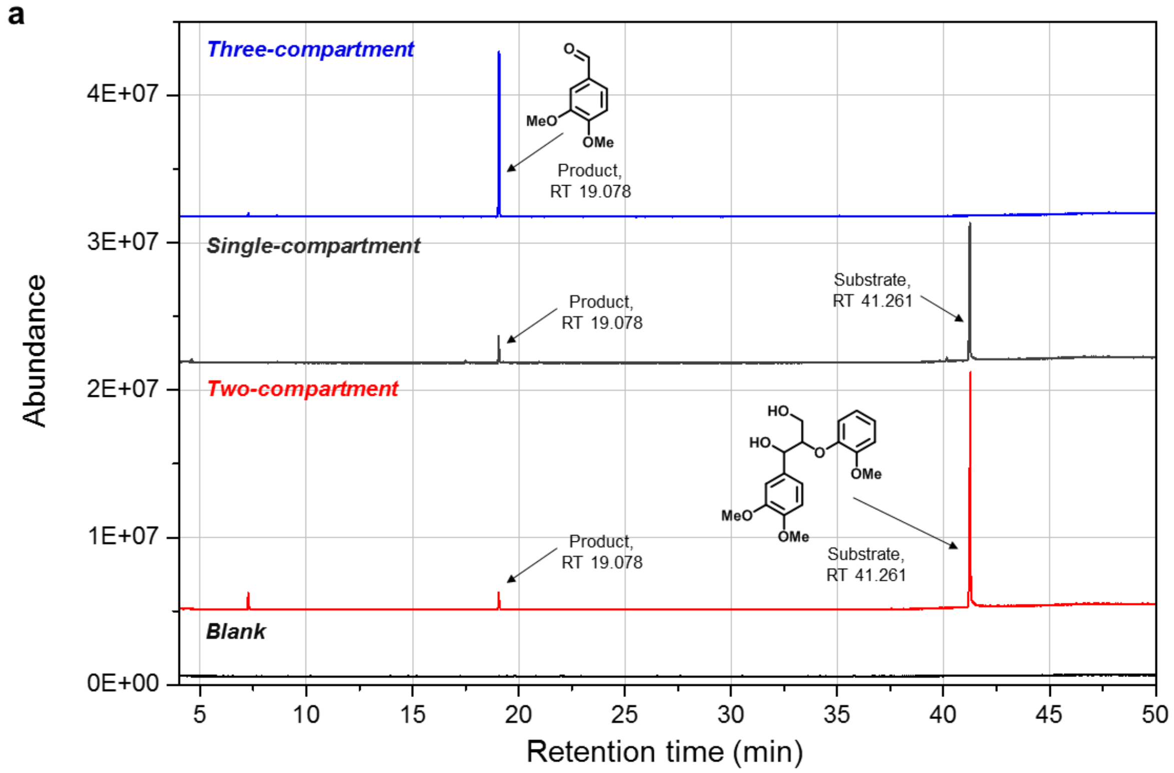

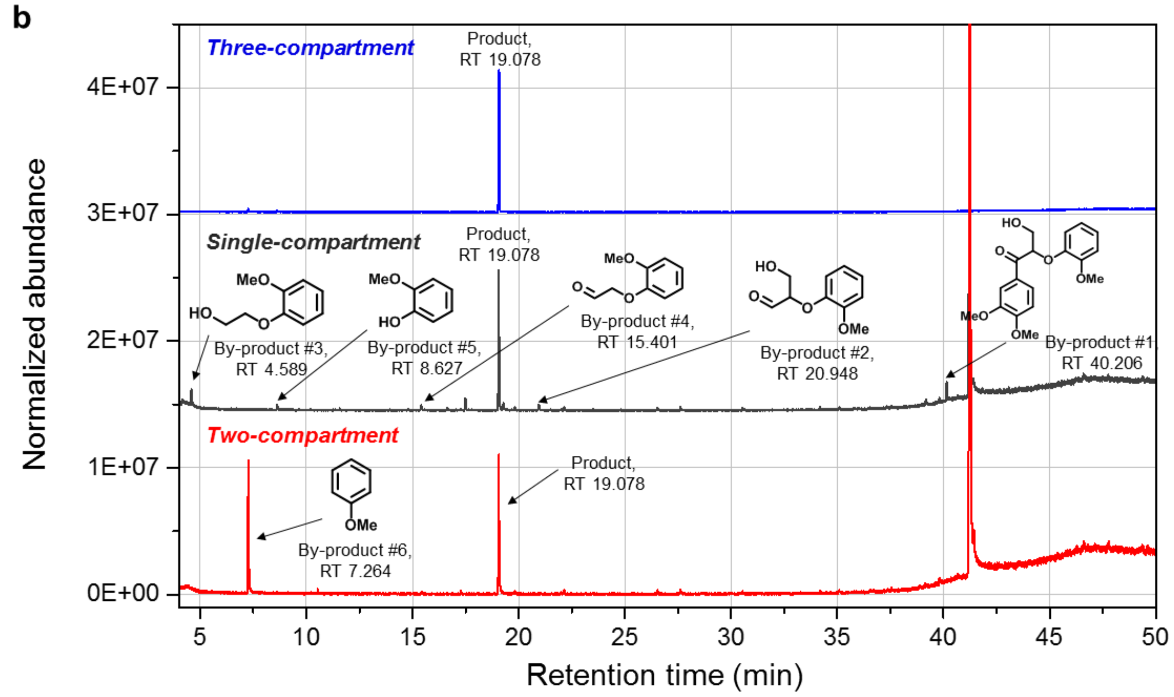

Supplementary Fig. 18 | GC-MS analysis of products from photo-electro-biochemical lignin degradation. a, GC-MS chromatograms of soluble fractions from reaction media of the three, single, and two-compartment systems. **b,** Normalised data of a (the product peak is normalised as the same height).

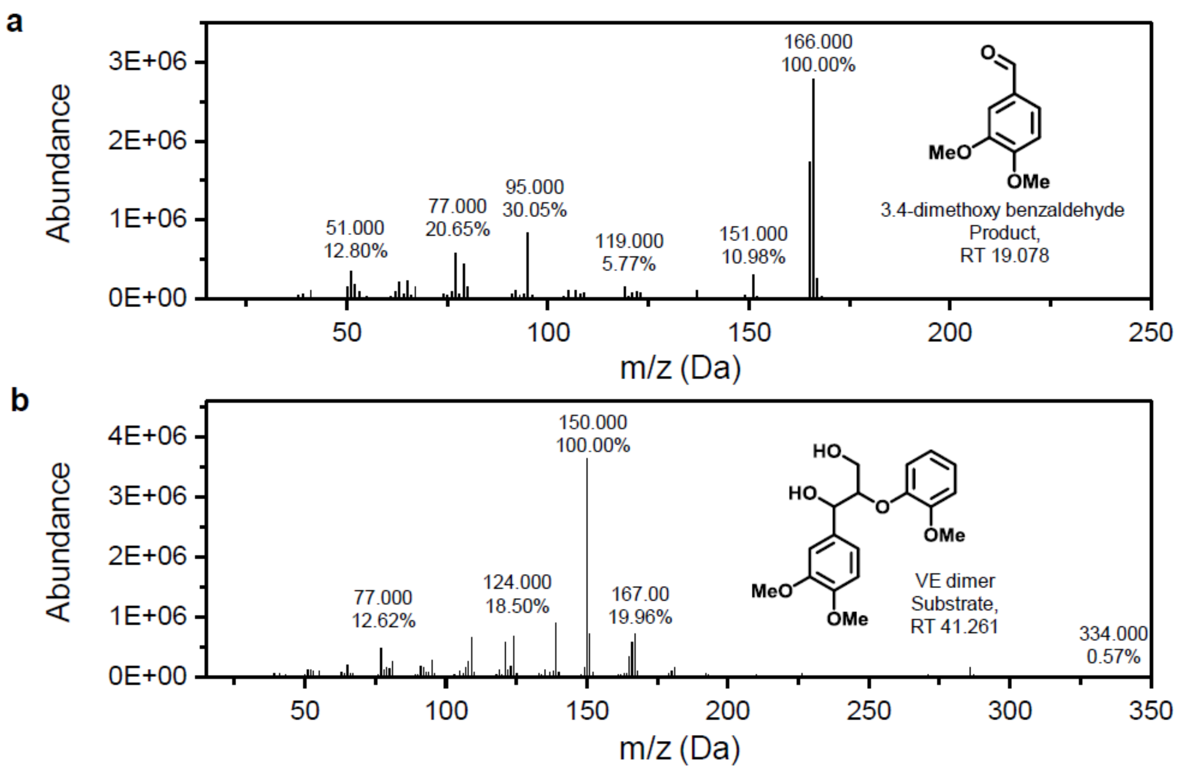

Supplementary Fig. 19 | Mass spectrum of substrate and products identified during photo-electro-biochemical lignin degradation. a, Products **b**, Substrate.

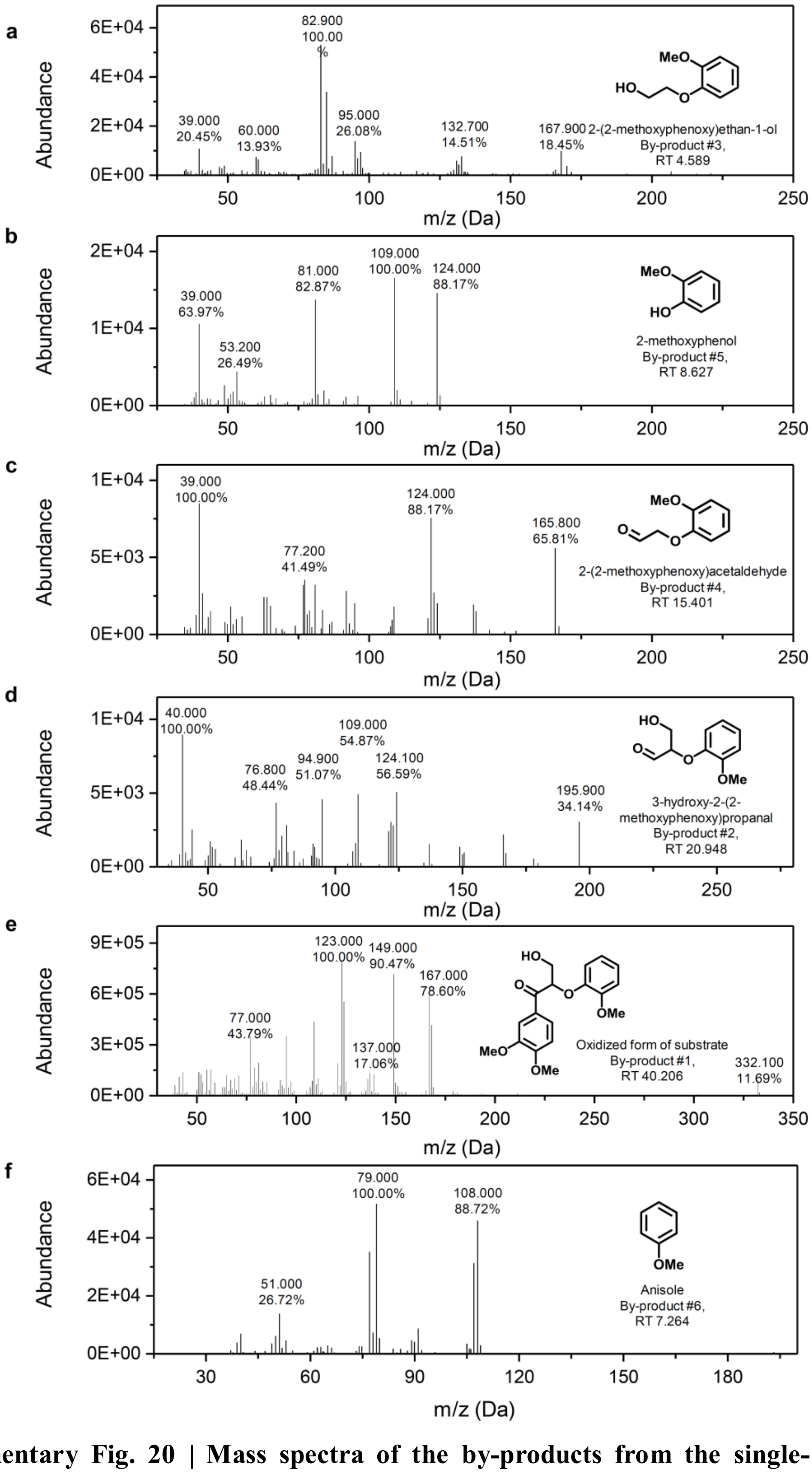

Supplementary Fig. 20 | Mass spectra of the by-products from the single- and two-compartment reactor during lignin degradation. a, By-product #3. **b**, By-product #5. **c**, By-product #4. **d**, By-product #2. **e**, By-product #1, **f**, By-product #6.

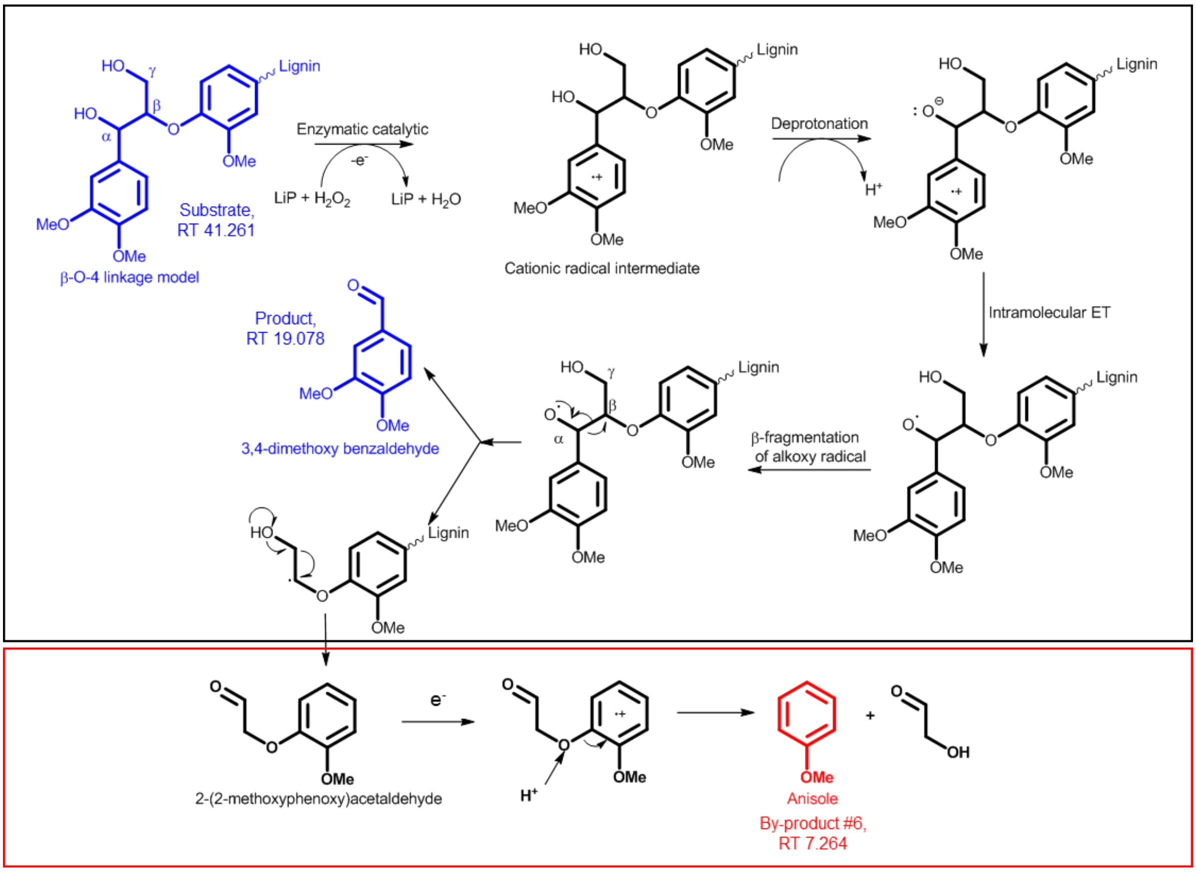

Supplementary Fig. 21 | A proposed mechanism for breaking of the β-O-4 linkage. Reaction mechanism of lignin dimer by the LiPH8 enzyme and H_2O_2 in the three-compartment reactor (in black rectangular), and the proposed side reaction in the two-compartment reactor (in red rectangular).

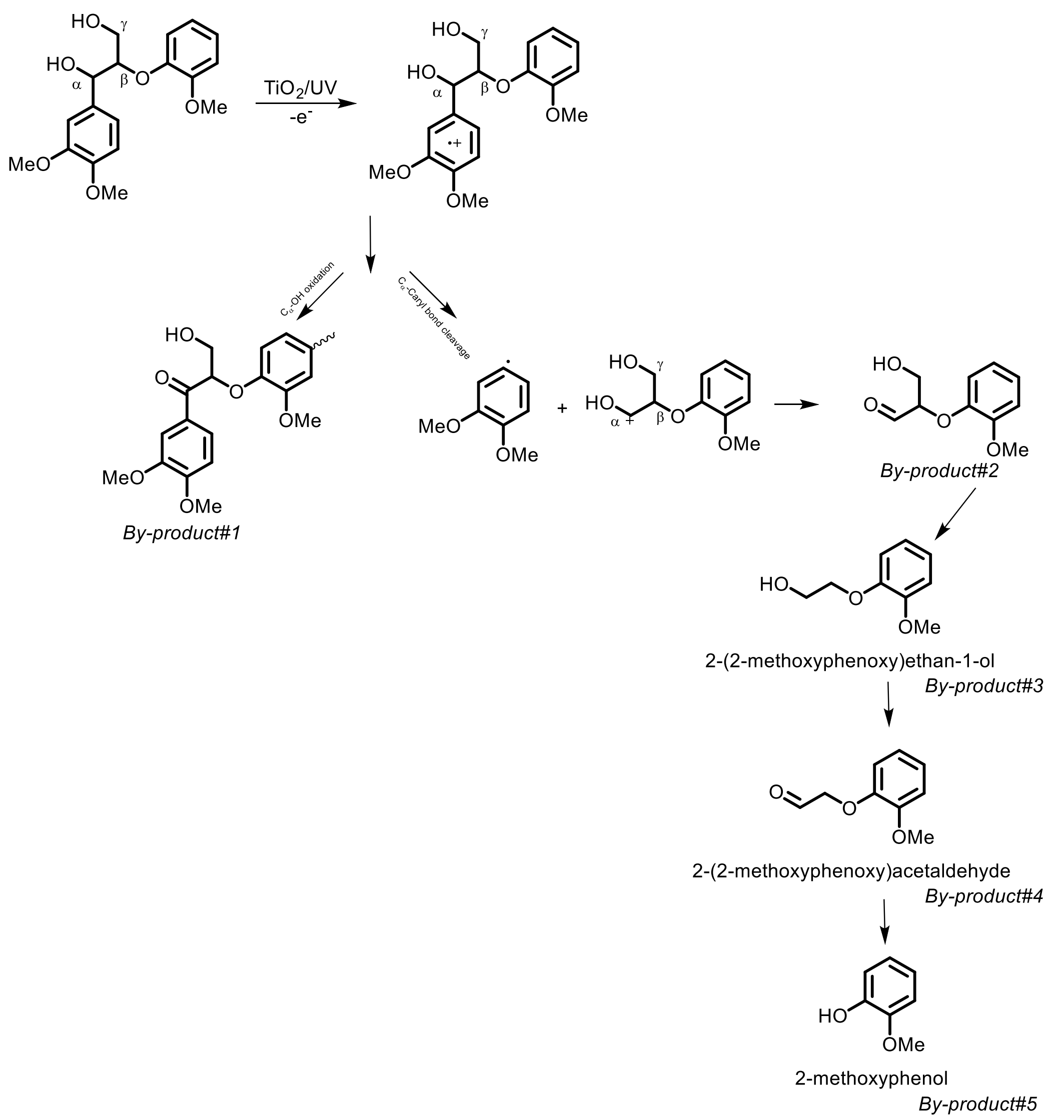

Supplementary Fig. 22 | Scheme for product formation under unselective catalysis of TiO_2/UV on lignin dimer in the single-compartment reactor.

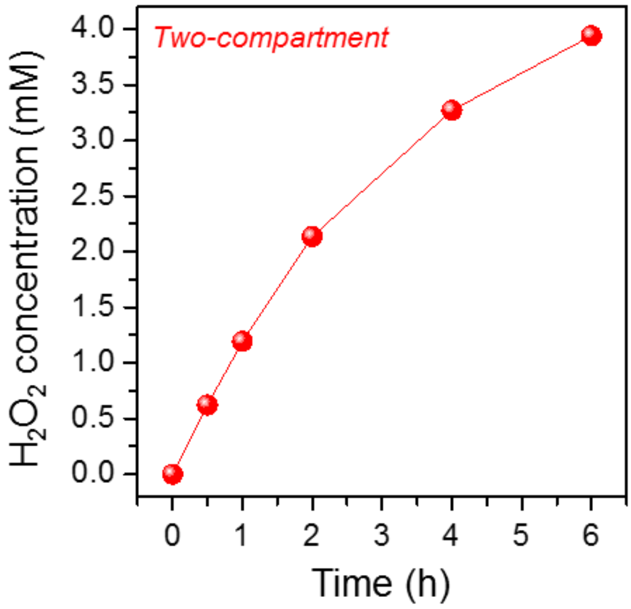

Supplementary Fig. 23 | Photo-electrochemical H_2O_2 production in the two-compartment reactor. Variation in the H_2O_2 concentration as a function of time in the cathodic compartment (4 mL) under illumination. Experimental conditions: 0.1 M phosphate borate solution, pH 4.5, and solar simulator irradiation at 100 mW cm^{-2} (AM1.5G).

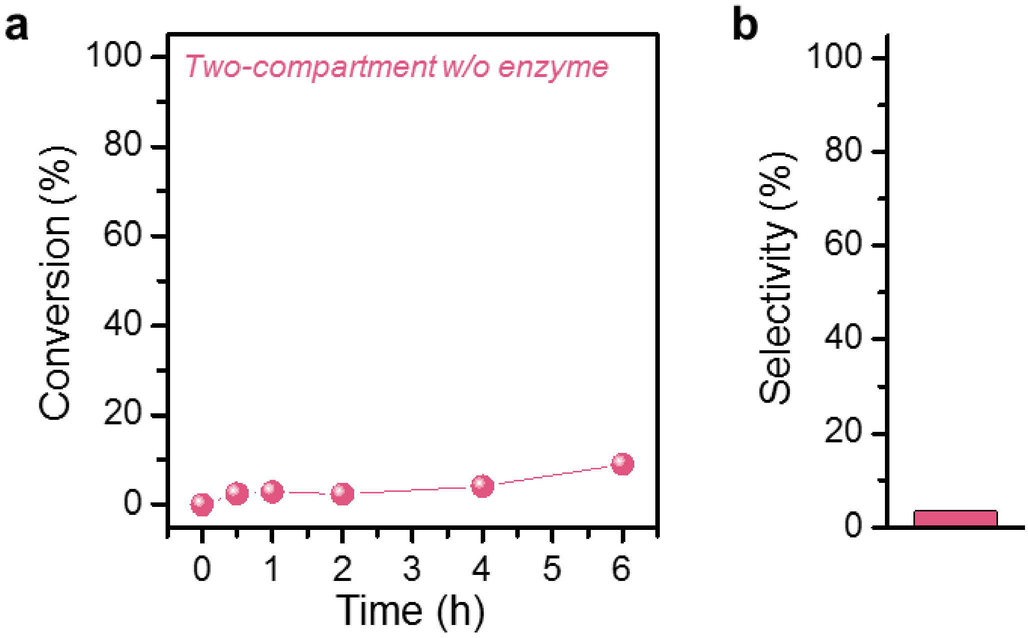

Supplementary Fig. 24 | Photo-electrochemical lignin degradation (i.e. without enzyme) in the two-compartment reactor. a, Lignin conversion and **b**, selectivity. Experimental conditions: 0.1 M phosphate borate solution, pH 4.5, and solar simulator irradiation at 100 mW cm^{-2} (AM1.5G).

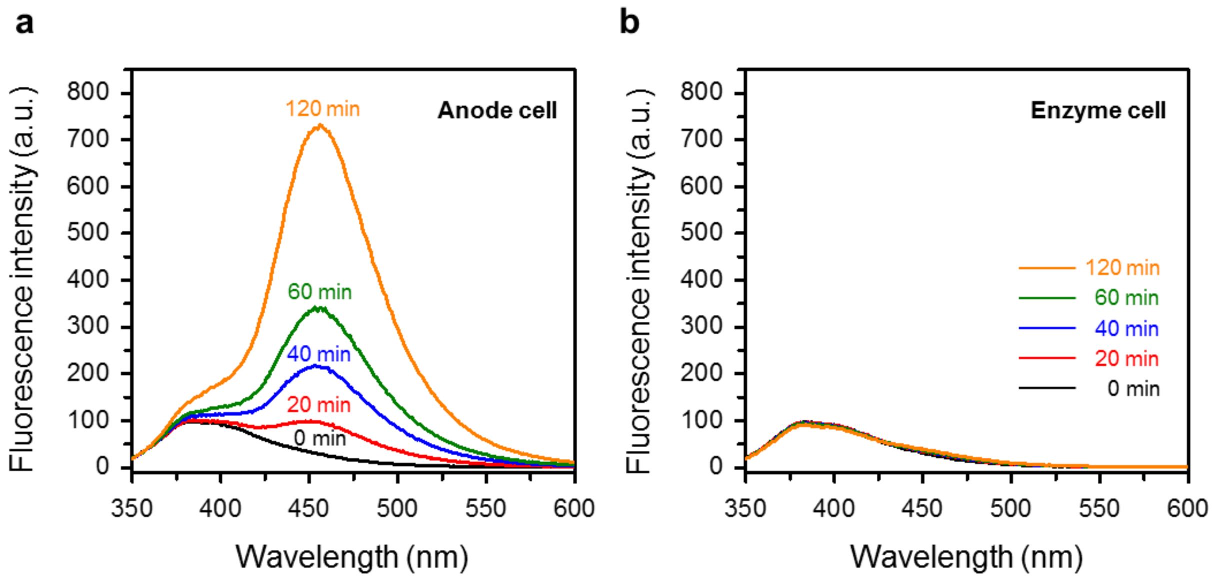

Supplementary Fig. 25 | Time-dependent fluorescence spectra of 0.2 mM coumarin solution. a, Anode cell and **b**, enzyme cell during unassisted H_2O_2 production.

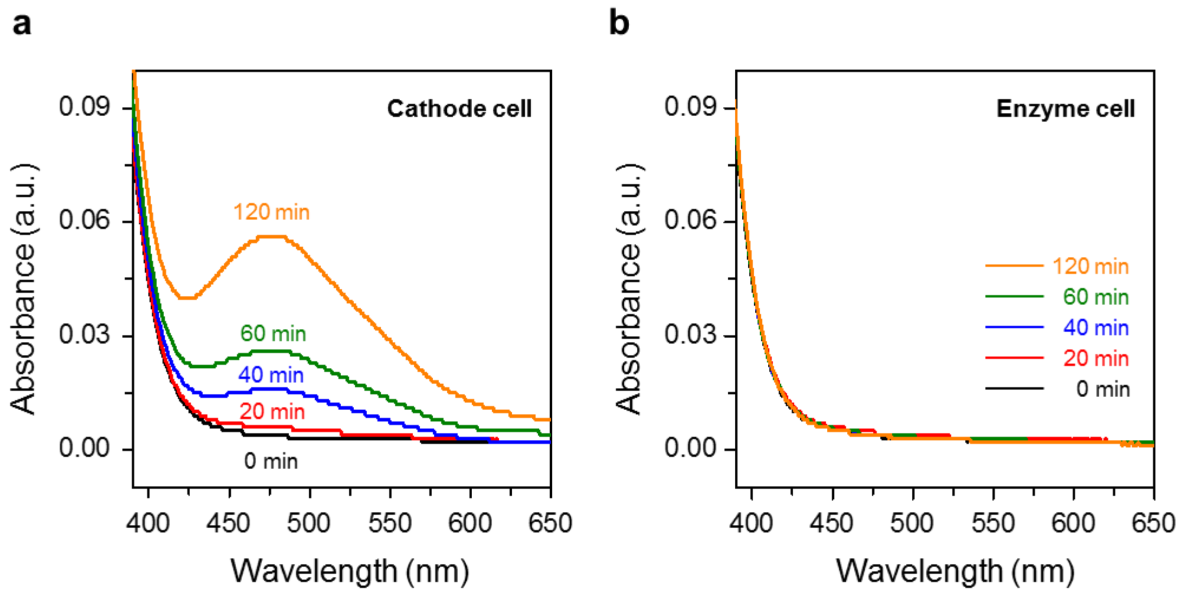

Supplementary Fig. 26 | Time-dependent UV-vis spectra of 0.1 mM XTT solution. a, Cathode cell and **b**, enzyme cell during unassisted H_2O_2 production.

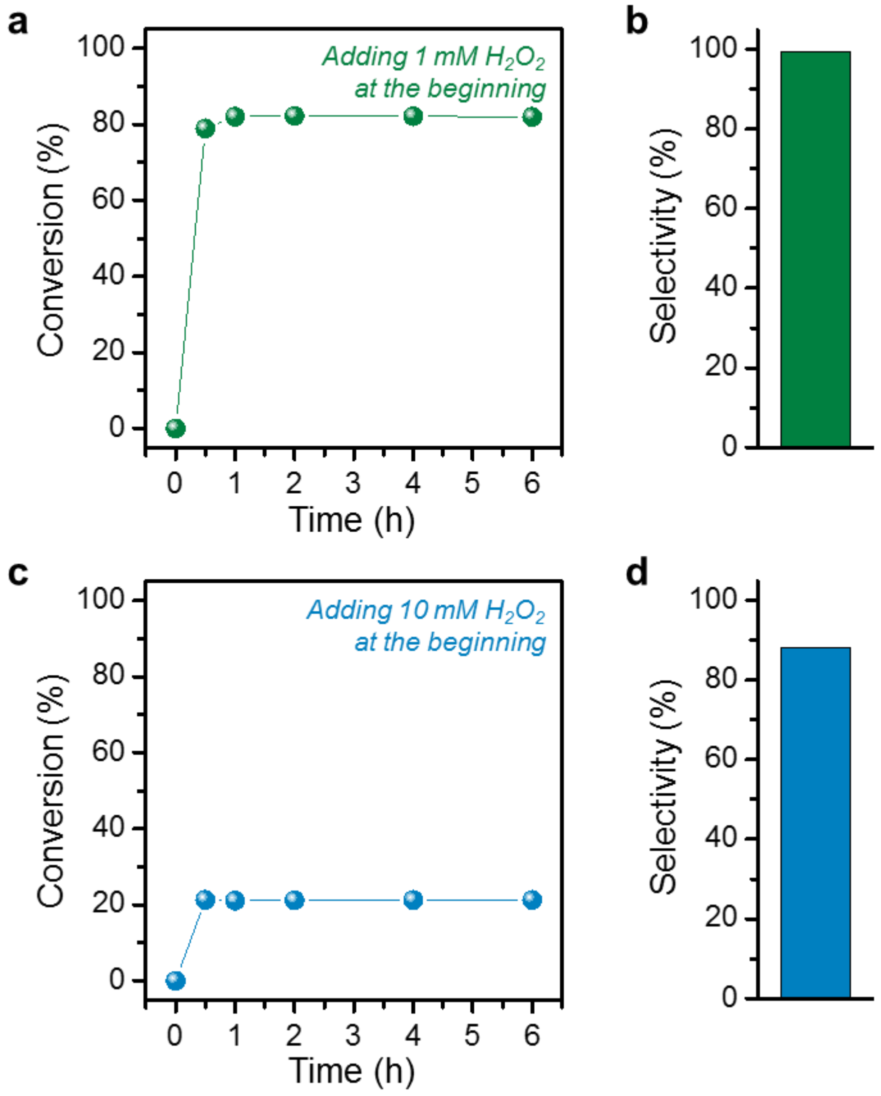

Supplementary Fig. 27 | Biocatalytic lignin degradation with H_2O_2 addition at the beginning. **a**,**c**, Lignin conversion and **b**,**d**, selectivity at 1 mM (**a**,**b**) and 10 mM (**c**,**d**) H_2O_2 concentration. Experimental conditions: 0.1 M phosphate borate solution, pH 4.5, 0.5 mM of lignin dimer, 0.8 µM of the LiPH8 enzyme, and adding 1 or 10 mM H_2O_2 at the beginning of the reaction.

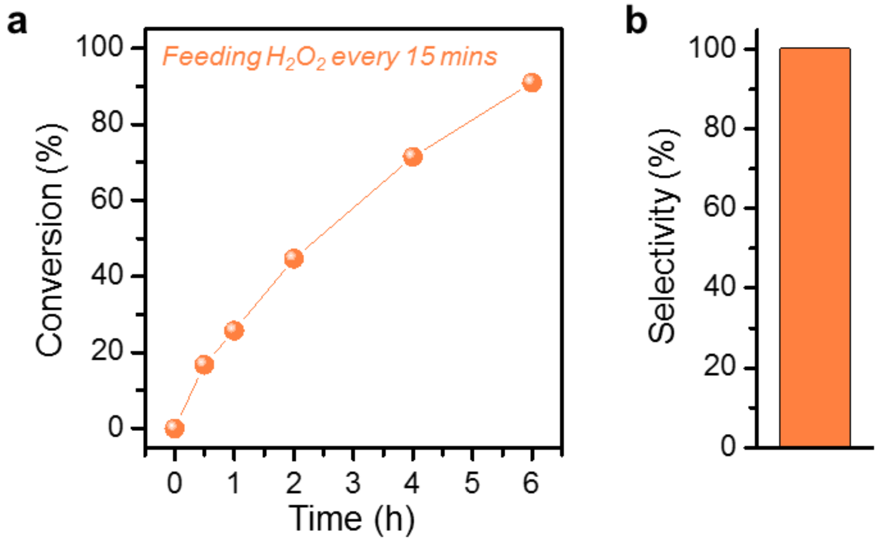

Supplementary Fig. 28 | Biocatalytic lignin degradation with continuous H_2O_2 feeding. a, Lignin conversion and **b**, selectivity. Experimental conditions: 0.1 M phosphate borate solution, pH 4.5, 0.5 mM of lignin dimer, 0.8 μM of the LiPH8 enzyme, and feeding H_2O_2 every 15 mins (total 1 mM).

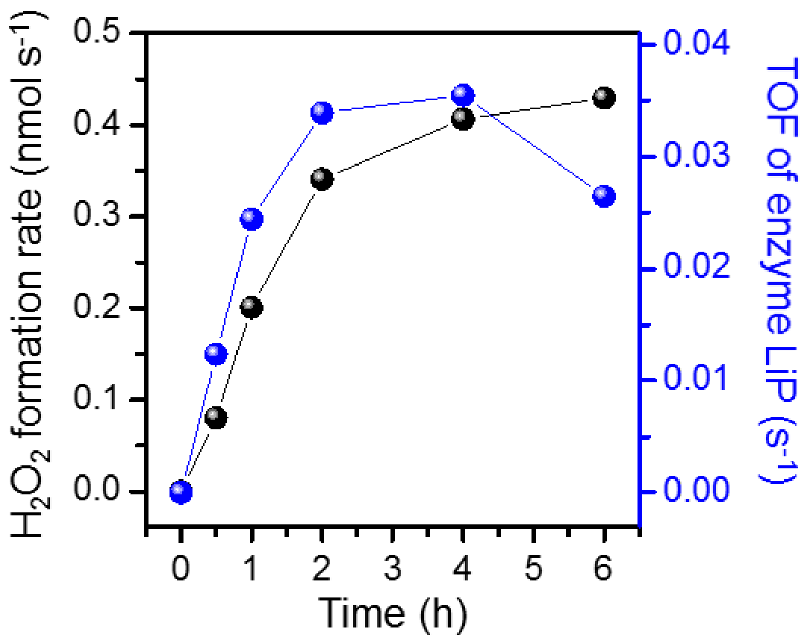

Supplementary Fig. 29 | Correlation between H_2O_2 formation rate and TOF of enzyme LiP. H_2O_2 formation rate in the enzyme cell (black circles) and turnover frequency of enzyme LiP (blue circles).

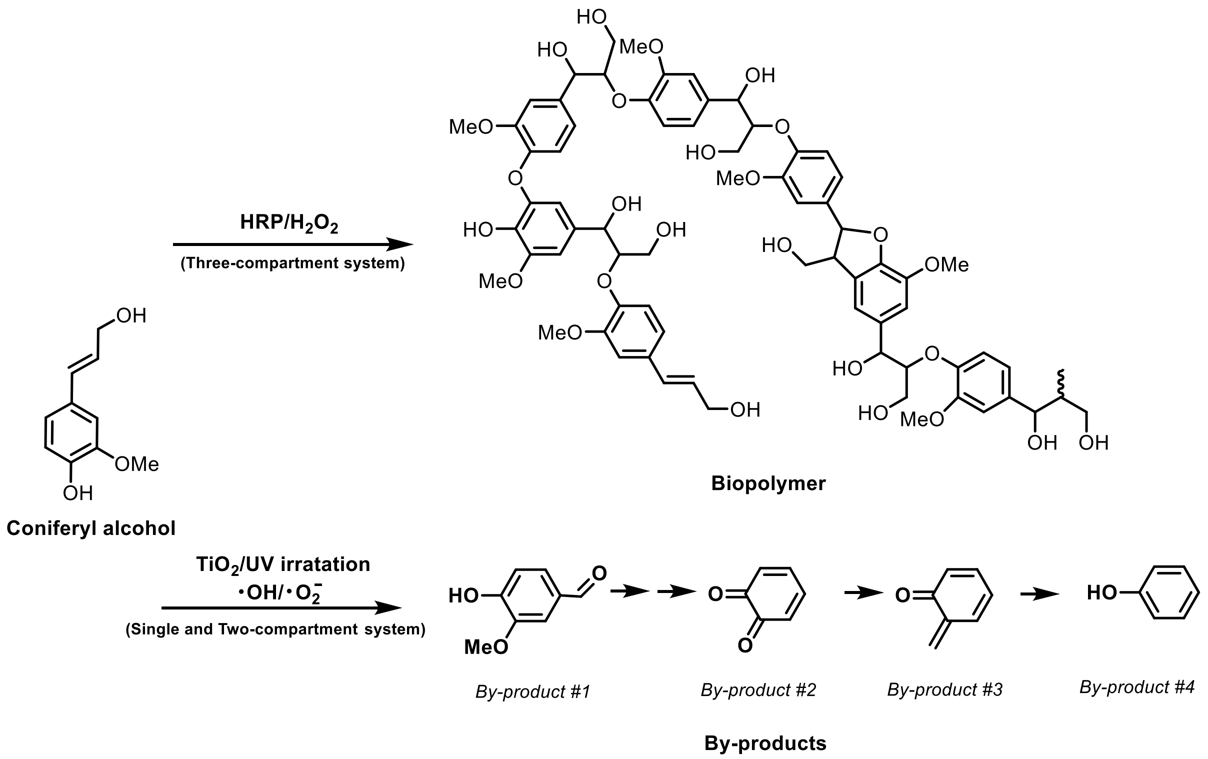

Supplementary Fig. 30 | Reaction schemes for the pathways of coniferyl alcohol in different reactor systems.

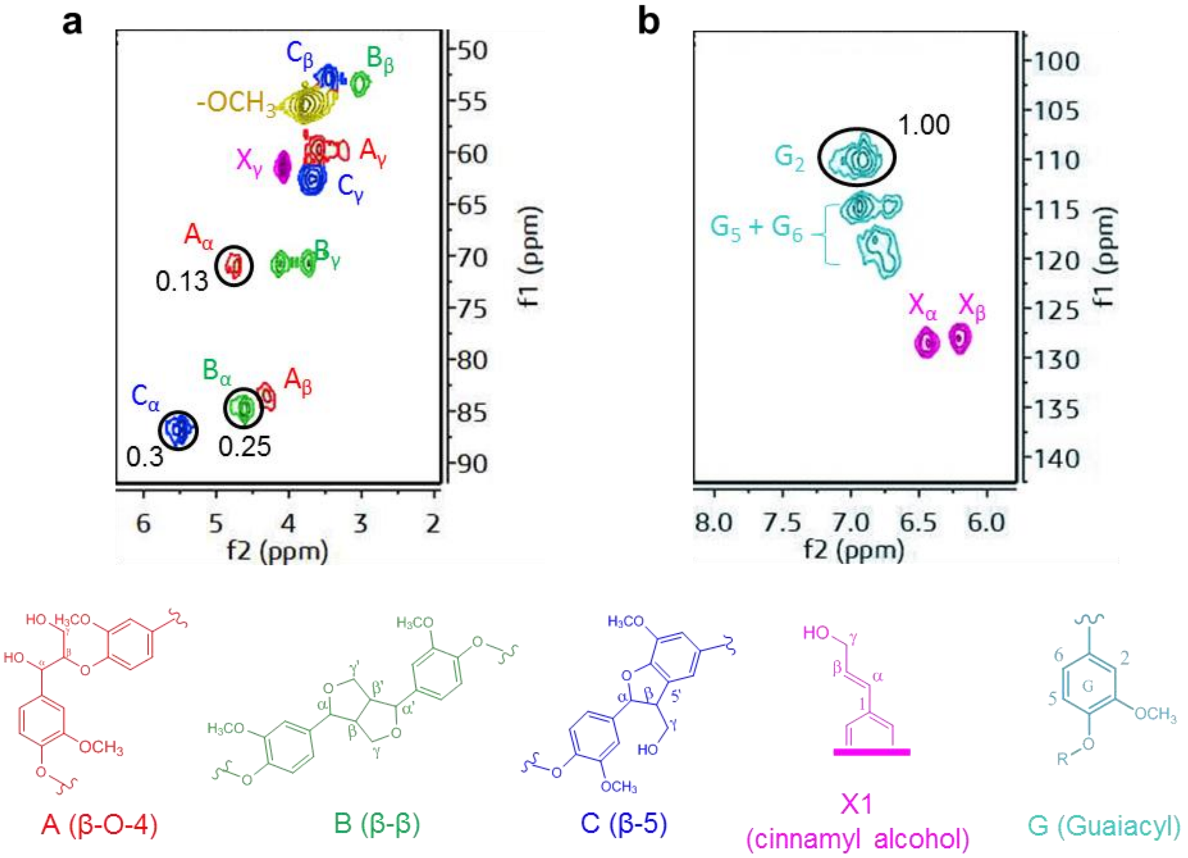

c

The major linkage types	Signal integral intensity	Content (%)
β-O-4	0.13	13.00
β-β	0.25	12.50
β-5	0.30	30.00

Supplementary Fig. 31 | Two-dimensional-nuclear magnetic resonance spectroscopy (2D-NMR) of the polymer. a, Aliphatic region and **b**, aromatic region of 2D ^{13}C-^{1}H HSQC spectra from synthesized polymer in the three-compartment reactor in DMSO-d_6 and **c**, the contents of major linkages relative to the total aromatic units. Contours are colour coded according to the structures shown at the bottom of the figure. Relative signal integrations of the linkages to the aromatic region determined the content of the linkages in polymer structure. G_2 signal was used for G unit. Signal integrations of linkages were estimated from their C_α-H_α correlation, where the signal integration for β-β was logically halved to calculate β-β content.

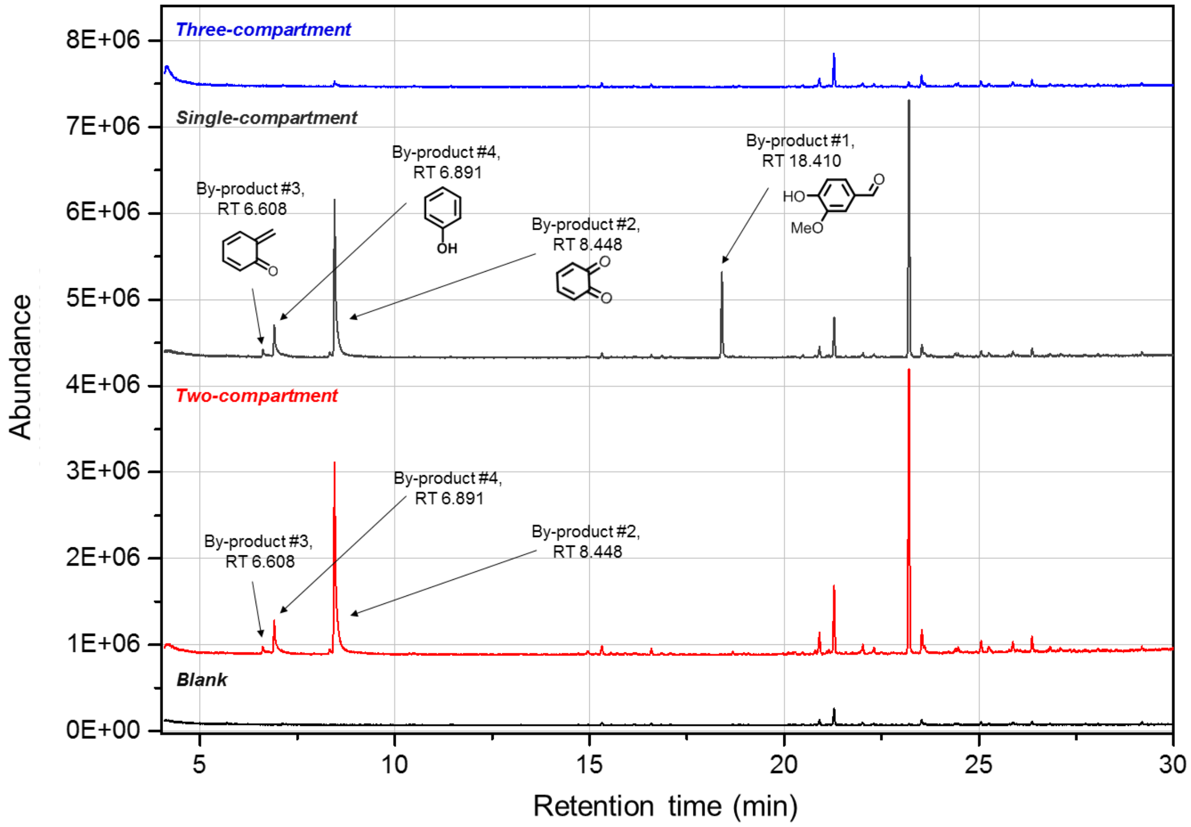

Supplementary Fig. 32 | GC-MS analysis of products from photo-electro-biochemical polymerisation. GC-MS chromatograms of soluble fractions from reaction media of three, single, and two-compartment systems.

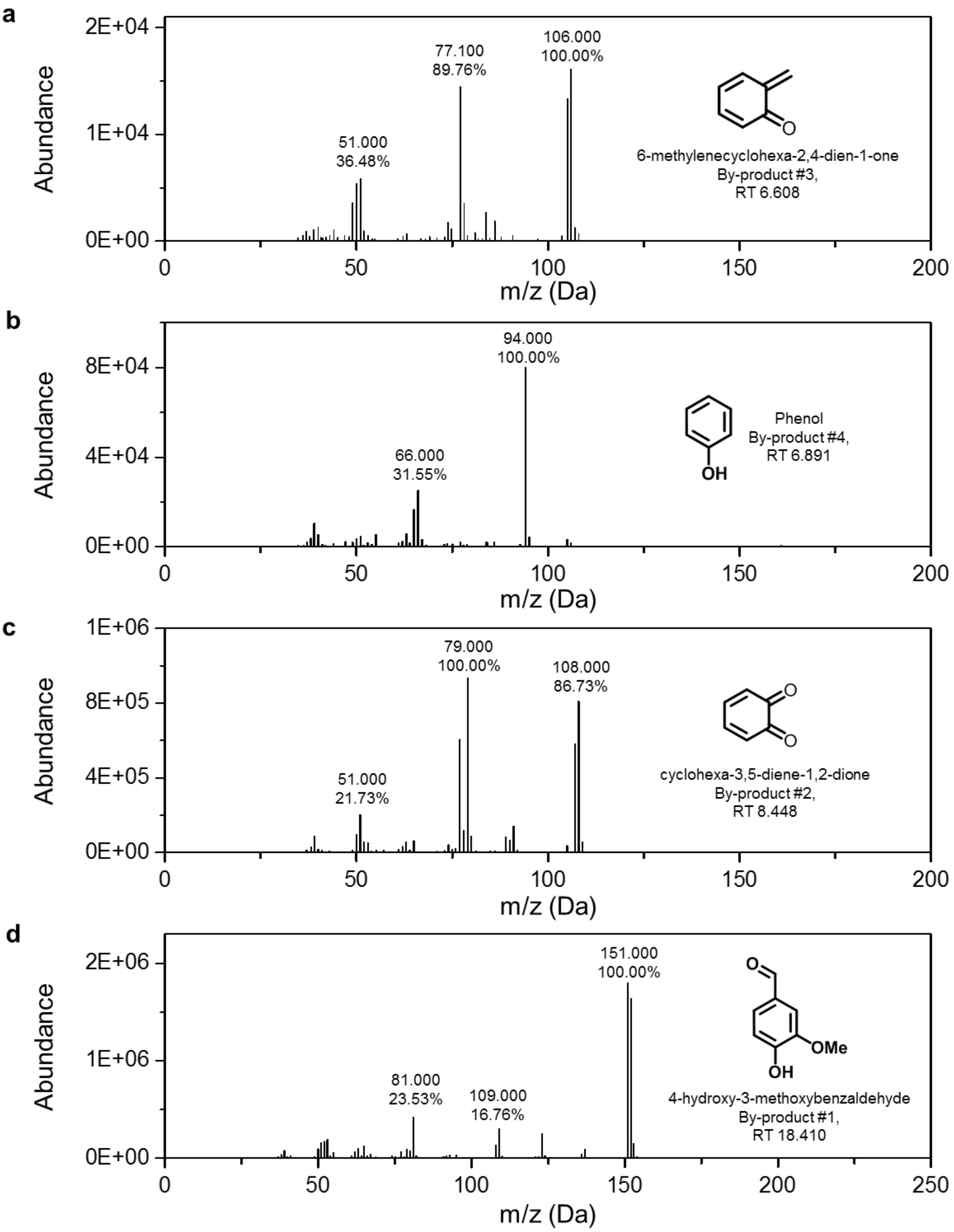

Supplementary Fig. 33 | Mass spectra of the by-products from the photo-electro-biochemical biopolymer synthesis. a, By-products #3 **b**, By-products #4 **c**, By-products #2 **d**, By-products #1.

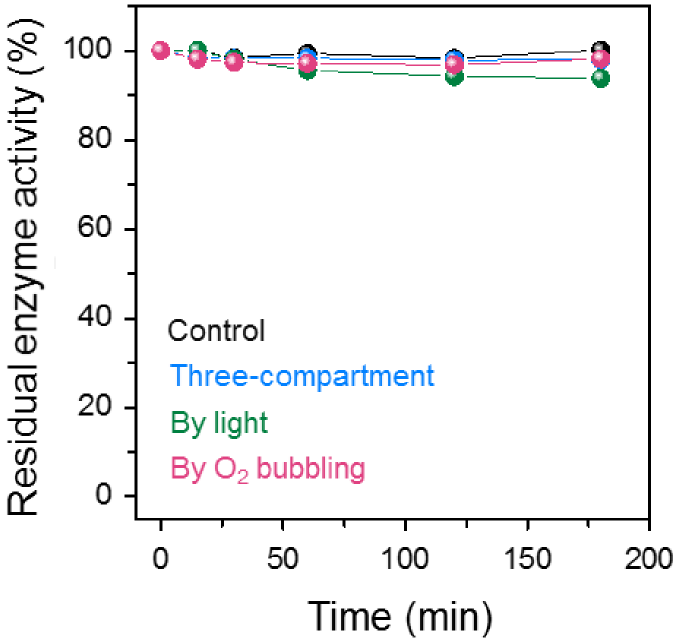

Supplementary Fig. 34 | Enzyme HRP stability test. Effect of O_2 purging and exposure to light irradiation on HRP activity. Residual enzyme activity after incubation with stirring in phosphate borate solution, pH 6.0: without light irradiation and O_2 purging (black circles), with light irradiation (green circles), and with O_2 purging (pink circles) in the two-compartment reactor. The blue circles represent the variation in enzyme activity with light irradiation and O_2 purging in the three-compartment reactor. All experiments were performed with stirring at room temperature.

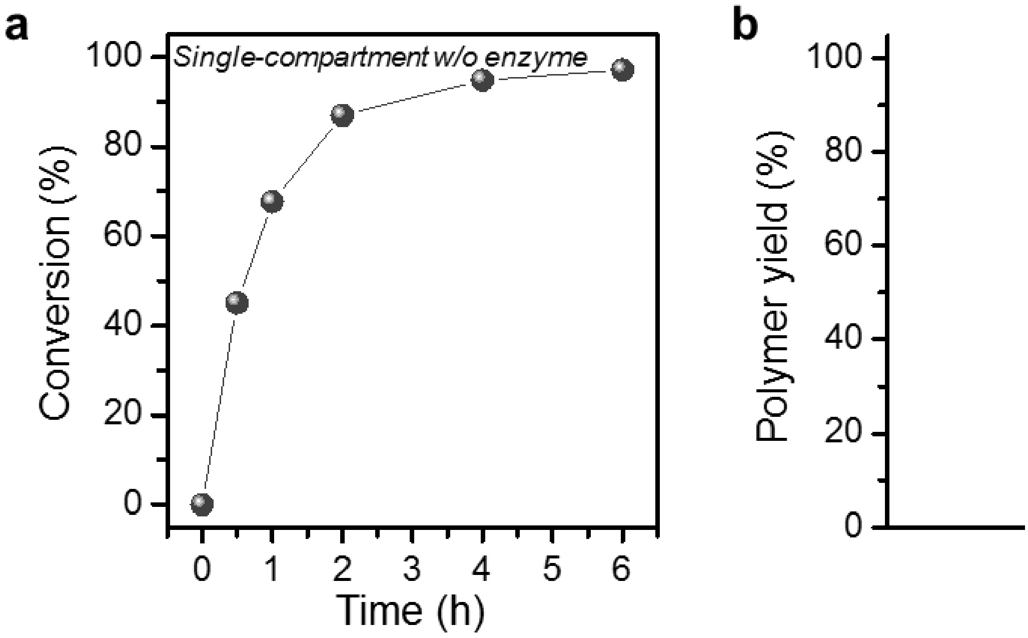

Supplementary Fig. 35 | Photochemical biopolymer synthesis (i.e. without enzyme) in the single-compartment reactor. a, Conversion and **b**, polymer yield. Experimental conditions: 0.1 M phosphate borate solution, pH 6.0, reaction volume 8 mL, catalyst concentration 0.5 mg mL^{-1}, and solar simulator irradiation at 100 mW cm^{-2} (AM1.5G).

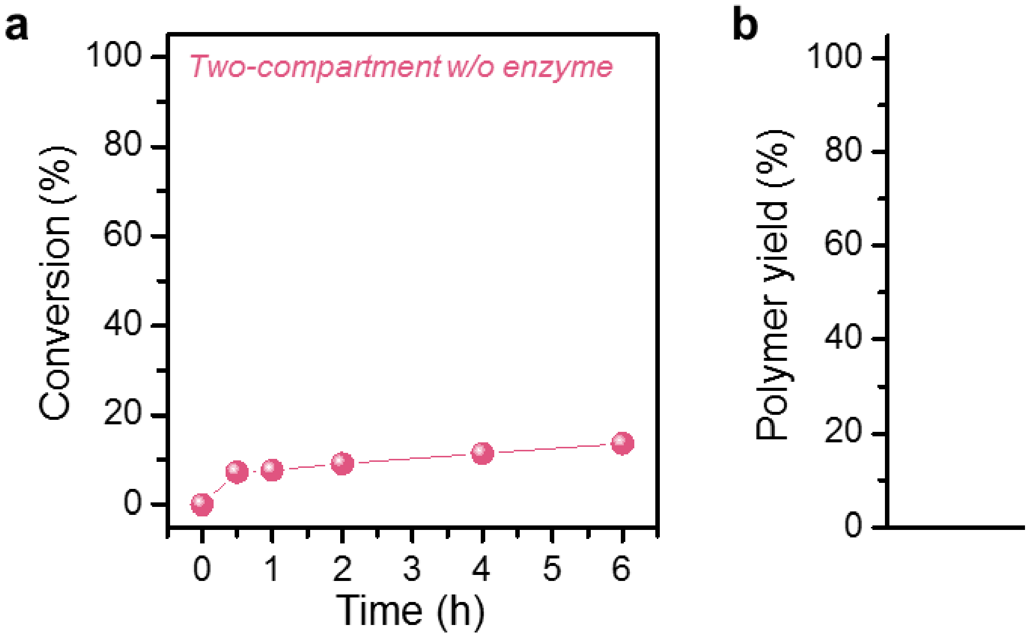

Supplementary Fig. 36 | Photo-electrochemical biopolymer synthesis (i.e. without enzyme) in the two-compartment reactor. a, Conversion and **b**, polymer yield. Experimental conditions: 0.1 M phosphate borate solution, pH 6.0, and solar simulator irradiation at 100 mW cm^{-2} (AM1.5G).

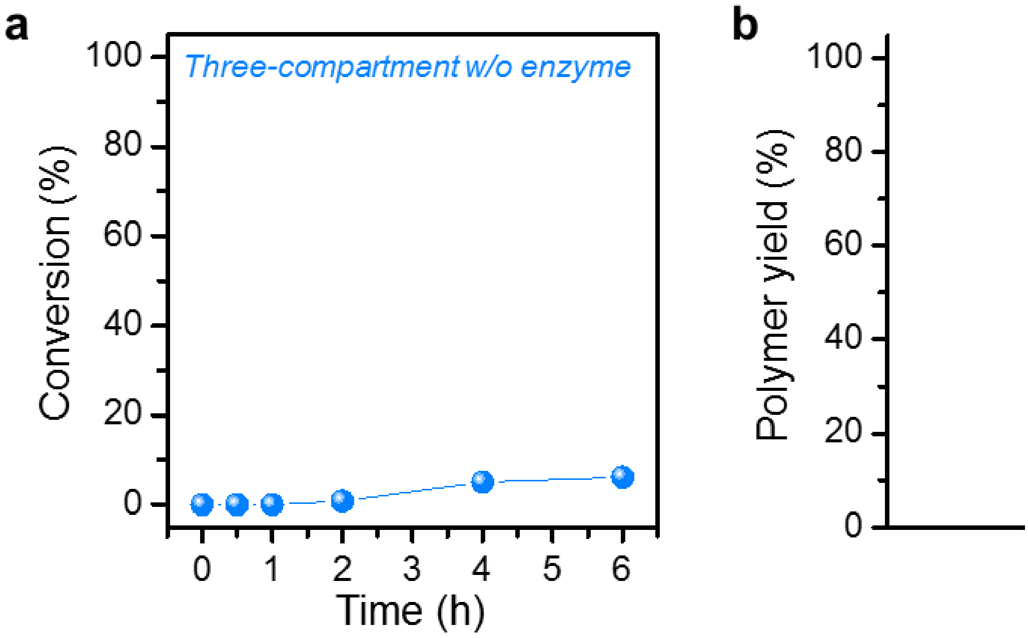

Supplementary Fig. 37 | Photo-electrochemical biopolymer synthesis (i.e. without enzyme) in the three-compartment reactor. a, Conversion and **b**, polymer yield. Experimental conditions: 0.1 M phosphate borate solution, pH 6.0, and solar simulator irradiation at 100 mW cm^{-2} (AM1.5G).
