# Supplementary material for: Unassisted solar lignin valorisation using a compartmented photo-electro-biochemical cell
Source: Nat Commun. 2019 Nov 12;10:5123. doi: 10.1038/s41467-019-13022-7 (PMC6851146; doi:10.1038/s41467-019-13022-7)
Supplement: Supplementary file 2 — Peer Review [file 41467_2019_13022_MOESM2_ESM.pdf]

Reviewers' comments:

Reviewer #1 (Remarks to the Author):

In this work, the authors have reported a photo-electro-biochemical system for lignin valorization without the need for any additional bias or sacrificial agent with good selectivity. The work is interesting and it can be recommended for publication after revisions.

1. In Fig. 2b, the LSV curves for Co–N/CNT turns out to be unnormal below 0.5V, and it seems that after 1000 cycles, this part becomes more efficient. What is the possible reason? Did there any changes happen to the "atomically dispersed Co–Nx sites"? Maybe, STEM images or XAS of the samples before and after the reaction should be provided.
2. How about the DRS of the H:TiO2? As for Supplementary Fig. 4, there is no current response without bias voltage for the H:TiO2 under AM irradiation?
3. In Figure 2d, it seems that the generation rate of H2O2 is not constant, what is the reason? It is might be not proper to say that "We observed the stable and continuous H2O2 production over 6 h of reaction without signs of deactivation" in lines 229-231.
4. According to HPLC analysis,there were many side reactions.What are those by-products?
5. A proposed mechanism for breaking of the lignin structure is suggested to be supplement.
6. According to previous study, the reaction of converting lignin models into aromatic compounds is a free radical reaction. The conversion and selectivity of lignin in the photocatalytic part without LiPH8 are suggested to be supplement so that it is better for readers understand the advantages of three-compartment photo-electro-biochemical system.
7. In lines 242-246, it is claimed that "Given 7.4% of the lignin dimer passed through a cellulose membrane to the cathodic compartment (Supplementary Fig. 12), the actual conversion efficiency could be higher. Although the small lignin dimer used in this experiment diffused through cellulose membrane, such a phenomenon is unlikely to occur for native bulky lignin." It is suggested that the as-claimed actual conversion efficiency can be given, or does it can be resolved, such as timely move out the products? What kind of the "native bulky lignin" do you refer to, and does it can be realized by this reaction system?
8. The expressions in lines 249-253 is a bit reduplicative with that in lines 263-264, which can be improved.
9. In Fig. 3d, the reaction mechanism diagram seems to be not consistent with the results, since the biocatalysts is proved to be not working. In addition, the possible produced ROS should also be added into Fig. 3a,d,g to make the reaction mechanism more clear. In addition, it is wondered that how about the efficiency of the two-compartment powder-based bio-photochemical system?
10. How about the efficiency of this two lignin transformation reactions based on other reaction systems? It is suggested that this work can be compared with previous published works.
11. Recent progress on lignin valorization and related model compounds is missed; for example, see: Green Chem., 2016, 18, 594–607; Chem.Rev. 2015, 115, 10307-10377.
12. In line 224, "the LSV of H:TiO2 and Co-N/CNT was 0.63 mA, which far exceeds that of H:TiO2 and CoTMPP/CNT (0.16 mA), as well as those of H:TiO2 and pristine CNT (0.015 mA)," The calculation of the last two data should be given, like Fig. 2c.
13. In line 248, "suggesting that the biocatalyst is essential for selective lignin valorisation (Supplementary Fig. 13)" Why does the conversion decrease with the increase of reaction time (2-4h)?

Reviewer #2 (Remarks to the Author):

The present paper descibes a novel method for biomass valorisation using an integrated concept of photo-, electro- and biocatalysis. The concept is certainly very intriguing and the interest of the community should be high. However, there is one major conceptual flaw and several smaller issues

which require rewriting major parts of the manuscript or drastically altering the claims. Therefore I recommend rejection of the article but encourage the authors to resubmit a thoroughly reworked version at a later point.

Regarding the major conceptual flaw in the report. The authors claim to perform water oxidation in the (photo)anode cell, yet they use a BR buffer which contains significant amounts of acetate. It is well known that acetate may act as a sacrificial electron donor for TiO_2 and thereby accelerate the reductive reaction. As such, it is not water oxidation that is performed but rather acetate oxidation. Analysis of acetate concentrations as well as the product and potential intermediates of its oxidation (formaldehyde, formate, carbon dioxide) should be performed to exclude this or the claim of water oxidation must be dropped.

Furthermore, the paper should give more information on the overall efficiency of the system, e.g., how much of the H_2O_2 generated is converted by the enzyme to the desired product and what is the fate of the remainder. How high is the TTN and turnover rate of the enzymes and how does that compare to other reported values for these enzymes, i.e., how efficiently are they utilized in the present system. How does the efficiency compare to just adding a (dilute) H_2O_2 solution to the lignin/enzyme solution.

The RRDE experiments indicate that oxygen is reduced in a two-electron reduction which directly yields hydrogen peroxide (in contrast to the one-electron reduction yielding superoxide). Yet the authors blame superoxide/hydroperoxide radicals for some of the observed enzyme deactivation and side-reactions. The authors should experimentally determine superoxide to clarify this.

For the experiments with coniferyl alcohol yielding many by-products it would be beneficial if the authors attempted to identify at least the major peaks in their chromatograms in order to give an idea of the possible mechanisms taking place.

Lastly, the authors should mention that many other groups have successfully integrated photocatalytic H_2O_2 generation with enzymes (e.g. peroxydases and peroxigenases).

Reviewer #3 (Remarks to the Author):

In the manuscript „Unassisted solar lignin valorisation using a compartmented photo-electro-biochemical cell" the author claimed that water was oxidized in the BR-buffer. The Britton-Robinson buffer contains different components, which could act as sacrificial agents.

Before the acceptance of the paper could be taken into consideration the authors must investigate if really an unassisted water oxidation takes place.

In the manuscript authors claim that in their system lignin is converted efficiently, this cannot be claimed:
- only very low concentrations of the substrate are used
- not lignin but "only" one model component is applied
- For a comparison with other methods, the entire process chain must be taken into account (here, for example, the synthesis and service life of the photo anode were not taken into account)
- the scale-ability of the system must be addressed

Line 270 – the unspecific conversion seems to be much more efficient.

The novelty of the manuscript is limited – in my opinion the authors just combined published steps in one system. Therefore, I suggest you reject the paper.

Point-by-point reply to the referee comments

We would like to first sincerely thank all referees for their feedback, questions and suggestions to further improve our manuscript. In the revised manuscript, we have fully taken into account the reviewers' constructive comments. The most critical concern raised by reviewers was the use of acetate in the Britton-Robinson (BR) buffer solution, which can act as sacrificial agent. To rule out the potential effects of acetate as a sacrificial agent, we performed most of the experiment again in acetate-free phosphate borate solution (revised Fig. 2–4, revised Supplementary Fig. 8, 12, 14–17, 23, 24 and 34–37). These results indicate that unassisted lignin valorisation is also possible in the acetate-free solution under sunlight irradiation using the compartmented photo-electro-biochemical cell. We have uploaded highlighted and unhighlighted copies of the revised manuscript and supplementary information. All the changes have been highlighted in dashed red (deleted part) and blue (added part).

Response to Comments of Reviewer #1

General Comment: In this work, the authors have reported a photo-electro-biochemical system for lignin valorization without the need for any additional bias or sacrificial agent with good selectivity. The work is interesting and it can be recommended for publication after revisions.

Response: Thank you very much for the reviewer's endorsement of our manuscript.

Comment 1: In Fig. 2b, the LSV curves for Co–N/CNT turns out to be unnormal below 0.5V, and it seems that after 1000 cycles, this part becomes more efficient. What is the possible reason? Did there any changes happen to the "atomically dispersed Co–Nx sites"? Maybe, STEM images or XAS of the samples before and after the reaction should be provided.

Response: We thank the reviewer for the critical comment. It should be pointed out that the current at high overpotentials (e.g. below 0.5 V) is limited by the mass-transport of O_2 dissolved in the electrolyte. In addition, the peroxide current (shown in Fig. 2b) is determined by the amount of transported peroxide, which is generated by the catalyst, from the disk to ring electrodes (Review Only Fig. 1).

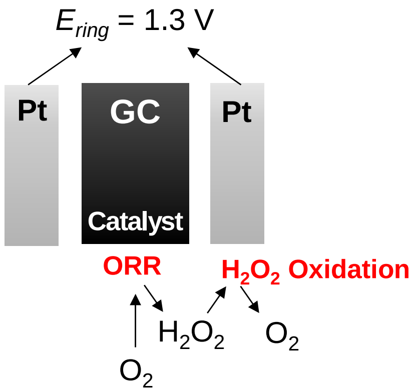

Review Only Fig. 1 | Schematic illustration of the rotating ring disk electrode (RRDE) experiment to determine the peroxide current

Therefore, the current in the potential region is governed by dynamic diffusion behaviour of the reactant and products, rather than the nature of the active sites. The slight change in diffusion property inside/in the vicinity of the catalyst layer could be the reason for the ca. 10% increase of the diffusion-limited current after 1000 cycles. It is possible that oxygen-related functional groups created on the catalyst surface by the applied potential can affect the transport behaviour, and in turn the peroxide current.

During the revision, we repeated the cycling test in an acetate-free phosphate borate solution, as suggested by the other reviewers. In this measurement, as can be seen in the Revised Fig. 2b, the abnormal shape of LSV was not found below 0.5 V. Although there is a marginal increase in the overpotential, we believe that it would lead to a negligible decrease in the operation current. The durability test result was consistent with the original one, indicating that our Co–N/CNT cathode catalyst is durable under the peroxide generation reaction conditions.

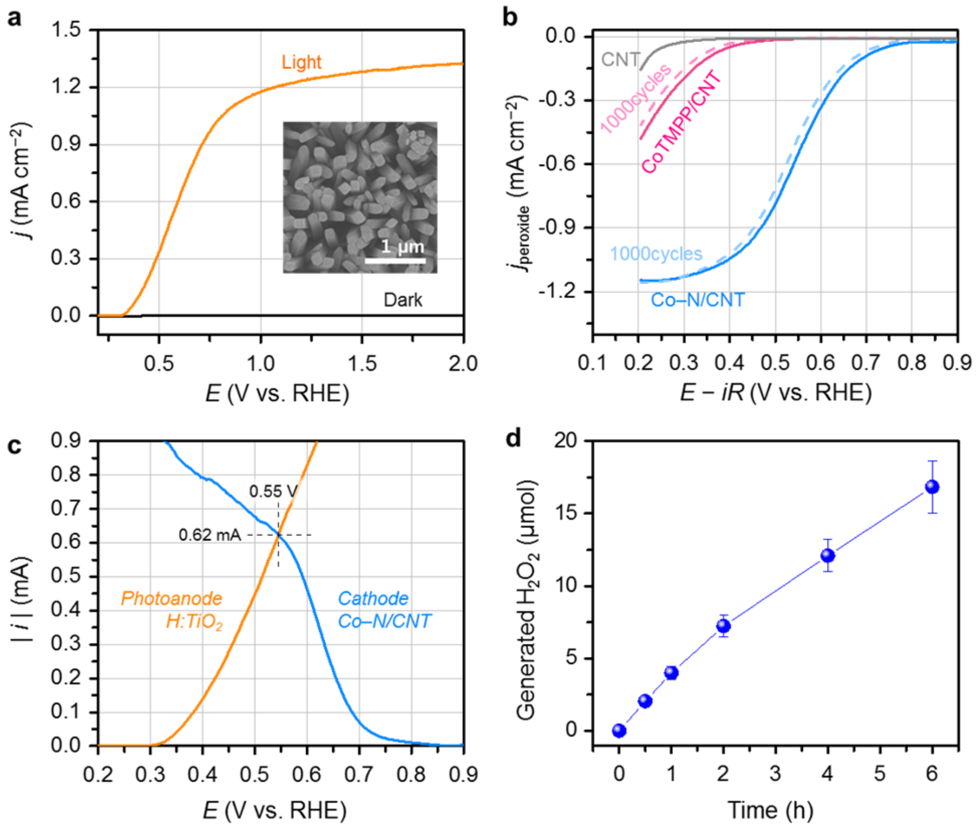

Revised Fig. 2 | Photo-electrocatalytic production of H_2O_2 in the integrated three-compartment reactor. a, Photocurrent density of the H:TiO_2 electrode under illumination and in the dark in 0.1 M phosphate borate solution at pH 4.5. Inset: SEM image of H:TiO_2. **b**, Linear sweep voltammetry curves of Co–N/CNT, CoTMPP/CNT, and CNT catalysts before and after potential cycling tests. The current densities for H_2O_2 production are shown and were obtained via rotating ring disk electrode measurements at an electrode rotation speed of 1,600 rpm in O_2-saturated 0.1 M phosphate borate solution at pH 4.5. **c**, *I-V* curves of H:TiO_2 photoanode under simulated 1 sun (AM1.5G) illumination 0.1 M phosphate borate solution at pH 4.5 and *I-V* curves of Co–N/CNT cathode in O_2-saturated 0.1 M phosphate borate solution at pH 4.5. **d**, Amount of H_2O_2 generated over time in 0.1 M phosphate borate solution at pH 4.5 during the operation of the integrated three-compartment reactor.

We also performed the STEM analysis for Co–N/CNT sample before and after the durability test for H_2O_2 production. STEM images in Supplementary Fig. 5 clearly indicate that there are virtually no changes in the Co–N/CNT catalyst after 1,000 cycles of durability tests, suggesting that no agglomeration took place during the durability test.

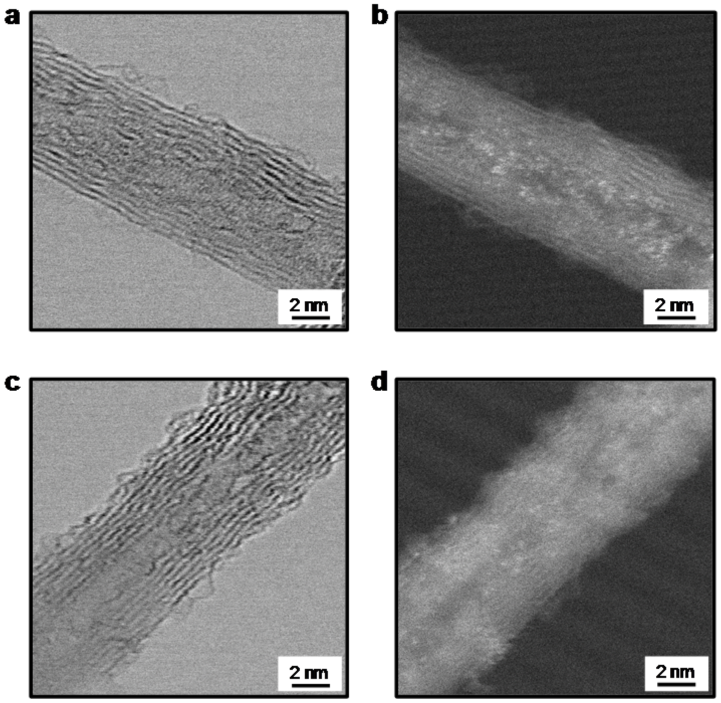

Supplementary Fig. 5 | Scanning transmission electron microscopy (STEM) images of Co–N/CNT before and after the potential cycling tests.

Added contents in the revised manuscript text, Page no. 12

"Scanning transmission electron microscopy (STEM) images of Co–N/CNT before and after the stability test were nearly the same, indicating that atomically dispersed Co species are not agglomerated or detached from CNT support (Supplementary Fig. 5)."

Comment 2: How about the DRS of the H:TiO_2? As for Supplementary Fig. 4, there is no current response without bias voltage for the H:TiO_2 under AM irradiation?

Response: Thank you for the suggestion. UV-vis diffusion reflectance spectra of H:TiO_2 and TiO_2 have been added in Supplementary Fig. 2b. Both films similarly showed a typical response and have no differences, indicating no significant changes in UV-vis spectra upon hydrogen treatment on TiO_2 photoanode.

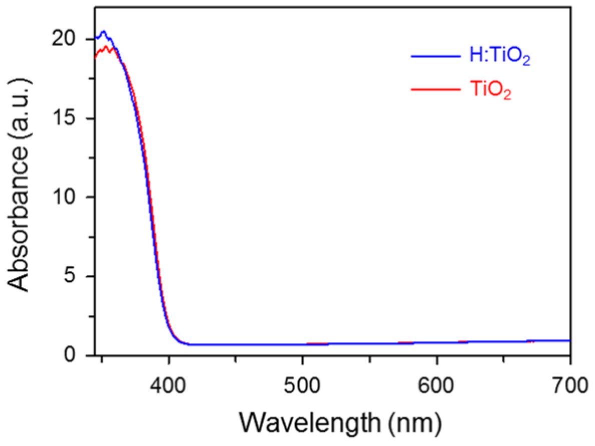

Supplementary Fig. 2b | UV-vis diffusion reflectance spectra (DRS) of H:TiO_2/FTO and TiO_2/FTO.

Added contents in the revised manuscript text, Page no. 8

"There was also no significant change in the TiO_2 UV-vis spectra upon hydrogen treatment (Supplementary Fig. 2b)."

Regarding the latter comment, our photo-electro-biochemical system operates at 0.55 V (Revised Fig. 2), where the photoanode and cathode generate the same current, not 0 V (vs. RHE) in the Supplementary Fig. 4. Bias-free indicates that we can obtain the current when the photoanode and cathode are at equipotential.

Comment 3: In Figure 2d, it seems that the generation rate of H_2O_2 is not constant, what is the reason? It is might be not proper to say that "We observed the stable and continuous H2O2 production over 6 h of reaction without signs of deactivation" in lines 229-231.

Response: We agree with the reviewer's criticism that the H_2O_2 production rate is not constant during the 6 h reaction. As a response, we have toned down the sentence.

Manuscript text, Page no. 11

~~*"We observed the stable and continuous H_2O_2 production over 6 h of reaction without signs of deactivation"*~~

→ *"We observed continuous H_2O_2 production over 6 h of reaction,…"*

Comment 4: According to HPLC analysis, there were many side reactions. What are those by-products?

Response: As the reviewer commented, there are some side products detected by GC-MS analysis. We found that there were five side products and one side product in the single-compartment reactor and two-compartment reactor respectively, while these were not observed in the three-compartment cells (Revised Supplementary Fig. 18). These products may have been formed by direct photocatalysis including the oxidation of the substrate and bond cleavage at C_{alpha}-C_{aryl}, or by indirect reduction. The proposed reaction scheme in single-,two-, and three-compartment system are presented in Supplementary Fig. 21 and 22.

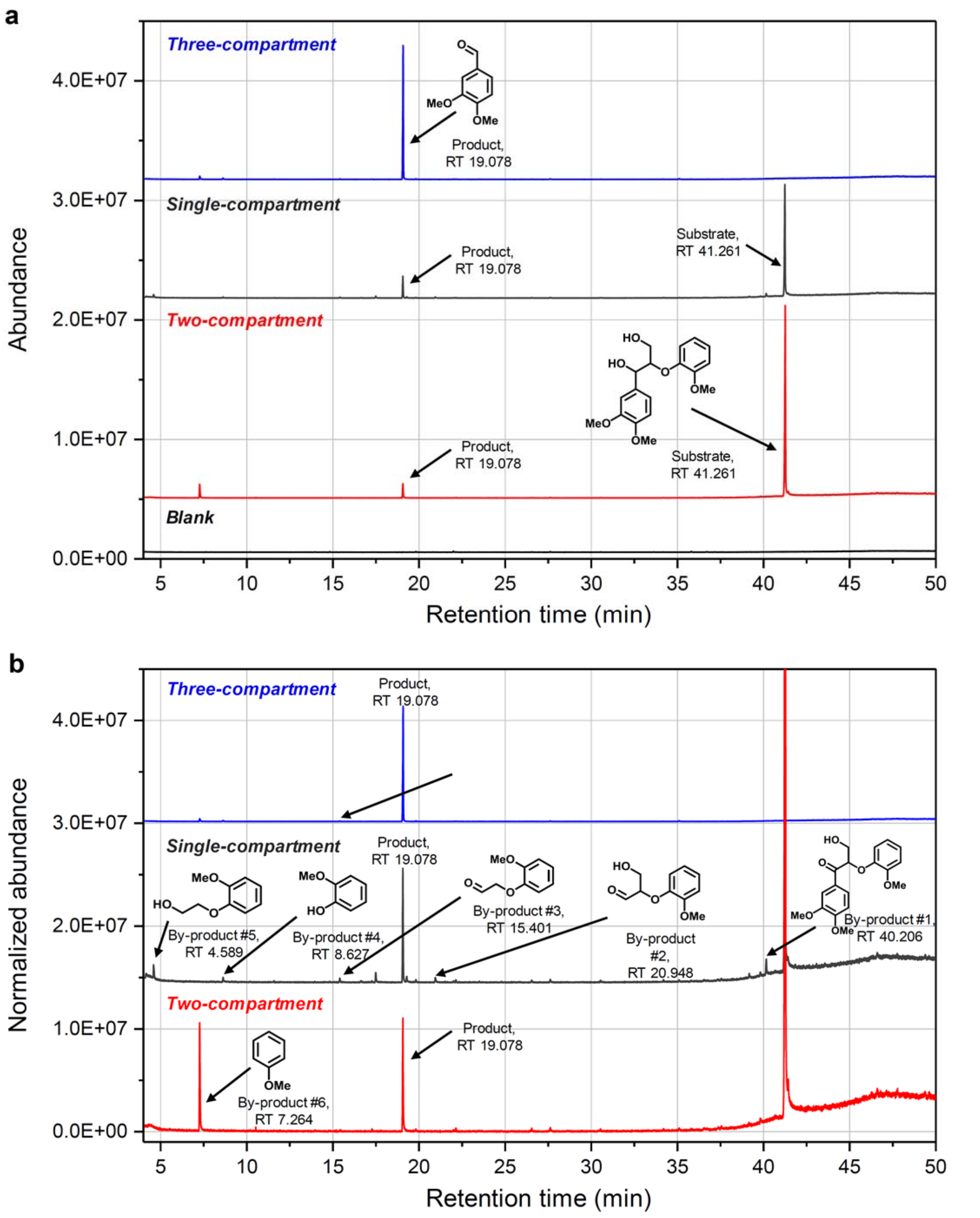

Supplementary Fig. 18 | GC-MS analysis of products from photo-electro-biochemical lignin degradation. a, GC-MS chromatograms of soluble fraction from reaction medium of three, single, and two-compartment systems. **b,** Normalised data of **a** (the product peak is normalised as the same height).

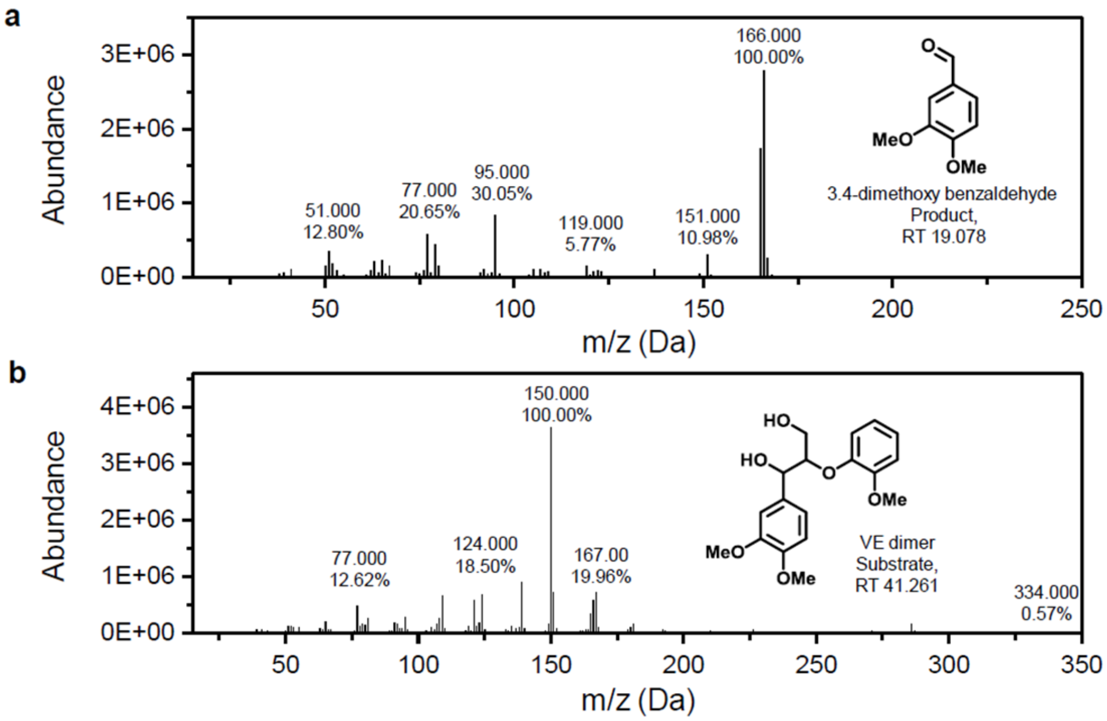

Supplementary Fig. 19 | Mass spectrum of substrate and products identified during photo-electro-biochemical lignin degradation. a, products **b,** substrate.

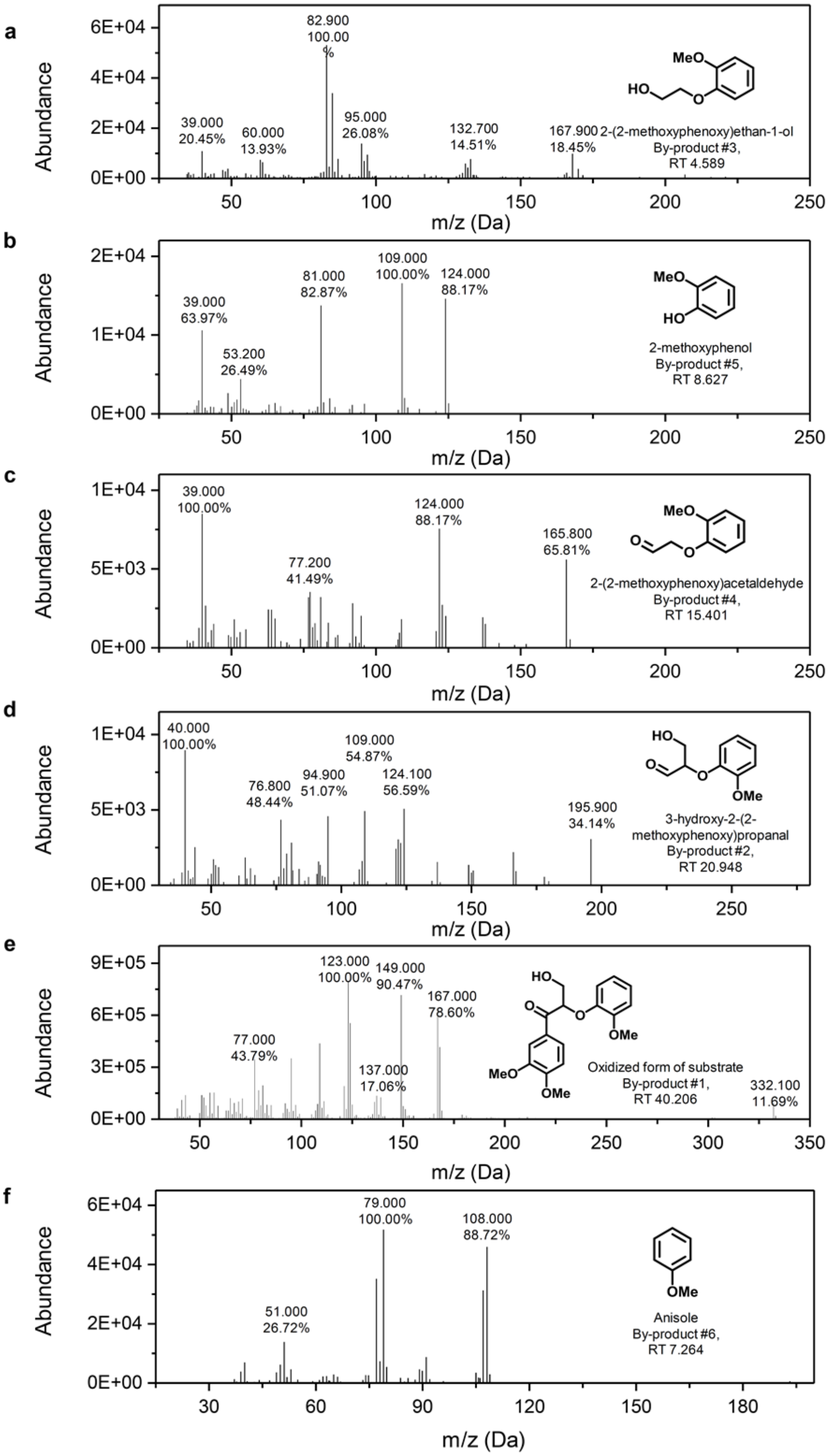

Supplementary Fig. 20 | Mass spectra of the by-products identified from the single-compartment reactor during lignin degradation. a, by-product #3. **b**, by-product #5. **c**, by-product #4. **d**, by-product #2. **e**, by-product #1, **f**, by-product #6.

Comment 5: A proposed mechanism for breaking of the lignin structure is suggested to be supplement.

Response: We thank the reviewer for their suggestion. We have added a possible mechanism in each system as Supplementary Fig. 21 and 22 in the revised supplementary information.

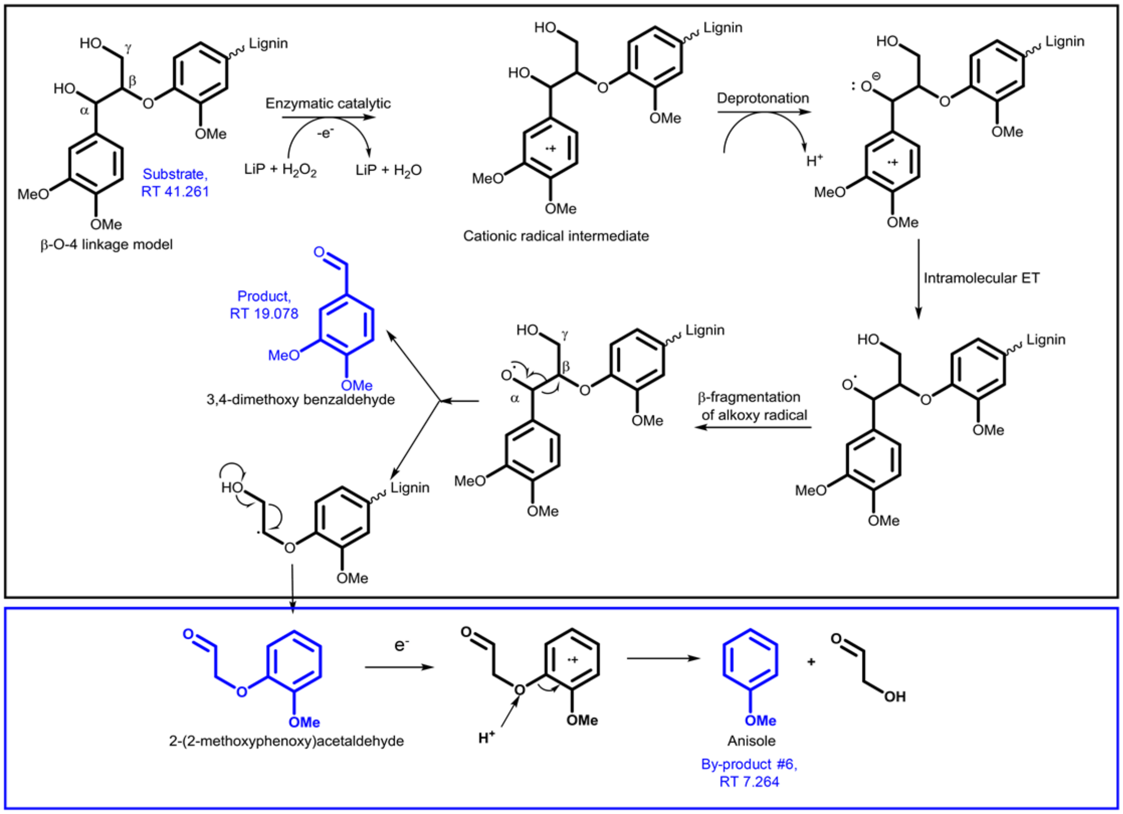

Supplementary Fig. 21 | A proposed mechanism for breaking of the β-O-4 linkage in the lignin structure by the LiPH8 enzyme and H_2O_2 (in black rectangular), and the proposed side reaction in the two-compartment system (in blue rectangular)

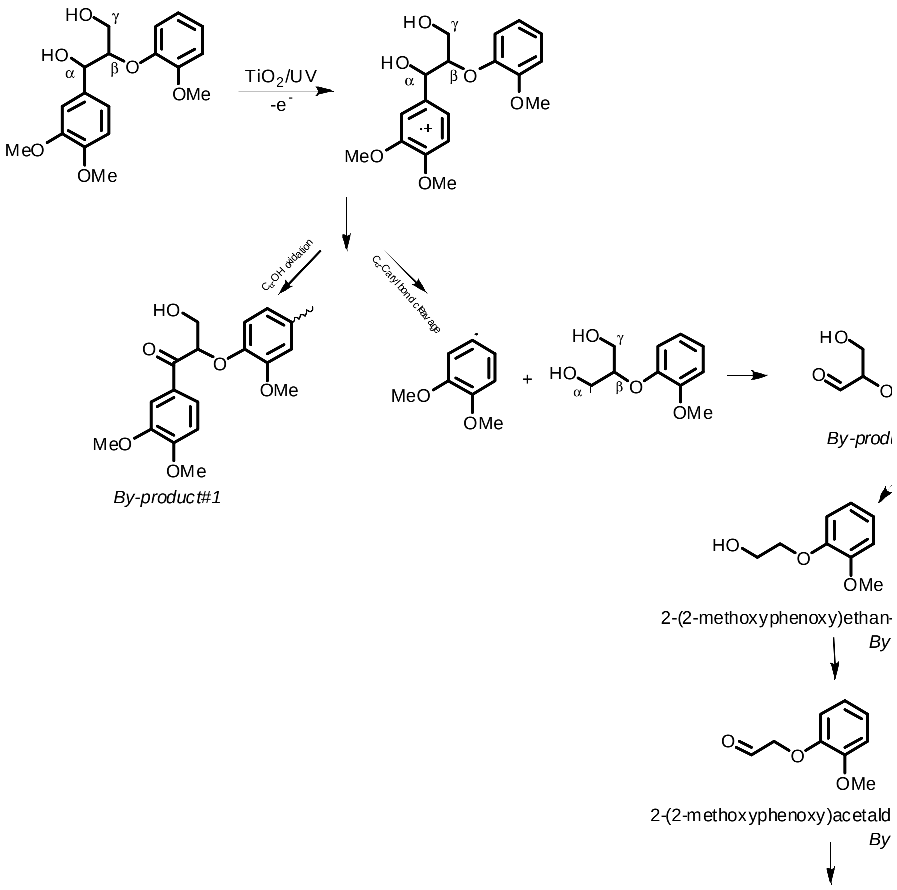

Supplementary Fig. 22 | Scheme for product formation under unselective catalysis of TiO_2/UV on lignin dimer in the single-compartment reactor.

Comment 6: According to previous study, the reaction of converting lignin models into aromatic compounds is a free radical reaction. The conversion and selectivity of lignin in the photocatalytic part without LiPH8 are suggested to be supplement so that it is better for readers understand the advantages of three-compartment photo-electro-biochemical system.

Response: Thank you for the suggestion. We already presented lignin conversion using TiO_2 photocatalyst without LiPH8 in Supplementary Fig. 16 as a control experiment. Interestingly, similar lignin dimer conversion (~44%) and selectivity (~30%) were obtained regardless of using LiPH8 biocatalyst. This result indicates that the lignin conversion in the single-compartment system occurs via direct photocatalysis by the TiO_2 nanoparticles. However, such direct photocatalytic reaction causes many side reactions (~30% selectivity), as shown

by GC-MS (Revised Supplementary Fig. 18–22). This control experiment clearly shows the advantage of the compartmented system to prevent random decomposition reactions and to obtain high conversion and selectivity.

The original statement appears to be insufficient to convey our claim. Therefore, we have revised the sentences for clear explanation.

Manuscript text, Page no. 15

~~*"Interestingly, a similar conversion and selectivity were obtained without LiPH8 (Supplementary Fig. 15), which indicates that the reaction mainly proceeds via direct photocatalytic lignin conversion by the TiO_2 photocatalyst."*~~

→ *"To investigate the role of the enzyme in the single-compartment system, we performed the lignin conversion experiment in the absence of LiPH8. Interestingly, similar conversion and selectivity were obtained without LiPH8 (Supplementary Fig. 16), which ..."*

Comment 7: In lines 242-246, it is claimed that "Given 7.4% of the lignin dimer passed through a cellulose membrane to the cathodic compartment (Supplementary Fig. 12), the actual conversion efficiency could be higher. Although the small lignin dimer used in this experiment diffused through cellulose membrane, such a phenomenon is unlikely to occur for native bulky lignin." It is suggested that the as-claimed actual conversion efficiency can be given, or does it can be resolved, such as timely move out the products? What kind of the "native bulky lignin" do you refer to, and does it can be realized by this reaction system?

Response: The conversion efficiency reported in this manuscript was calculated without considering the amount of lignin dimer passed through the cellulose membranes to the cathodic compartment. In the photocatalytic system with a new acetate-free solution, 5.0% lignin dimer passed through the membrane to the cathode compartment (Review Only Fig. 2).

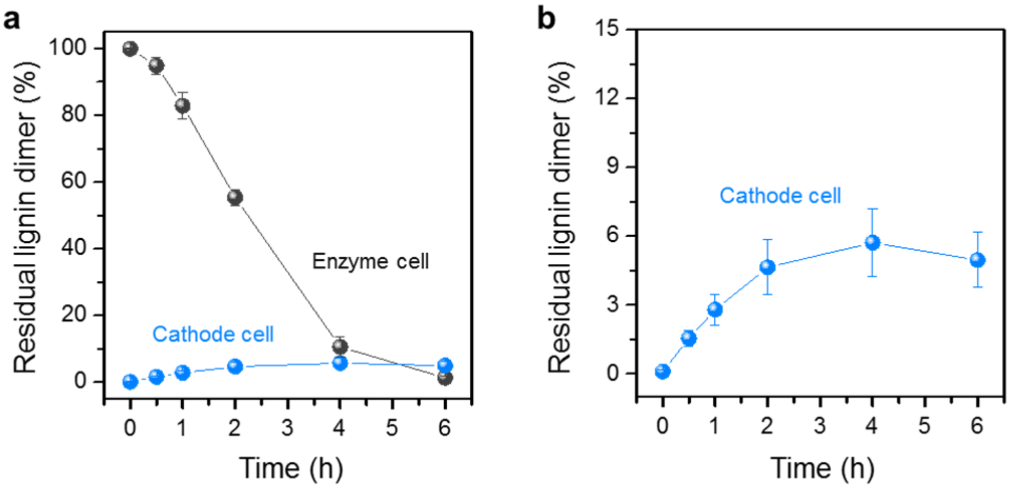

Review Only Fig. 2 | Residual lignin dimer at each cell. a, Residual lignin dimer (%) in the cathode cell and enzyme cell during the course of photo-electro-biochemical reaction in the three-compartment reactor. Residual lignin dimer in enzyme cell (●), and the lignin dimer diffused into the cathode cell (●). **b,** Residual lignin dimer (%) in the cathode cell during the course of photo-electro-biochemical reaction in the three-compartment reactor.

The size of native bulky lignins (milled wood lignin or kraft lignin) is generally between 320~360 nm, which is larger than pore size of a cellulose membrane. Hence, we claimed that they cannot move to enzyme side from cathode side through the membrane [*Green Chem.* **18**, 1416 (2016); *Int. J. Mol. Sci.* **18**, 1244 (2017)]. However, we found that these claims could be confusing, thus we removed this part in the revised manuscript.

Manuscript text, Page no. 13

~~*Given 7.4% of the lignin dimer passed through a cellulose membrane to the cathodic compartment (Supplementary Fig. 12), the actual conversion efficiency could be higher. Although the small lignin dimer used in this experiment diffused through cellulose membrane, such a phenomenon is unlikely to occur for native bulky lignin.*~~

Comment 8: The expressions in lines 249-253 is a bit reduplicative with that in lines 263-264, which can be improved.

Response: We thank the reviewer for pointing this out. As a response, we have deleted the reduplicative sentences.

Manuscript text, Page no. 12

~~*"We compared the three-compartment photo-electro-biochemical system with single-*~~

~~compartment (simple biocatalyst integrated powder-type TiO_2 photocatalyst, Fig. 3d) and two-compartment (H:TiO_2 photoanode is separated, but the Co-based electrocatalyst and biocatalyst are in the same compartment, Fig. 3g) systems with respect to their conversion efficiencies and selectivities."~~

Comment 9: In Fig. 3d, the reaction mechanism diagram seems to be not consistent with the results, since the biocatalysts is proved to be not working. In addition, the possible produced ROS should also be added into Fig. 3a,d,g to make the reaction mechanism more clear. In addition, it is wondered that how about the efficiency of the two-compartment powder-based bio-photochemical system?

Response: As per the reviewer's comment, we have modified the reaction scheme in Fig. 3a,d,g for the lignin degradation and Fig. 4a,d,g for the biopolymer synthesis (Revised Fig. 3 and 4). For the GC-MS analysis in the biopolymer synthesis, we have moved GC-MS chromatogram figures to Supplementary Fig. 32,33.

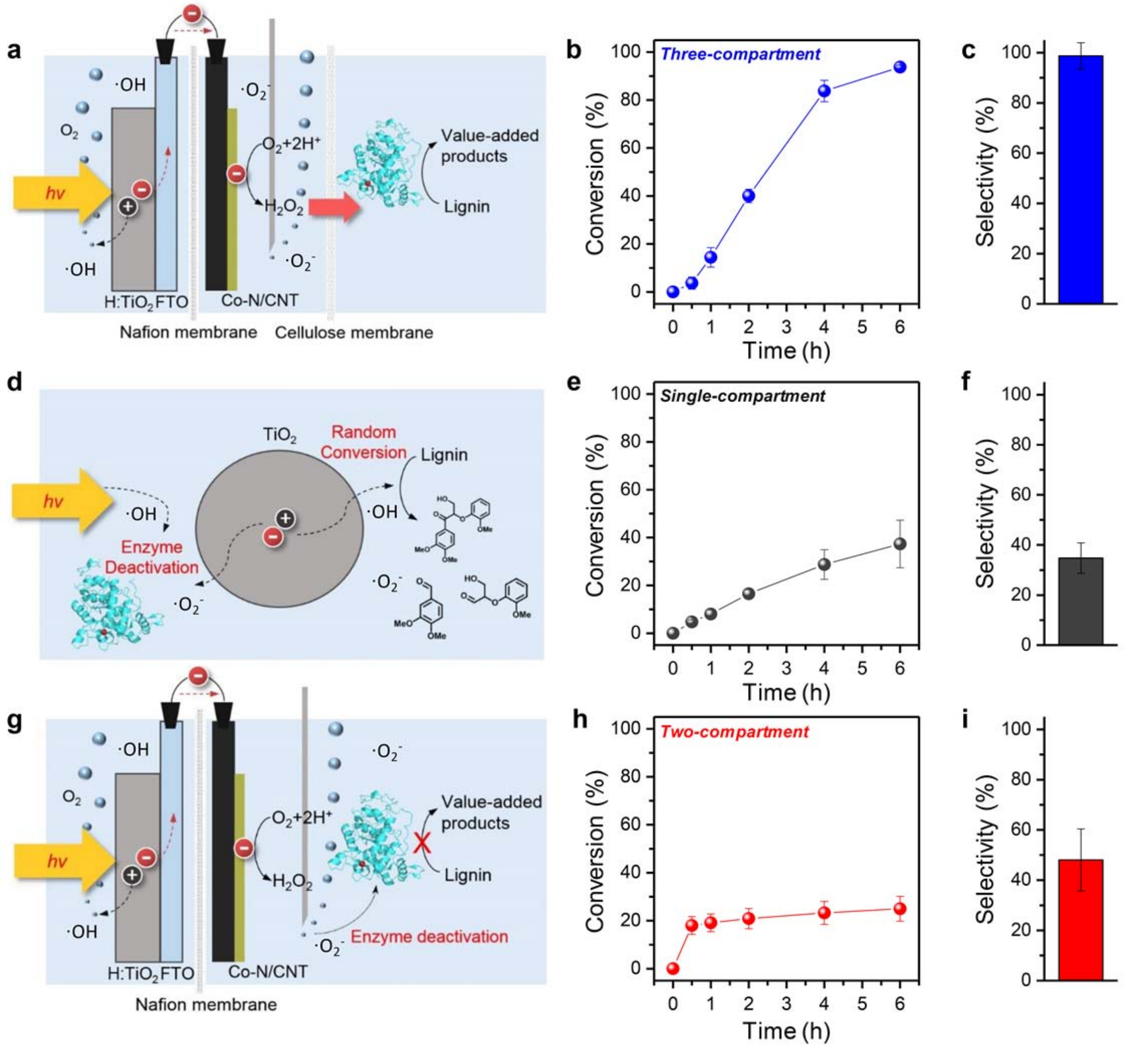

Revised Fig. 3 | Reactor configurations and photo-electro-biochemical lignin dimer conversion. a,**d**,**g**, Schematic representations of the three-compartment photo-electro-biochemical system (**a**), single-compartment powder-based bio-photochemical system (**d**), and two-compartment photo-electrochemical system (**g**) used for lignin dimer conversion. **b**,**e**,**h**, Conversion and **c**,**f**,**i**, selectivity of the three-compartment photo-electro-biochemical system (**b**,**c**), single-compartment powder-based bio-photochemical system (**e**,**f**), and two-compartment photo-electrochemical system (**h**,**i**).

₩

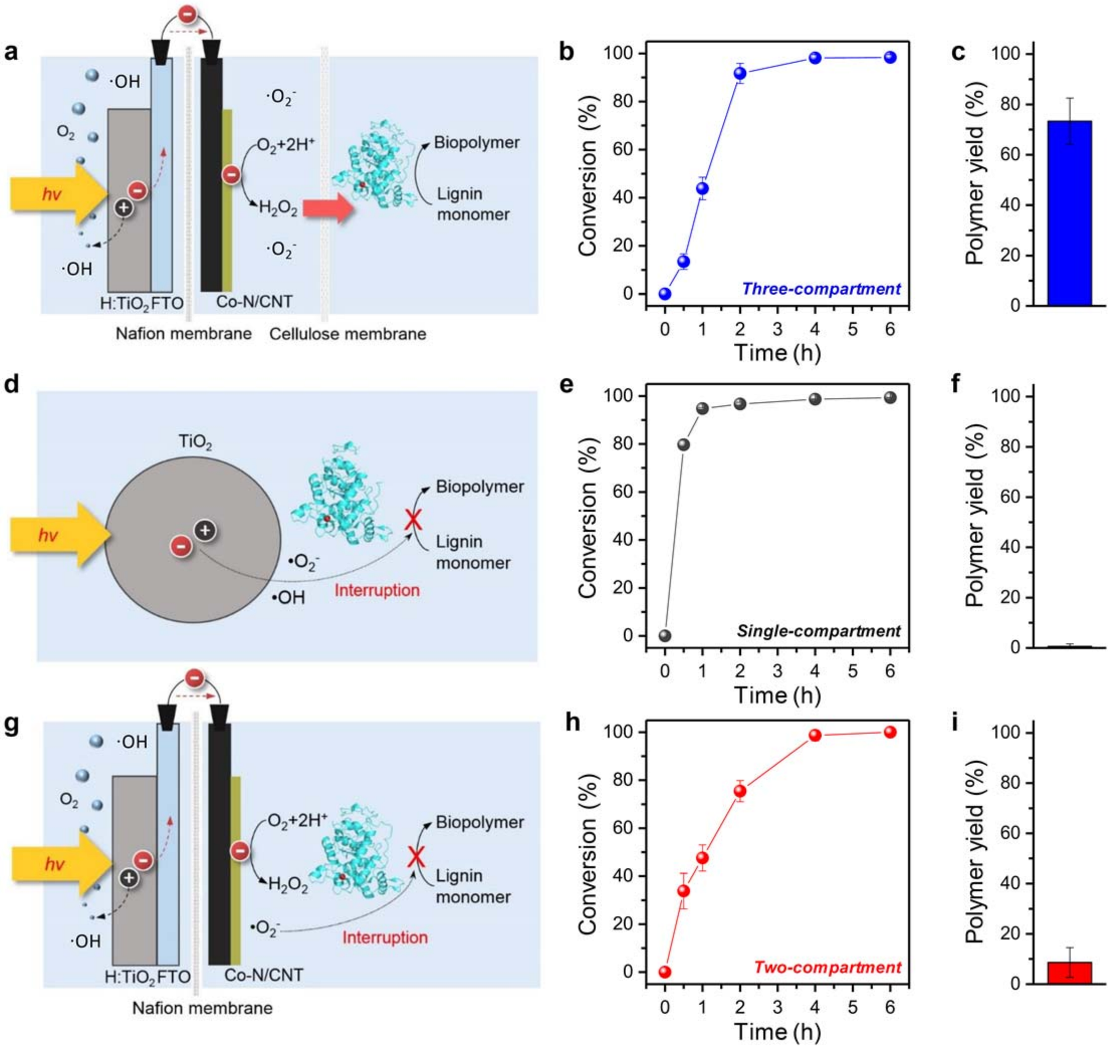

Revised Fig. 4 | Reactor configurations and photo-electro-biochemical synthesis of biopolymer from coniferyl alcohol. **a**,**d**,**g**, Schematic representations of the (**a**), three-compartment photo-electro-biochemical system, (**d**), single-compartment powder-based bio-photochemical system, and (**g**), two-compartment photo-electrochemical system used for biopolymer synthesis. **b**,**e**,**h**, Conversion percentage of coniferyl alcohol over time and **c**,**f**,**i**, polymer yield after 6 h of reaction of the three-compartment photo-electro-biochemical system (**b**,**c**), single-compartment powder-based bio-photochemical system (**e**,**f**), and two-compartment photo-electrochemical system (**h**,**i**).

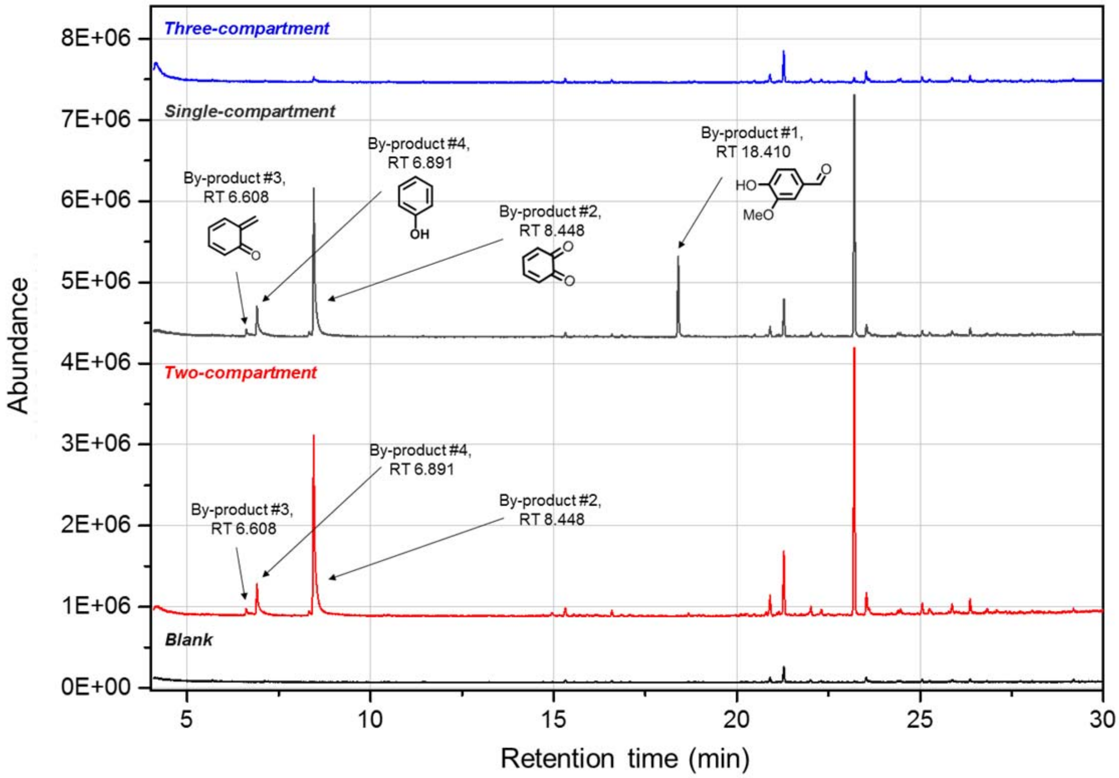

Supplementary Fig. 32 | GC-MS analysis of products from photo-electro-biochemical polymerization. GC-MS chromatograms of soluble fractions from reaction media of three, single, and two-compartment systems.

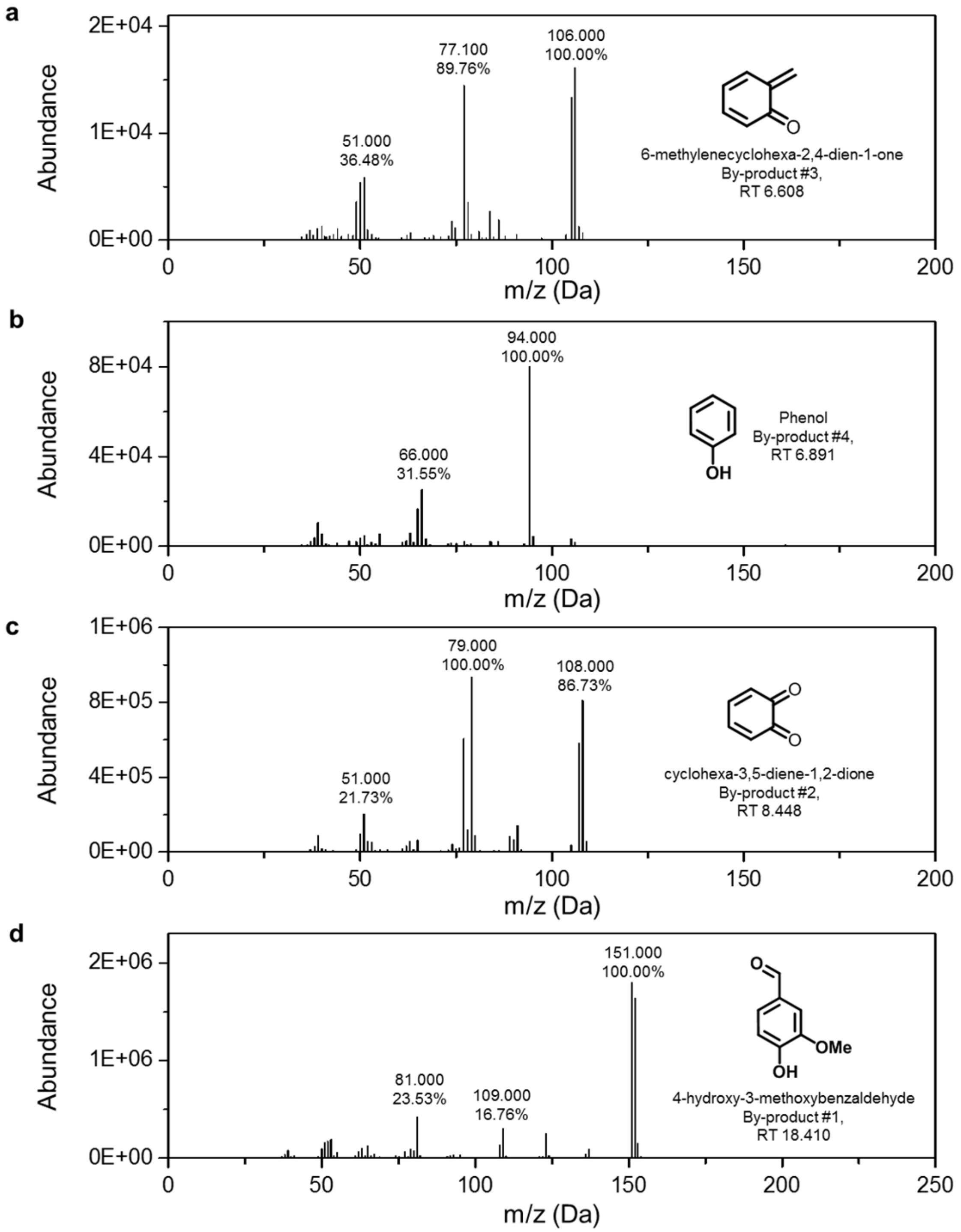

Supplementary Fig. 33 | Mass spectra of the substrate and products identified during photo-electro-biochemical biopolymer synthesis. a, By-products #3 **b**, By-products #4 **c**, By-products #2 **d**, By-products #1.

We also thank the reviewer for suggesting the two-compartment powder-based bio-

photochemical system. We performed lignin degradation in a two-compartment cell consisting of a powder cell (P25) and enzyme cell with a cellulose membrane between two cells. The conversion and selectivity of lignin degradation were 22% and 42%, respectively (Review Only Fig. 3). Because the lignin dimer as well as H_2O_2 permeate the cellulose membrane, it is photocatalytically degraded by the TiO_2 powder, resulting in low selectivity. In addition, powder photocatalyst cannot block the penetration of irradiated light into the enzyme compartment completely, unlike the film type of photocatalyst, so that there would be enzyme deactivation by the light.

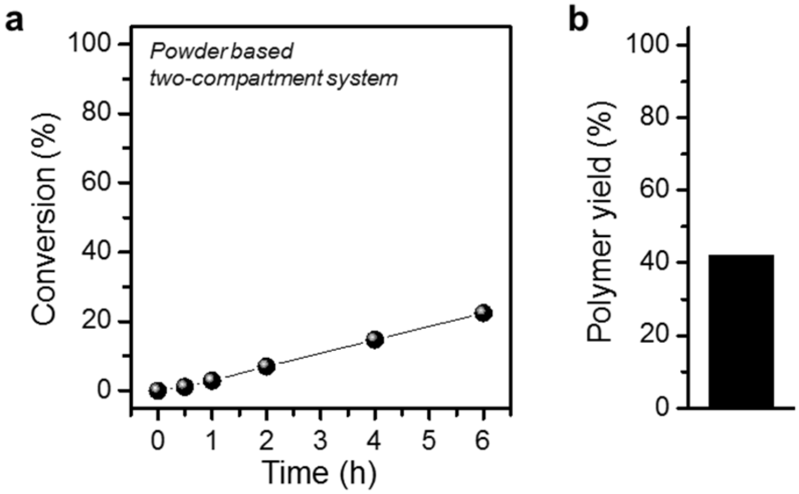

Review Only Fig. 3 | Bio-photochemical lignin degradation in powder based two-compartment reactor. a, Conversion and **b**, selectivity.

Comment 10: How about the efficiency of this two lignin transformation reactions based on other reaction systems? It is suggested that this work can be compared with previous published works.

Response : Please see our response to reviewer 2's second comment.

Comment 11: Recent progress on lignin valorization and related model compounds is missed; for example, see: Green Chem., 2016, 18, 594–607; Chem.Rev. 2015, 115, 10307-10377.

Response: We thank the reviewer for suggesting relevant literature for this paper. In our original manuscript, we already cited *Green Chem.* **2016**, *18*, 594–607. Following the reviewer's suggestion, we have added *Chem. Rev.* **2015**, *115*, 10307–10377 and *Appl. Catal., B* **2012**, *111*, 555-561. We believe that this addition would be helpful for potential readers of this paper.

36 Zhang, N., Yang, M.-Q., Liu, S., Sun, Y. & Xu, Y.-J. Waltzing with the versatile platform of graphene to synthesize composite photocatalysts. *Chem. Rev.* **115**, 10307-10377 (2015).

44 Augugliaro, V. *et al.* Synthesis of vanillin in water by TiO_2 photocatalysis. *Appl. Catal., B* **111**, 555-561 (2012).

Manuscript text, Page no. 3

Various treatment technologies including physical (ball milling, ultrasonication, plasma irradiation, and microwave heating), chemical (using an organic or inorganic acid), or catalytic (with a heterogeneous catalyst) methods have been used for lignin valorisation[18-21]. *However, these processes are usually energy-intensive, requiring high temperatures and pressures, and environmentally unfriendly, producing chemical waste.*

The above content is related to the lignin valorisations which is already included in the manuscript (*Green Chem.* **2016**, *18*, 594–607). We added the contents related to the recent progress on lignin valorisation and related model compounds as follows:

Manuscript text, Page no. 5

However, this integrated system (biocatalytic system with photocatalytic H_2O_2) has been rarely applied for lignin valorisation, and most of the research has been focused on developing an efficient powder-type photocatalyst such as using Nb_2O_5/TiO_2 heterojunction, metal-doped TiO_2, graphene-TiO_2 nanocomposites Pt metal core CeO_2 photocatalyst for direct lignin conversion not for H_2O_2 production[12,36].

Manuscript text, Page no. 13

There are many reported lignin model compounds such as ferulic acid, vanillyl alcohol, and isoeugenol and benzyl alcohol[12,44]. *We selected lignin dimer as a representative model compound to specifically observe the cleavage of β–O–4.*

Comment 12: In line 224, "the LSV of H:TiO2 and Co-N/CNT was 0.63 mA, which far exceeds that of H:TiO2 and CoTMPP/CNT (0.16 mA), as well as those of H:TiO2 and pristine CNT (0.015 mA)," The calculation of the last two data should be given, like Fig. 2c.

Response: We have added new Supplementary Fig. 10 showing LSV curves of CoTMPP/CNT, pristine CNT, and H:TiO_2 for the estimation of operation currents. In a new acetate-free system, the photoelectrochemical reactor produced 0.62, 0.09, and 0.07 mA with Co–N/CNT, CoTMPP/CNT, and CNT cathodes, respectively, emphasising the importance of an active cathode in our system.

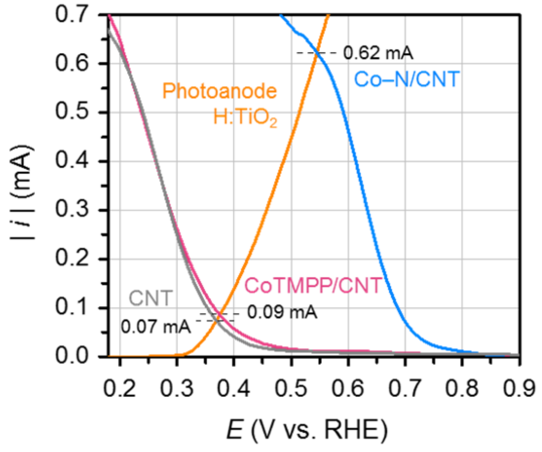

Supplementary Fig. 10 | *I-V* curve of H:TiO_2 photoanode under simulated 1 sun (AM1.5G) illumination in 0.1 M phosphate borate solution at pH 4.5 and *I-V* curves of Co–N/CNT, CoTMPP/CNT, and CNT cathodes in O_2-saturated 0.1 M phosphate borate solution at pH 4.5.

Comment 13: In line 248, "suggesting that the biocatalyst is essential for selective lignin valorisation (Supplementary Fig. 13)" Why does the conversion decrease with the increase of reaction time (2-4h)?

Response: We carried out the same experiment again in phosphate borate solution at pH 4.5 in the absence of enzyme (Revised Supplementary Fig. 15) and we found that the conversion is constant during the reaction (2-4 h).

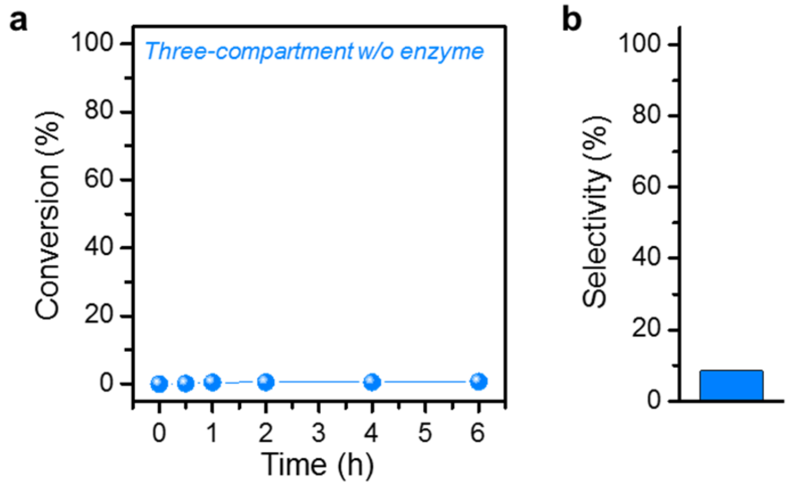

Revised Supplementary Fig. 15 | Photo-electrochemical lignin degradation (i.e. without enzyme) in the three-compartment acryl reactor equipped with quartz window. a,b, Lignin conversion (**a**) and selectivity (**b**). Experimental conditions: 0.1 M phosphate borate solution, pH 4.5, and solar simulator irradiation at 100 mW cm^{-2} (AM1.5G).

Response to Comments of Reviewer #2

General Comment: The present paper describes a novel method for biomass valorisation using an integrated concept of photo-, electro- and biocatalysis. The concept is certainly very intriguing and the interest of the community should be high. However, there is one major conceptual flaw and several smaller issues which require rewriting major parts of the manuscript or drastically altering the claims. Therefore I recommend rejection of the article but encourage the authors to resubmit a thoroughly reworked version at a later point.

Response: Thank you very much for the reviewer's endorsement of our work and sound criticism. The reviewer raised major concerns on the use of BR buffer containing acetate. In the revision, we tried to address the reviewer's concerns fully by performing experiments in acetate-free conditions. Details of our response to the reviewer's concerns are delineated below.

Comment 1: Regarding the major conceptual flaw in the report. The authors claim to perform water oxidation in the (photo)anode cell, yet they use a BR buffer which contains significant amounts of acetate. It is well known that acetate may act as a sacrificial electron donor for TiO2 and thereby accelerate the reductive reaction. As such, it is not water oxidation that is performed but rather acetate oxidation. Analysis of acetate concentrations as well as the product and potential intermediates of its oxidation (formaldehyde, formate, carbon dioxide) should be performed to exclude this or the claim of water oxidation must be dropped.

Response: We truly appreciate pointing out this critical point which we missed. To rule out the potential effect of acetate as a sacrificial agent in water oxidation, we measured the photoelectrochemical water oxidation activity of H:TiO_2 in acetate-free phosphate borate solution (pH 4.5). We found that the turn-on voltage of photoanode was similar to that in the BR buffer at pH 4.5 (Review Only Fig. 4). We also checked the H_2O_2 generation activity of cathode catalysts in the acetate-free solution (Revised Fig. 2b). Based on the (photo)electrochemical activities of the photoanode and cathode, we confirm that our photoelectrochemical cell can operate without applied voltage under AM1.5G irradiation.
We have performed the main experiments again (lignin degradation and biopolymer synthesis) in the phosphate borate solution (w/o acetate) with higher enzyme concentration (Revised Fig. 3 and 4). The final conversion and polymer yield in the three-compartment system were similar to previous data (with BR buffer) and the overall trend was similar for each system, although the lignin conversion and polymer yield two-compartment were slightly higher than before. Therefore, the original claim that our three-compartment system can make unassisted lignin valorisation by bias-free water oxidation is still valid in the absence of acetate.

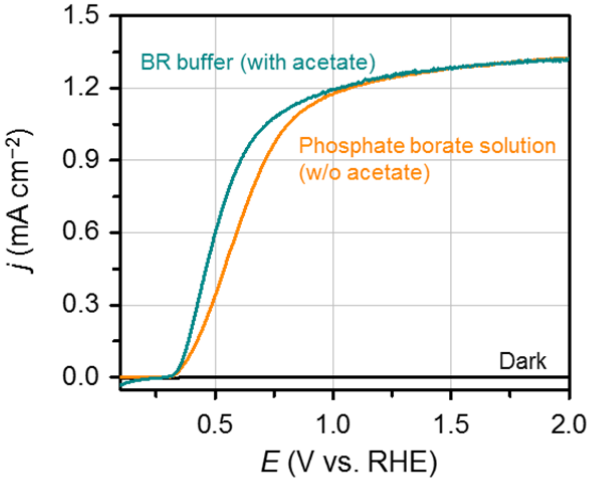

Review Only Fig. 4 | *I-V* curves of H:TiO$_2$ in 0.1 M BR buffer, pH 4.5 and in 0.1 M phosphate borate solution, pH 4.5 under simulated 1 sun (AM1.5G) illumination.

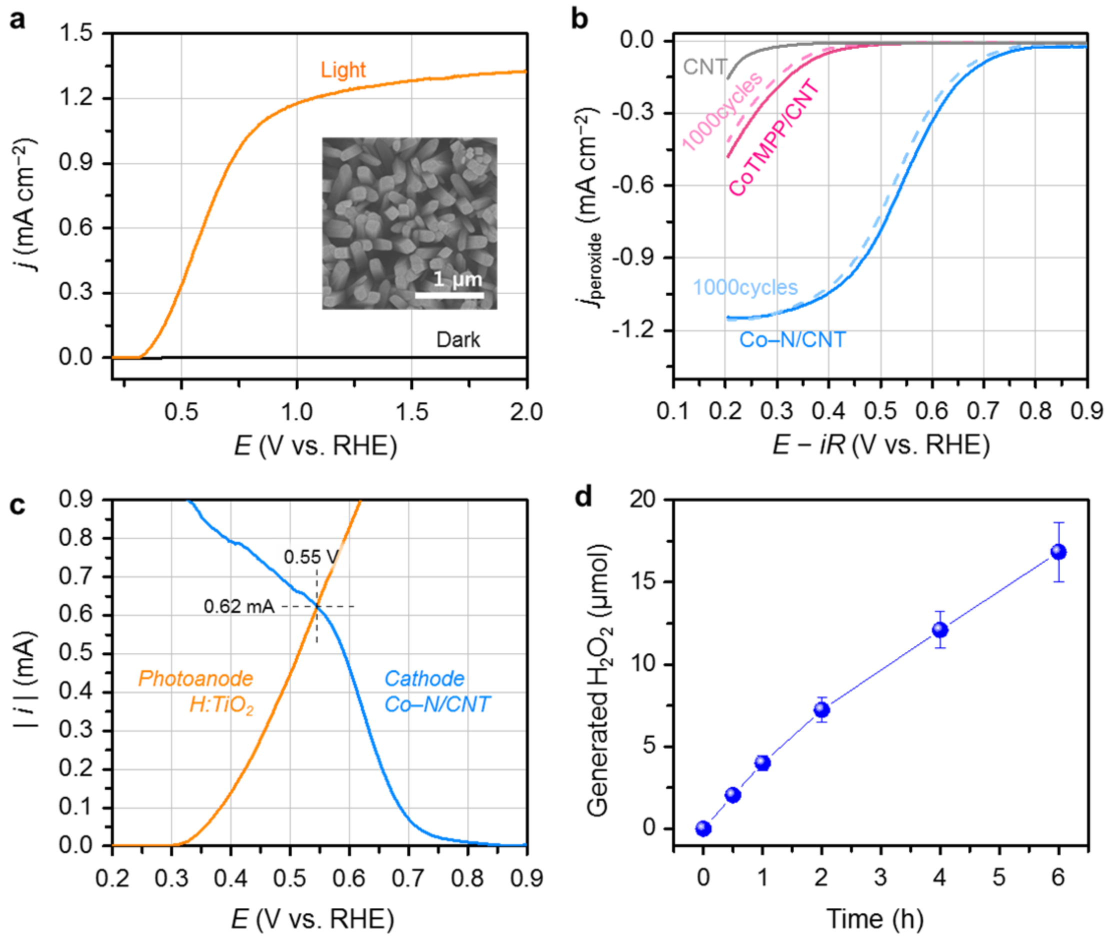

Revised Fig. 2 | Photo-electrocatalytic production of H$_2$O$_2$ in the integrated three-compartment reactor. a, Photocurrent density of the H:TiO$_2$ electrode under illumination and in the dark in 0.1 M phosphate borate solution at pH 4.5. Inset: SEM image of H:TiO$_2$. **b**,

Linear sweep voltammetry curves of Co–N/CNT, CoTMPP/CNT, and CNT catalysts before and after potential cycling tests. The current densities for H_2O_2 production are shown and were obtained via rotating ring disk electrode measurements at an electrode rotation speed of 1,600 rpm in O_2-saturated 0.1 M phosphate borate solution at pH 4.5. **c**, *I-V* curves of H:TiO_2 photoanode under simulated 1 sun (AM1.5G) illumination and 0.1 M phosphate borate solution at pH 4.5 and *I-V* curves of Co–N/CNT cathode in O_2-saturated 0.1 M phosphate borate solution at pH 4.5. **d**, Amount of H_2O_2 generated over time in 0.1 M phosphate borate solution at pH 4.5 during the operation of the integrated three-compartment reactor.

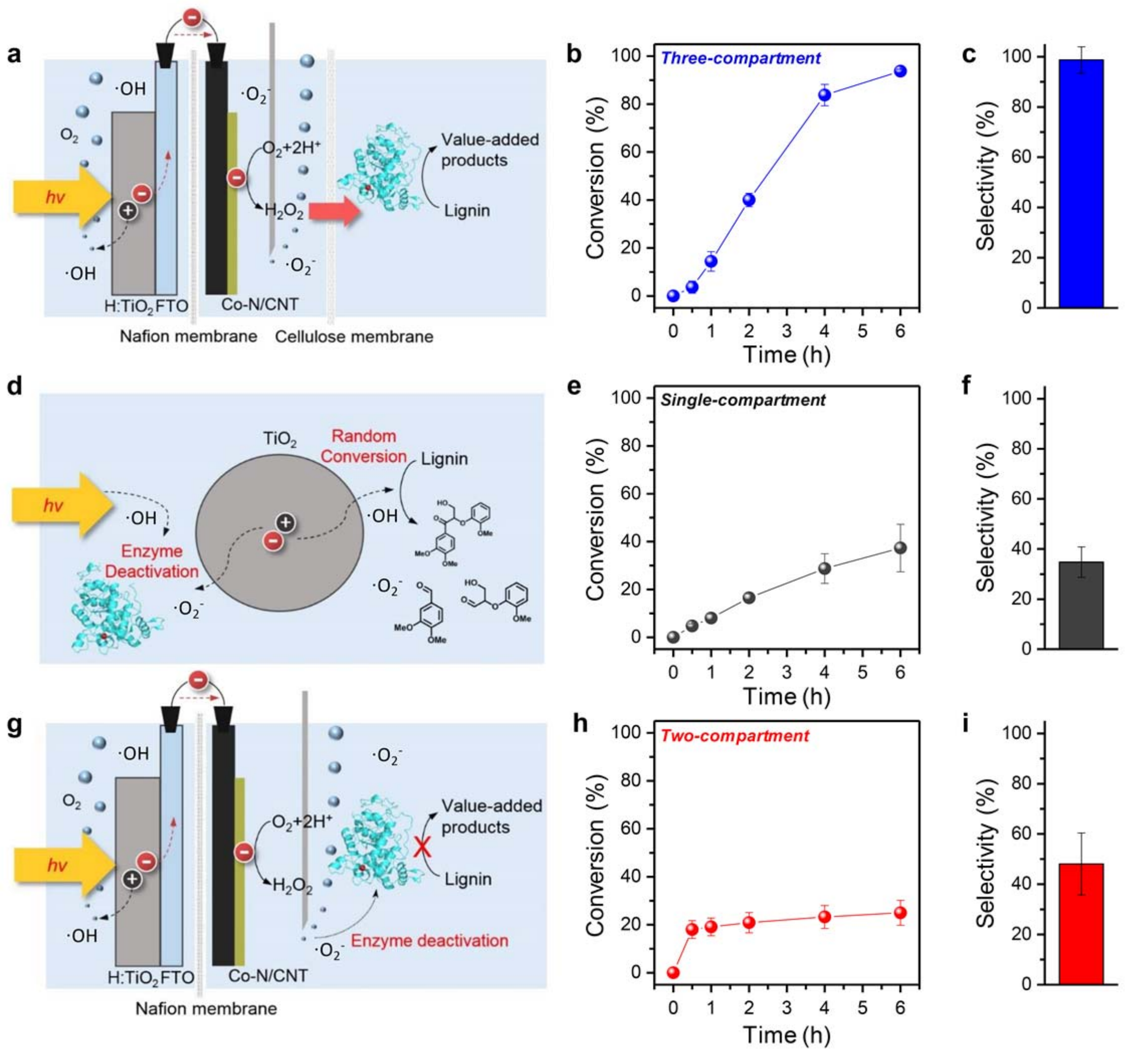

Revised Fig. 3 | Reactor configurations and photo-electro-biochemical lignin dimer conversion. a,**d**,**g**, Schematic representations of the three-compartment photo-electro-biochemical system (**a**), single-compartment powder-based bio-photochemical system (**d**), and two-compartment photo-electrochemical system (**g**) used for lignin dimer conversion. **b**,**e**,**h**, Conversion and **c**,**f**,**i**, selectivity of the three-compartment photo-electro-biochemical system (**b**,**c**), single-compartment powder-based bio-photochemical system (**e**,**f**), and two-compartment photo-electrochemical system (**h**,**i**).

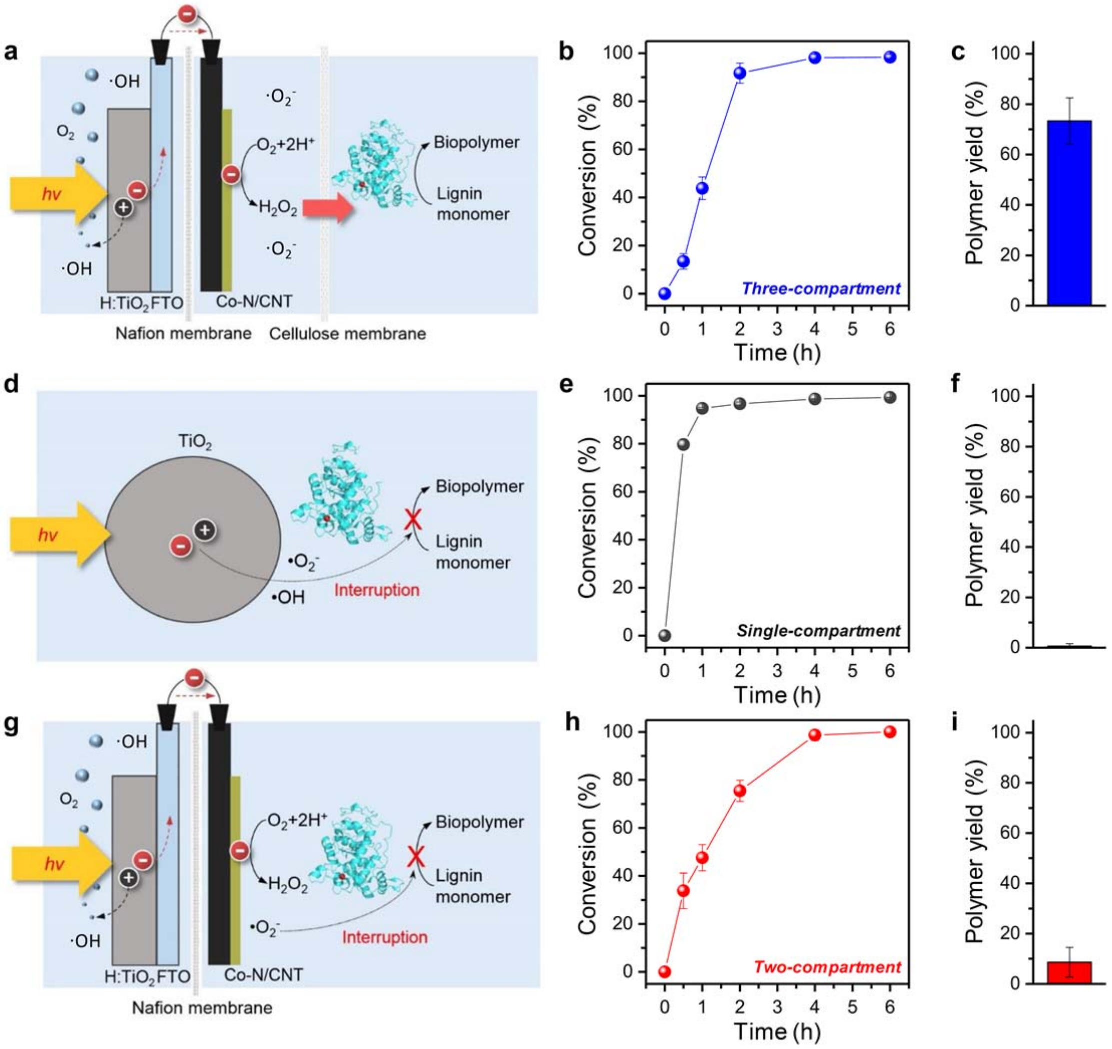

Revised Fig. 4 | Reactor configurations and photo-electro-biochemical synthesis of biopolymer from coniferyl alcohol. a,**d**,**g**, Schematic representations of the (**a**), three-compartment photo-electro-biochemical system, (**d**), single-compartment powder-based bio-photochemical system, and (**g**), two-compartment photo-electrochemical system used for biopolymer synthesis. **b**,**e**,**h**, Conversion percentage of coniferyl alcohol over time and **c**,**f**,**i**, polymer yield after 6 h of reaction of the three-compartment photo-electro-biochemical system (**b**,**c**), single-compartment powder-based bio-photochemical system (**e**,**f**), and two-compartment photo-electrochemical system (**h**,**i**).

As the solution changed, the ORR activity and selectivity of the catalysts (Revised Supplementary Fig. 8), the H_2O_2 production experiments at each system (Revised Supplementary Fig. 12, 17, and 23), enzyme stability tests (Revised Supplementary Fig. 14 and 34), the control reactions in the absence of enzyme for lignin degradation (Revised Supplementary Fig. 15, 16, and 24), and biopolymer synthesis (Revised Supplementary Fig. 35–37) were performed again in the phosphate borate solution. The overall trends are almost identical, with the only difference being H_2O_2 production in the single-compartment by TiO_2

powder-type photocatalyst. Bare TiO_2 powder cannot produce H_2O_2 in the absence of acetate (Revised Supplementary Fig. 17), which indicates that acetate will act as a sacrificial agent in the powder system. However, TiO_2 powder-type photocatalyst will still increase lignin random conversion by the generated charge carriers on TiO_2 surface (Revised Supplementary Fig. 16). Therefore, powder-type photocatalyst is challenging to be compatible with biocatalytic reactions, as before.

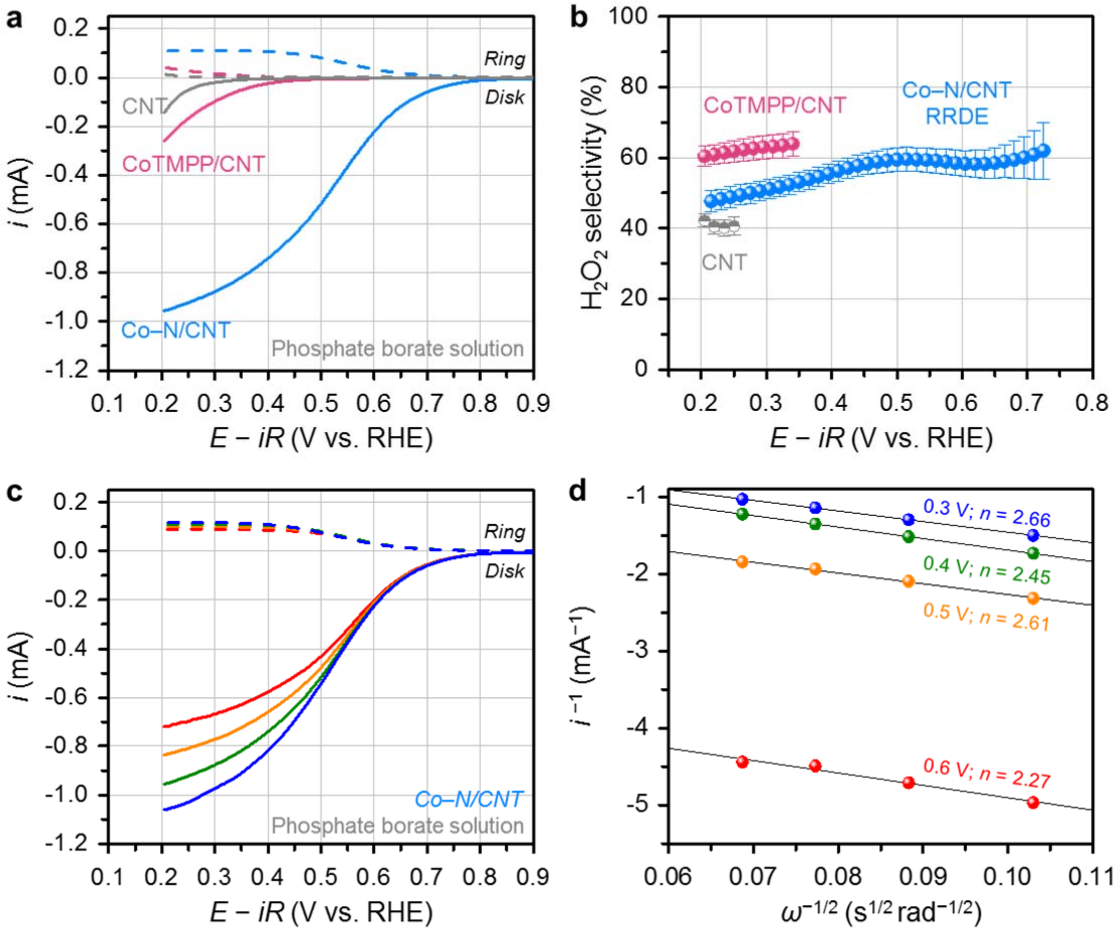

Revised Supplementary Fig. 8 | ORR Activity and Selectivity. a, ORR polarisation curves and **b**, H_2O_2 selectivity of Co–N/CNT, CoTMPP, and CNT measured in 0.1 M phosphate borate solution by the RRDE method. **c**, ORR polarisation curves of Co–N/CNT measured in 0.1 M phosphate borate solution, pH 4.5 at different electrode rotation speeds and **d**, corresponding K–L plots.

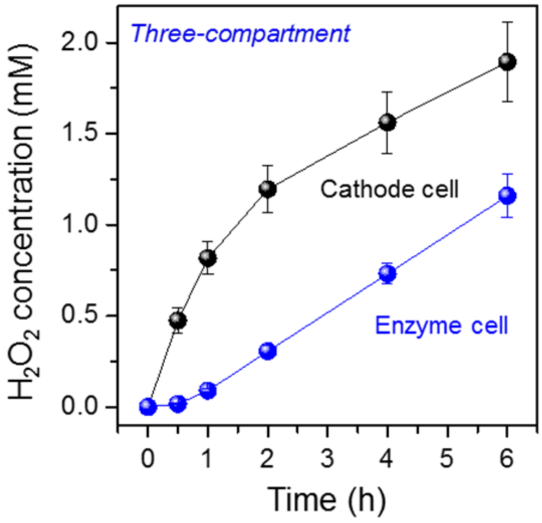

Revised Supplementary Fig. 12 | Photo-electrochemical H_2O_2 production using H:TiO_2/FTO photoanode and Co–N/CNT cathode in the three-compartment acryl reactor equipped with a quartz window. Changes in H_2O_2 concentration as a function of time in the cathode cell (4 mL) and enzyme cell (8 mL) under illumination. Experimental conditions: 0.1 M phosphate borate solution, pH 4.5, and solar simulator irradiation at 100 mW cm^{-2} (AM1.5G).

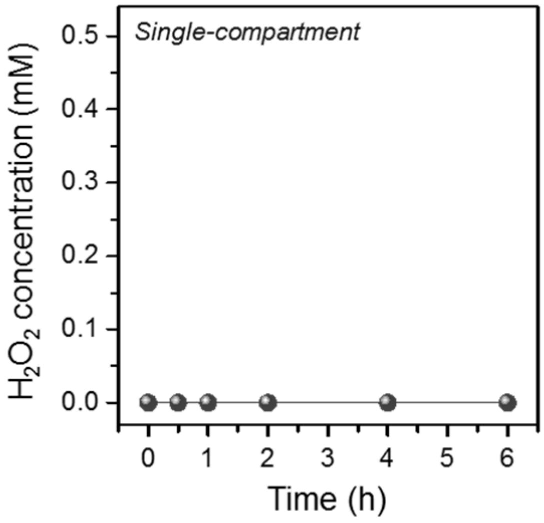

Revised Supplementary Fig. 17 | Photochemical H_2O_2 production in the single-compartment quartz reactor using TiO_2 powder. Changes in H_2O_2 concentration as a function of time under illumination. Experimental conditions: 0.1 M phosphate borate solution, pH 4.5, reaction volume 8 mL, catalyst concentration 0.5 mg mL^{-1}, and solar simulator irradiation at 100 mW cm^{-2} (AM1.5G).

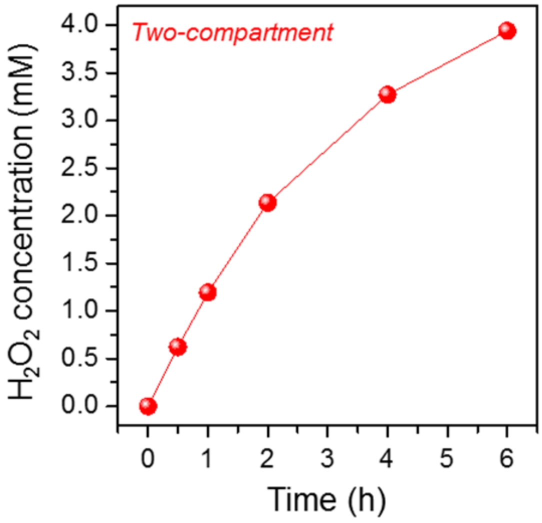

Revised Supplementary Fig. 23 | Photo-electrochemical H_2O_2 production using a H:TiO_2/FTO photoanode and Co–N/CNT cathode in the two-compartment acryl reactor equipped with a quartz window. Variation in the H_2O_2 concentration as a function of time in the cathodic compartment (4 mL) under illumination. Experimental conditions: 0.1 M phosphate borate solution, pH 4.5, and solar simulator irradiation at 100 mW cm^{-2} (AM1.5G).

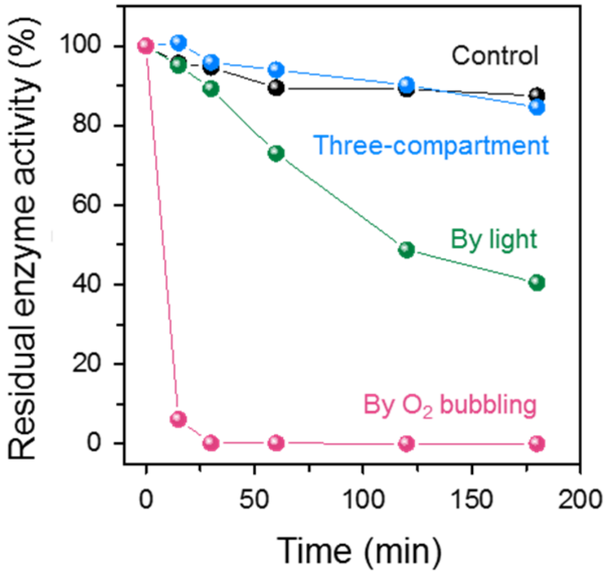

Revised Supplementary Fig. 14 | Enzyme LiPH8 stability test. Effect of O_2 bubbling and exposure to light irradiation on the activity of LiPH8. Residual enzyme activity after incubation with stirring in phosphate borate solution, pH 4.5: without light irradiation and O_2 purging (●), with light irradiation and without O_2 bubbling (●), and without light irradiation and with O_2 bubbling (●) in the two-compartment reactor. The blue circles (●) represent the variation in enzyme activity with light irradiation and O_2 purging in three-compartment reactor. All experiments were performed with constant stirring at room temperature.

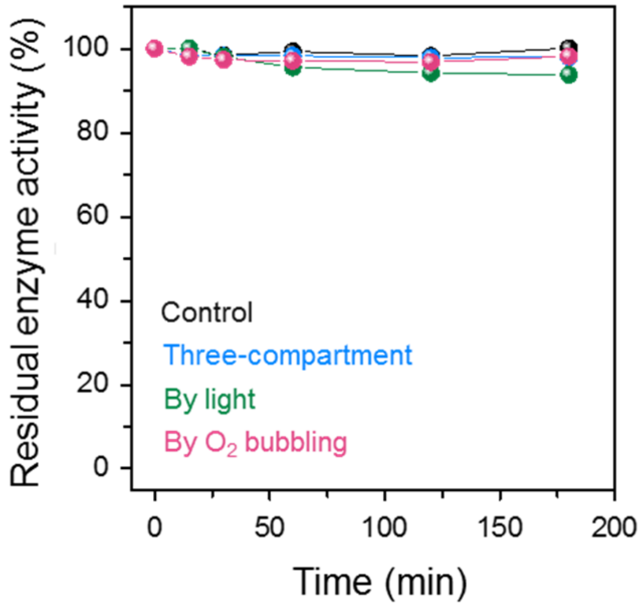

Revised Supplementary Fig. 34 | Enzyme HRP stability test. Effect of O_2 bubbling and exposure to light irradiation on HRP activity. Residual enzyme activity after incubation with stirring in phosphate borate solution, pH 6.0: without light irradiation and O_2 purging (●), with light irradiation and without O_2 bubbling (●), and without light irradiation and with O_2 bubbling (●) in the two-compartment reactor. The blue circles (●) represent the variation in enzyme activity with light irradiation and O_2 purging in the three-compartment reactor. All experiments were performed with stirring at room temperature.

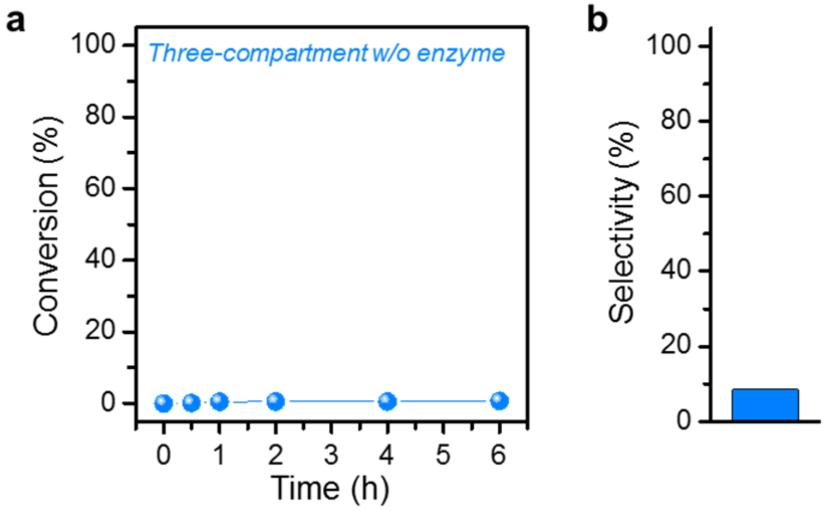

Revised Supplementary Fig. 15 | Photo-electrochemical lignin degradation (i.e. without enzyme) in the three-compartment acryl reactor equipped with a quartz window. a, Lignin conversion **and** b, selectivity. Experimental conditions: 0.1 M phosphate borate solution, pH 4.5, and solar simulator irradiation at 100 mW cm^{-2} (AM1.5G).

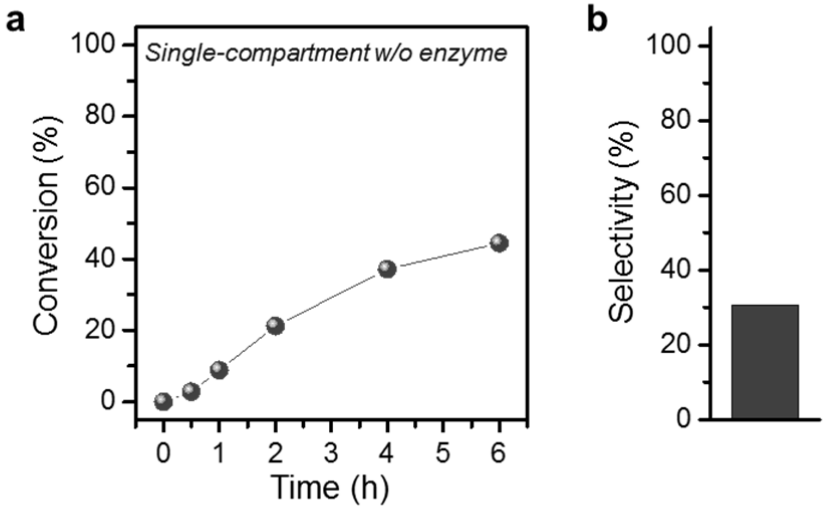

Revised Supplementary Fig. 16 | Photochemical lignin degradation (i.e. in the powder system without enzyme) in the single-compartment quartz reactor. a, Lignin conversion and **b**, selectivity. Experimental conditions: 0.1 M phosphate borate solution, pH 4.5, reaction volume 8 mL, catalyst concentration 0.5 mg mL^{-1}, and solar simulator irradiation at 100 mW cm^{-2} (AM1.5G).

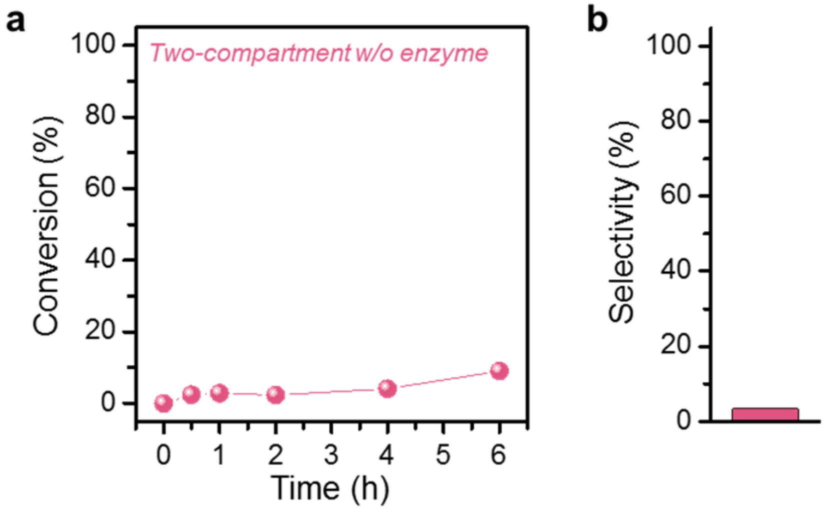

Revised Supplementary Fig. 24 | Photo-electrochemical lignin degradation (i.e. without enzyme) in the two-compartment acryl reactor equipped with a quartz window. a, Lignin conversion and **b**, selectivity. Experimental conditions: 0.1 M phosphate borate solution, pH 4.5, and solar simulator irradiation at 100 mW cm^{-2} (AM1.5G).

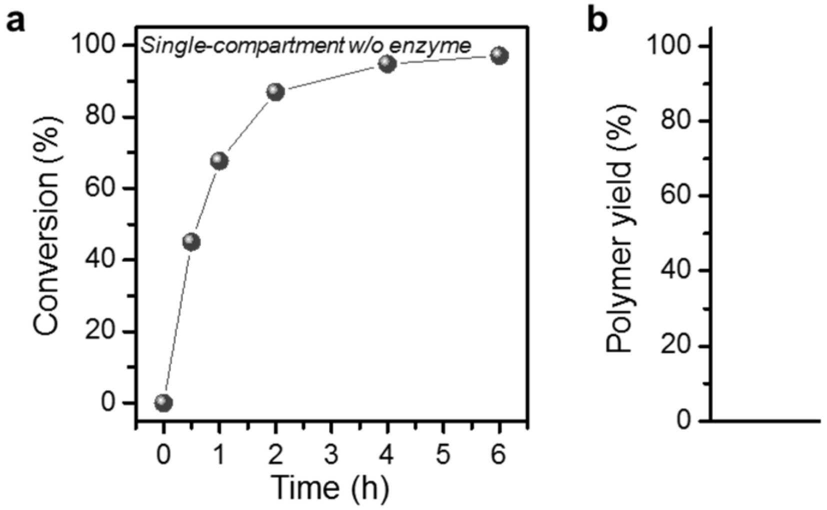

Revised Supplementary Fig. 35 | Photochemical biopolymer synthesis (i.e. the powder system without enzyme) in the single-compartment quartz reactor. a, Conversion and **b**, polymer yield. Experimental conditions: 0.1 M phosphate borate solution, pH 6.0, reaction volume 8 mL, catalyst concentration 0.5 mg mL^{-1}, and solar simulator irradiation at 100 mW cm^{-2} (AM1.5G).

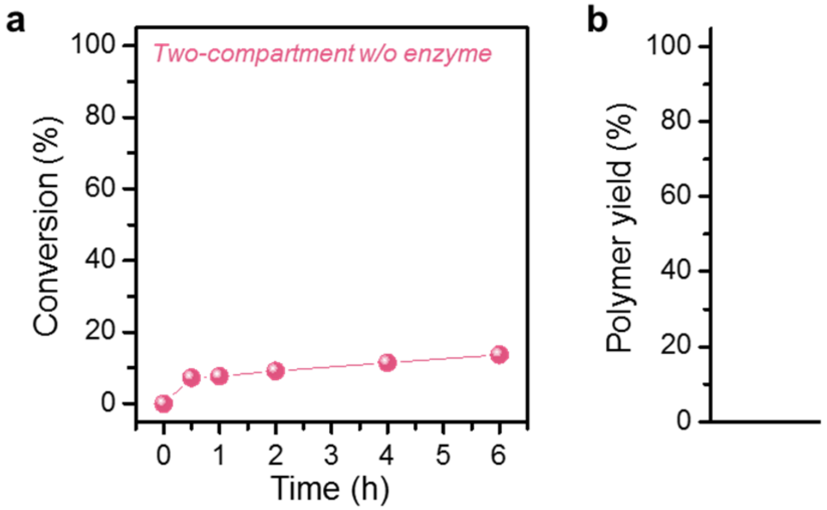

Revised Supplementary Fig. 36 | Photo-electrochemical biopolymer synthesis (i.e. without enzyme) in the two-compartment acryl reactor equipped with a quartz window. a, Conversion and **b**, polymer yield. Experimental conditions: 0.1 M phosphate borate solution, pH 6.0, and solar simulator irradiation at 100 mW cm^{-2} (AM1.5G).

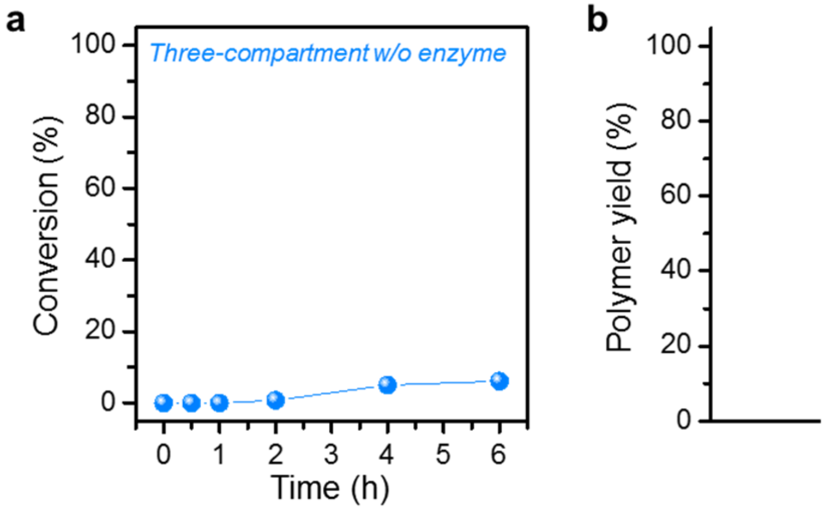

Revised Supplementary Fig. 37 | Photo-electrochemical biopolymer synthesis (i.e. without enzyme) in the three-compartment acryl reactor equipped with a quartz window. **a**, Conversion and **b**, polymer yield. Experimental conditions: 0.1 M phosphate borate solution, pH 6.0, and solar simulator irradiation at 100 mW cm^{-2} (AM1.5G).

Comment 2: Furthermore, the paper should give more information on the overall efficiency of the system, e.g., how much of the H2O2 generated is converted by the enzyme to the desired product and what is the fate of the remainder. How high is the TTN and turnover rate of the enzymes and how does that compare to other reported values for these enzymes, i.e., how efficiently are they utilized in the present system.

Response: We appreciate this valuable comment. We calculated i) the amount of H_2O_2 consumed by the enzyme to yield the desired product and ii) turnover frequency (TOF) of the enzyme.

i) The amount of H_2O_2 consumed by the enzyme and the proportion of the consumed to the generated amount of H_2O_2

1. The amount of lignin dimer converted to the desired product

$$= (0.5\ \text{mM lignin dimer}) \times (8\ \text{mL}) \times (93.73\%\ \text{Conv.}) \times (98.75\%\ \text{selec.})$$
$$= 3.62\ \mu\text{mol lignin dimer}$$

2. The amount of H_2O_2 generated in the three-compartment system for 6 h (shown in Fig. 2d)

$$= 16.84\ \mu\text{mol}\ H_2O_2\ \text{at 6 h}$$

3. The amount of H_2O_2 in the enzyme compartment at 6 h of operation (Supplementary Fig. 9)

$$= 9.27\ \mu\text{mol}\ H_2O_2\ \text{in the enzyme compartment}$$

4. The proportion of the consumed amount of H_2O_2 in the whole 3-compartment for lignin dimer bond-cleavage

$$= (3.62\ \mu\text{mol lignin dimer}) \times \frac{100}{15.3\ \mu\text{mol}\ H_2O_2} = 21.48\%$$

5. The proportion of the consumed amount of H_2O_2 in the enzyme compartment for lignin

dimer bond-cleavage

$$= (3.62\ \mu\text{mol lignin dimer}) \times \frac{100}{9.27\ \mu\text{mol } H_2O_2} = 39.0\%$$

The above calculations show that 39% of the H_2O_2 is consumed when it is moved to the enzyme compartment from the cathode department. As the concentration in the enzyme part is lowered by consumption due to lignin dimer conversion, higher concentrations of H_2O_2 in the cathode part are maintained, resulting in a continuous transfer of H_2O_2 from the cathode to the enzyme compartment.

ii) Average TOF of the enzyme: 0.036 s^{-1} (Activity was recorded after 4 h)

$$\text{TOF} = \frac{(\text{Converted lignin dimer})}{(\text{The amount of enzyme}) \times (\text{Time})}$$

**Ref**	**Lignin peroxidase (LiP)**	**Substrate**	**TOF (s^{-1})**
This work	LiPH8 from *P. chrysosporium*	Non-phenolic dimer	0.036 (at pH 4.5)<br>at 4 h of operation
Biotechnol. Biofuels **9**, 247 (2016)	LiPH8 from *P. chrysosporium*	Non-phenolic dimer<br>(Steady-state kinetic)	1.0210 (at pH 2.6)<br>0.7791 (at pH 3.0)<br>0.2031 (at pH 3.8)

We calculated the average TOF of the LiPH8 enzyme in the three-compartment reactor according to the above equation. The calculated TOF of LiPH8 enzyme used in this system is 0.036 s^{-1}. We compared this TOF value to those of reported lignin peroxidase (LiPH8). The TOF of the LiPH8 enzyme in our system is relatively lower than that of the other reported LiPH8 enzyme (~0.2 s^{-1} at pH 3.8) for *in vitro* systems. The reason for the relatively lower TOF in our system was found that the number of selective conversions of lignin dimer to monomer products is dependent on the H_2O_2 concentration present in the enzyme cell (Supplementary Fig. 29). The TOF increased as the diffusion rate of H_2O_2 from cathode cell to enzyme cell increased and reached a maximum at 4 h. At 6 h, TOF decreased because almost all lignin dimer was converted before 4 h. The serious detrimental effect of excess H_2O_2 on lignin peroxidase has been reported in many previous works [*Arch. Biochem. Biophys.* **316**, 851 (1995); *Enzyme Microb. Technol.* **61–62**, 48 (2014); *Biotechnol. Biofuels* 9, 247 (2016)]. However, in our system, the enzyme could maintain activity up to 6 h of operation. Besides preventing the unselective catalysis of Vis/UV irritation, the stability of the enzyme in this system is also advantageous compared with other reported reactors for its higher productivity in lignin conversion. It is suggested that the optimal diffusion rate through the membrane cathode cell to the enzyme cell gradually provided H_2O_2 to the enzyme, resulting in a balanced oxidation-reduction catalytic cycle.

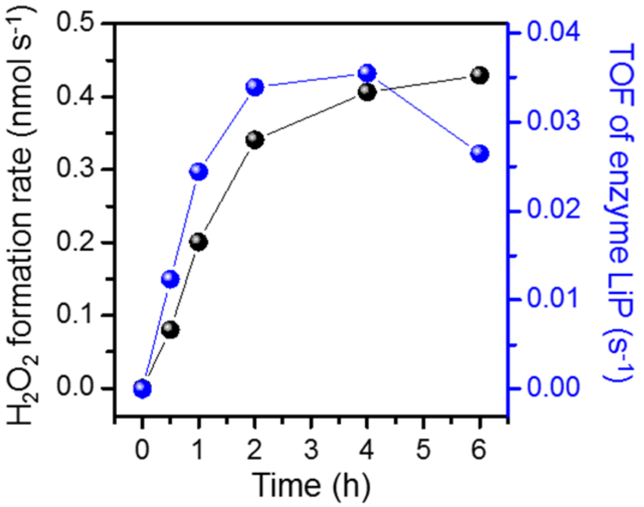

Supplementary Fig. 29 | Correlation between H_2O_2 formation rate and TOF of enzyme LiP. H_2O_2 formation rate in the enzyme cell (●) and turnover frequency of enzyme LiP (●).

Added contents in the revised manuscript text, Page no. 18

"Turnover frequency (TOF) of the enzyme was 0.036 $^{s-1}$, which is relatively lower than that of the LiPH8 enzyme reported in the literature[48], due to the rate of lignin conversion being limited by H_2O_2 concentration (Supplementary Fig. 29). These results show that TOF can be controlled in a stable manner by adjusting the H_2O_2 generation speed of the photo-electro-biochemical system."

Added reference in the revised manuscript

48 Pham, L. T. M., Kim, S. J. & Kim, Y. H. Improvement of catalytic performance of lignin peroxidase for the enhanced degradation of lignocellulose biomass based on the imbedded electron-relay in long-range electron transfer route. *Biotechnol. Biofuels* **9**, 247 (2016).

We applied this three-compartment system for polymerisation under catalysts of enzyme horseradish peroxidase (HRP) at pH 6.0. Formation rate of H_2O_2 at pH 6.0 is comparable with that at pH 4.5, and the yield of polymer in our study (73.3%) is also comparable with previously reported studies for *in vitro* polymerisation, as shown in the table below.

Ref	Horseradish peroxidase (HRP)	Substrate	Polymer yield (%)
This work	HRP (Sigma)	Coniferyl alcohol	73.3% (at pH 6.0)
Chem. Pharm. Bull. **39**, 950 (1991)	HRP (Sigma)	Coniferyl alcohol	90.1% (at pH 6.0)

Biotechnol. Prog. **34**, 81 (2018)	Versatile peroxidase from *Bjerkandera sp.*	Coniferyl alcohol	99.3 ± 1.2% (pH 6) with Mn^{2+} mediator

Comment 3: How does the efficiency compare to just adding a (dilute) H_2O_2 solution to the lignin/enzyme solution.

Response: This is a very intriguing comment that suggests the comparison of the system in which H_2O_2 is added at the beginning with our continuous H_2O_2 supply system. Following the reviewer's suggestion, we performed lignin degradation experiments by simply adding dilute H_2O_2 to the lignin/enzyme solution. The obtained lignin dimer conversions were 82% and 21% when the concentrations of H_2O_2 were 1 and 10 mM, respectively (Supplementary Fig. 27). Although the enzymatic reaction was very fast under these conditions (conversion completed within 30 min), the final conversions (82% for 1 mM and 21% for 10 mM) were lower than those of the three-compartment H_2O_2 supply system (94%) because of enzyme deactivation due to high H_2O_2 concentration. To confirm the importance of continuous supply of a low concentration of H_2O_2 to the enzyme, we conducted lignin degradation while feeding small amounts of H_2O_2 every 15 minutes. The continuous addition of H_2O_2 resulted in a higher final conversion than when H_2O_2 was added at once at the beginning (Supplementary Fig. 28). These results clearly indicate that the continuous and low concentration of H_2O_2 supply is preferred for the efficient biocatalytic lignin degradation. Therefore, this compartmented photo-electro-biochemical system is a more efficient approach than direct H_2O_2 addition system.

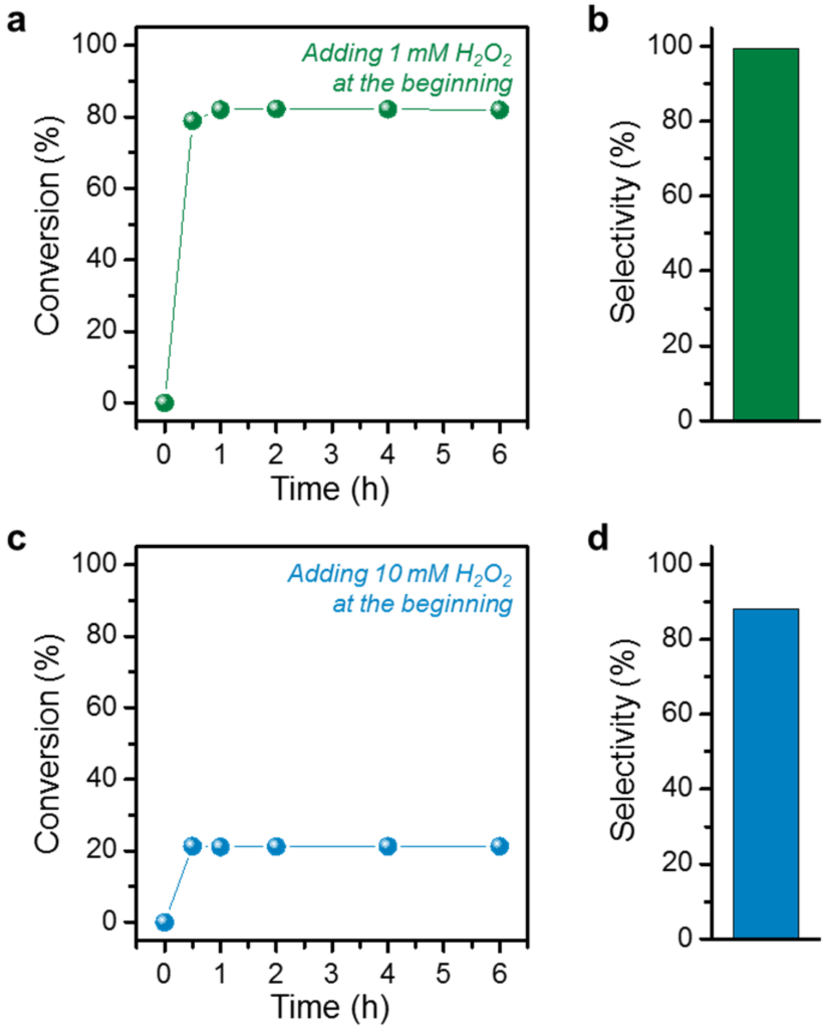

Supplementary Fig. 27 | Biocatalytic lignin degradation with H_2O_2 addition at the beginning. **a**,**c**, Lignin conversion and **b**,**d**, selectivity at 1 mM (**a**,**b**) and 10 mM (**c**,**d**) H_2O_2 concentration. Experimental conditions: 0.1 M phosphate borate solution, pH 4.5, 0.5 mM of lignin dimer, 0.8 µM of the LiPH8 enzyme, and adding 1 or 10 mM H_2O_2 at the beginning of the reaction.

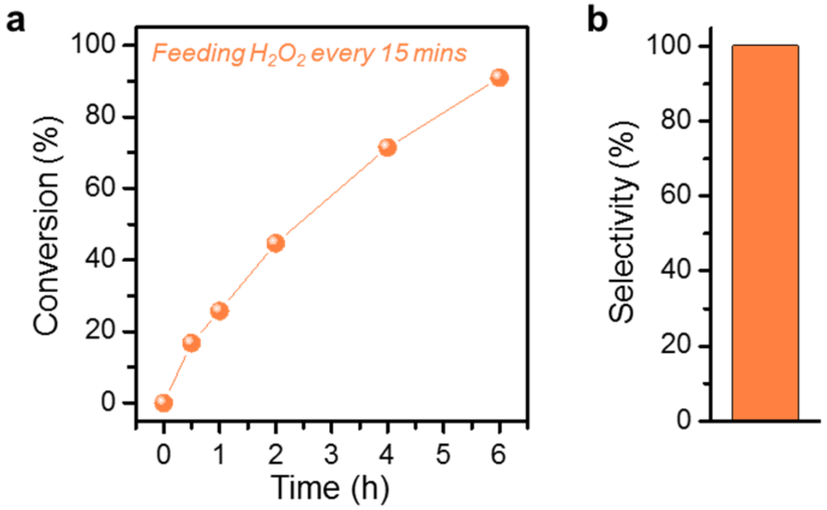

Supplementary Fig. 28 | Biocatalytic lignin degradation with continuous H_2O_2 feeding. a, Lignin conversion and **b**, selectivity. Experimental conditions: 0.1 M phosphate borate solution, pH 4.5, 0.5 mM of lignin dimer, 0.8 μM of the LiPH8 enzyme, and feeding H_2O_2 every 15 mins (total 1 mM).

Added contents in the revised manuscript text, Page no. 17−18

"The gradual increase of H_2O_2 concentration in the three-compartment cell is also a reason for the high performance, as this helps to maintain low concentrations of H_2O_2 in the enzyme cell, as shown. When the initial concentrations were 1 mM and 10 mM, the conversion efficiencies were 82% and 21%, respectively. These values are lower than that of three-compartment system (93.7%) because of enzyme deactivation due to high H_2O_2 concentration (Supplementary Fig. 27). When we fed a small amount of H_2O_2 every 15 mins, the conversion efficiency was increased, indicating that the continuous and low concentration of H_2O_2 supply is preferred for the efficient biocatalytic lignin degradation (Supplementary Fig. 28)."

Comment 4: The RRDE experiments indicate that oxygen is reduced in a two-electron reduction which directly yields hydrogen peroxide (in contrast to the one-electron reduction yielding superoxide). Yet the authors blame superoxide/hydroperoxide radicals for some of the observed enzyme deactivation and side-reactions. The authors should experimentally determine superoxide to clarify this.

Response: We thank the reviewer for their valuable criticism. Our claim regarding the superoxide radical is based on the current understanding of the reaction mechanism for the electrochemical reduction of O_2. Superoxide appears at the first step of oxygen reduction by a single electron transfer to the O_2 molecule ($O_2 + e^- \rightarrow {\bullet}O_2^-$). As a response to the reviewer's

comment, we tried to detect the superoxide radical (•O_2^-) at the cathode and hydroxyl radical (•OH) at the photoanode.

The detection of the radicals was conducted using a fluorescence probe method with coumarin and the colouration method with XTT for •OH and •O_2^- radicals, respectively. To verify whether the generated radicals diffused into the adjacent cell, we checked for spectrum changes in the enzyme cell as well as the photoanode and cathode cells for •OH and •O_2^- radicals, respectively. The changes in fluorescence spectra at 455 nm in anode cell and enzyme cell (Supplementary Fig. 25) indicate that •OH was formed in the anode cell while the enzyme cell was free from the interruption of the hydroxyl radicals. Regarding superoxide radical detection, we used coloration of XTT reduction to XTT–formazam by superoxide anion because of the various restrictions of other methods, although there is a controversy that the coloration reagent could be reduced by the photoinduced electrons. There was no formation of XTT–formazam, which was monitored by the absorbance of the solution at 470 nm in the enzyme cell while cathode solution showed an increased absorbance at 470 nm (Supplementary Fig. 26). Although it is unclear whether XTT reduction was by superoxide anion or photoinduced electrons at the cathode, at least superoxide anion and reductive power (electrons) did not exist in the enzyme cell so that selective polymerisation could occur in the enzyme cell.

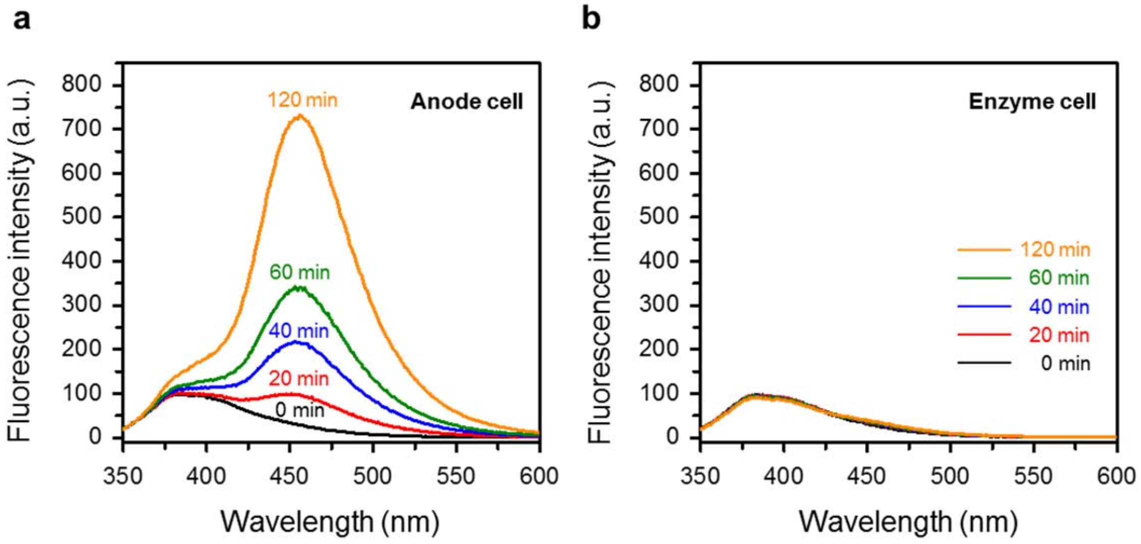

Supplementary Fig. 25 | Time-dependent fluorescence spectra of 0.2 mM coumarin solution. a, Anode cell and **b**, enzyme cell during unassisted H_2O_2 production.

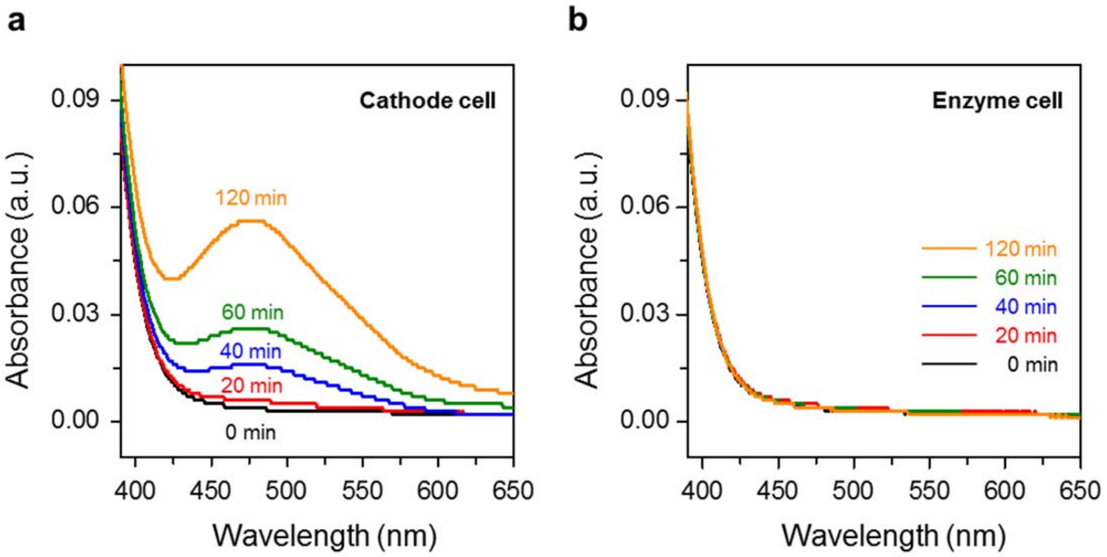

Supplementary Fig. 26 | Time-dependent UV-vis spectra of 0.1 mM XTT solution. a, Cathode cell and **b**, enzyme cell during unassisted H_2O_2 production.

Added contents in the revised manuscript text, Page no. 17

~~*The newly designed three-compartment photo-electro-biochemical system has distinct advantages over both single- and two-compartment systems, resulting in high conversion and selectivity of lignin dimer depolymerisation.*~~

→ *In the newly designed three-compartment photo-electro-biochemical system, we verified that the concentrations of •OH and •O_2^- radicals were very low in the enzyme cell compared to those in the anode and cathode cells, respectively (Supplementary Fig. 25,26), resulting in high conversion and selectivity of lignin dimer depolymerisation.*

Comment 5: For the experiments with coniferyl alcohol yielding many by-products it would be beneficial if the authors attempted to identify at least the major peaks in their chromatograms in order to give an idea of the possible mechanisms taking place.

Response: The precipitates from reactors were identified as biopolymer, a product from radical coupling of coniferyl alcohol. Firstly, this polymer was comprised of the common linkages between coniferyl alcohol molecules such as β-O-4, β-β and β-5 which were confirmed by two-dimensional-nuclear magnetic resonance spectroscopy (2D-NMR, Supplementary Fig. 31). We revised the relevant contents as followed:

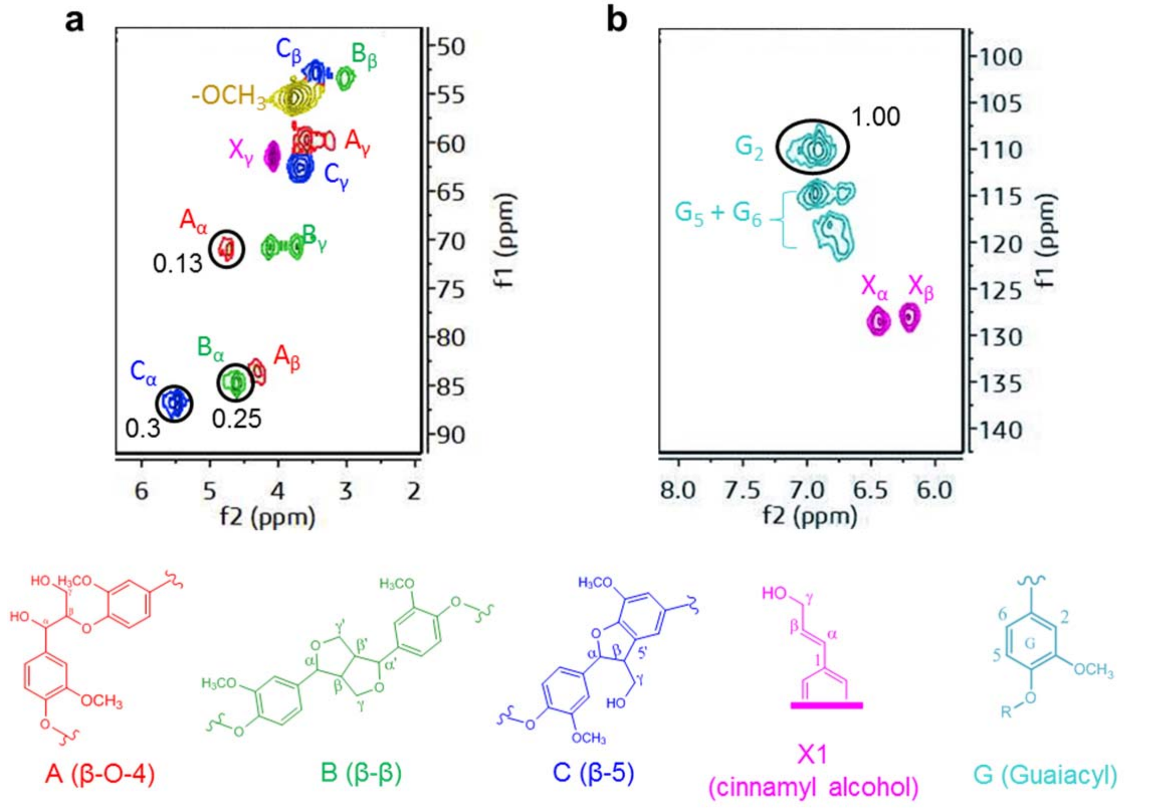

c

The major linkage types	Signal integral intensity	Content (%)
β-O-4	0.13	13.00
β-β	0.25	12.50
β-5	0.30	30.00

Supplementary Fig. 31 | Two-dimensional-nuclear magnetic resonance spectroscopy (2D-NMR) of the synthesized polymer in the three-compartmented system. a, Aliphatic region and **b**, aromatic region of 2D ^{13}C-^{1}H HSQC spectra from synthesized polymer in the three-compartment system in DMSO-d_6 and **c**, the contents of major linkages relative to the total aromatic units. Contours are colour coded according to the structures shown at the bottom of the figure. Relative signal integrations of the linkages to the aromatic region determined the content of the linkages in polymer structure. G_2 signal was used for G unit. Signal integrations of linkages were estimated from their C_α-H_α correlation, where the signal integration for β-β was logically halved to calculate β-β content.

Manuscript text, Page no. 18

"…, ~~and there were no detectable soluble by-products (Fig. 4c)~~. ~~Gel permeation chromatography (GPC) analysis revealed that the polymer product had a number-average molecular weight (M_n) of 1,330. "~~

→ "This polymer was comprised of the common linkages between coniferyl alcohol molecules such as β-O-4, β-β and β-5, and has a number average molecular weight of 1,103, which were confirmed by two-dimensional-nuclear magnetic resonance spectroscopy (2D-NMR, Supplementary Fig. 31), and gel permeation chromatography respectively."

Furthermore, through GC-MS analysis of the extracted soluble fractions in the post-reaction media, we could identify some by-products (23 min peak was not fully identified yet. Supplementary Fig. 32). Some monomeric by-products were found on the GC-MS chromatograms of single- and two-compartment systems (Supplementary Fig. 33). On the basis of these GC-MS analyses, reaction schemes for the pathways of coniferyl alcohol in different reactor systems are suggested in the revised Supplementary Fig. 30.

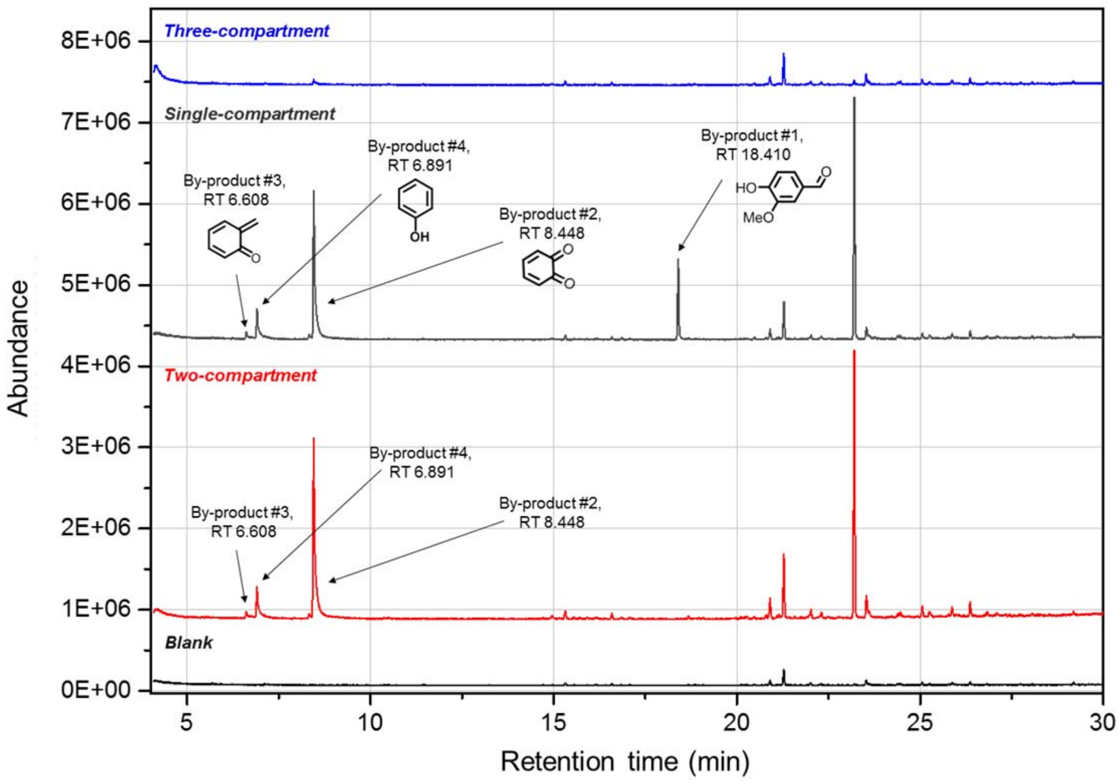

Supplementary Fig. 32 | GC-MS analysis of products from photo-electro-biochemical polymerization. GC-MS chromatograms of soluble fractions from reaction media of three, single, and two-compartment systems.

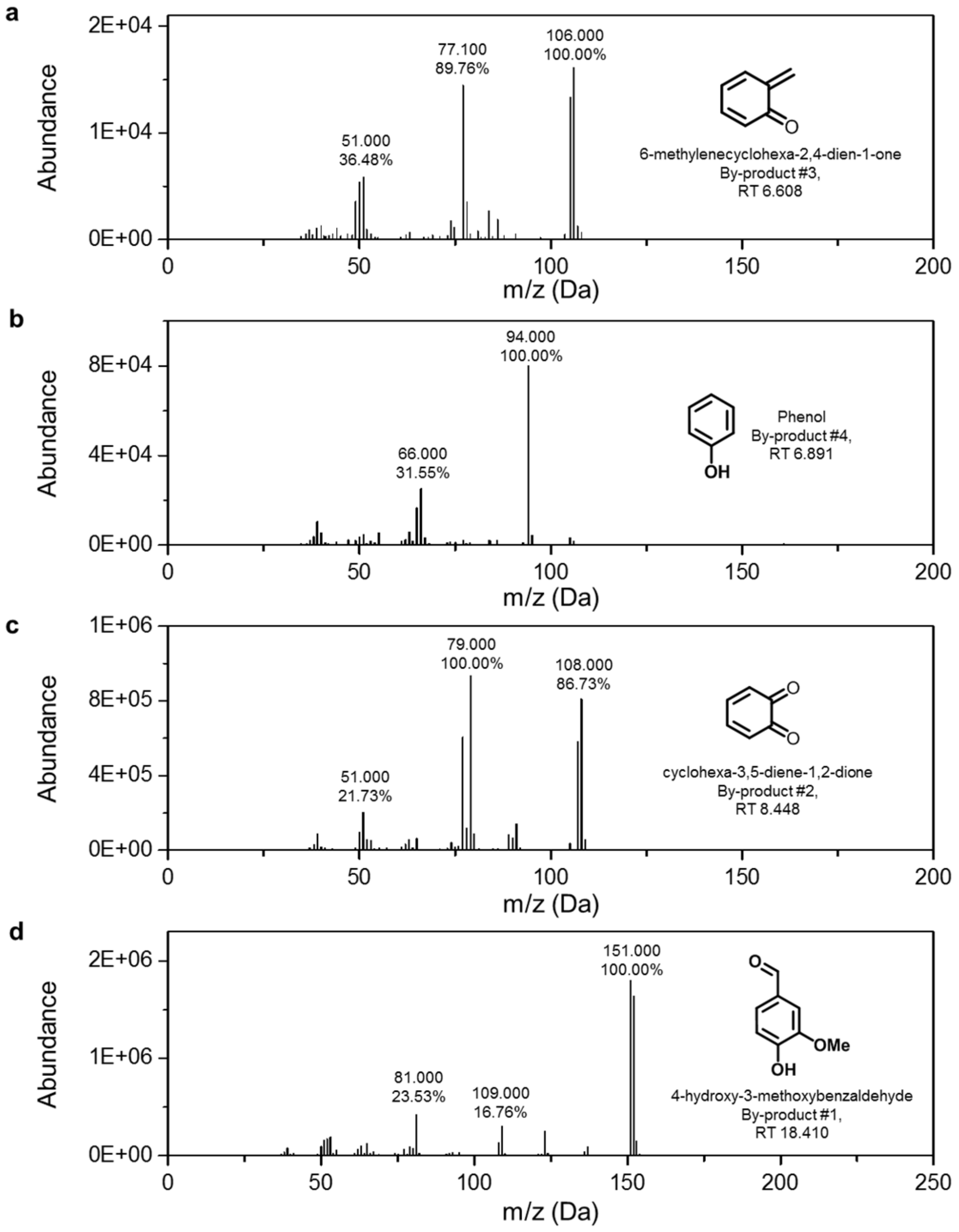

Supplementary Fig. 33 | Mass spectra of the substrate and products identified during photo-electro-biochemical biopolymer synthesis. a, By-products #3 **b**, By-products #4 **c**, By-products #2 **d**, By-products #1.

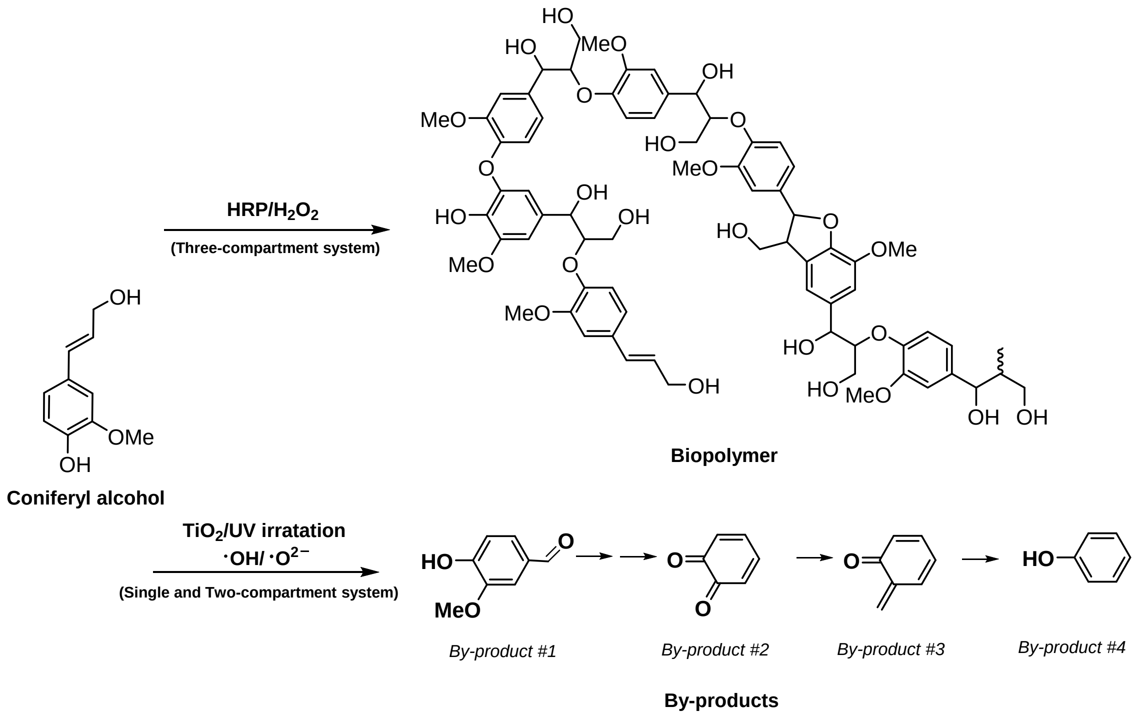

Revised Supplementary Fig. 30 | Reaction schemes for the pathways of coniferyl alcohol in different reactor systems.

Comment 6. Lastly, the authors should mention that many other groups have successfully integrated photocatalytic H2O2 generation with enzymes (e.g. peroxydases and peroxigenases).

Response

We thank the reviewer for improvement of our manuscript. Following the reviewer's comment, we have added recently reported important references regarding integrated photocatalytic H_2O_2 generation with enzymes in the revised manuscript.

References

32 Zhang, W. *et al.* Selective activation of C−H bonds in a cascade process combining photochemistry and biocatalysis. *Angew. Chem., Int. Ed.* **56**, 15451-15455 (2017).

33 Zhang, W. *et al.* Selective aerobic oxidation reactions using a combination of photocatalytic water oxidation and enzymatic oxyfunctionalizations. *Nat. Catal.* **1**, 55-62 (2018).

34 van Schie, M. M. C. H. *et al.* Cascading g-C_3N_4 and peroxygenases for selective oxyfunctionalization reactions. *ACS Catal.* **9**, 7409-7417 (2019).

35 Willot, S. J. P. *et al.* Expanding the spectrum of light-driven peroxygenase reactions. *ACS Catal.* **9**, 890-894 (2019).

Manuscript text, Page no. 4-5

Recently, several groups have combined this photocatalytic H_2O_2 system with biocatalyst and applied them for various chemical reactions. For example, a modified powder-type TiO_2 photocatalyst was utilised for H_2O_2 generation and it was integrated with a biocatalytic system of peroxygenases for the selective oxyfunctionalisations of carbon-hydrogen bonds, which has been one of the major challenges in organic synthesis[32-35].

Response to Comments of Reviewer #3

Comment 1: In the manuscript „Unassisted solar lignin valorisation using a compartmented photo-electro-biochemical cell" the author claimed that water was oxidized in the BR-buffer. The Britton-Robinson buffer contains different components, which could act as sacrificial agents. Before the acceptance of the paper could be taken into consideration the authors must investigate if really an unassisted water oxidation takes place.

Response: This a critical point we missed. Among components in BR buffer, acetate can act as a sacrificial agent and must be removed from the solution for water oxidation. To rule out the potential effect of acetate as a sacrificial agent in water oxidation, we measured the photoelectrochemical water oxidation activity of H:TiO_2 in acetate-free phosphate borate solution (pH 4.5). We found that the turn-on voltage of photoanode in the phosphate borate solution (w/o acetate) at pH 4.5 was similar to that of BR buffer at pH 4.5 (Review Only Fig. 4). We also checked the H_2O_2 generation activity of cathode catalysts in the acetate-free solution (Revised Fig. 2b). Based on the (photo)electrochemical activities of the photoanode and cathode, we confirm that our photoelectrochemical cell can operate without applied voltage under AM1.5G irradiation.

We have performed the main experiment again (lignin degradation, biopolymer synthesis) in a phosphate borate solution (w/o acetate) with higher enzyme concentration (Revised Fig. 3 and 4). The final conversion and polymer yield in the three-compartment system were similar to previous data (with BR buffer) and the overall trend was similar at each system, although the two-compartment lignin conversion and polymer yield were slightly higher than before. Therefore, the original claim that our three-compartment system can make unassisted lignin valorisation by bias-free water oxidation is still valid in the absence of acetate.

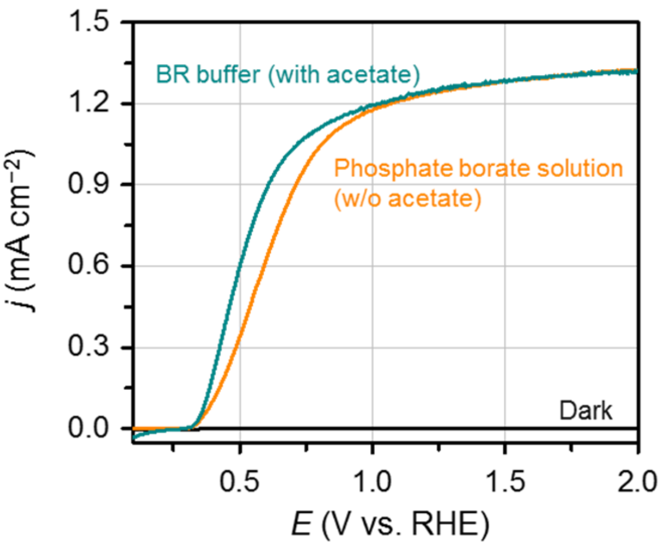

Review Only Fig. 4 | *I-V* curves of H:TiO_2 in 0.1 M BR buffer, pH 4.5 and in 0.1 M phosphate borate solution, pH 4.5 under simulated 1 sun (AM1.5G) illumination.

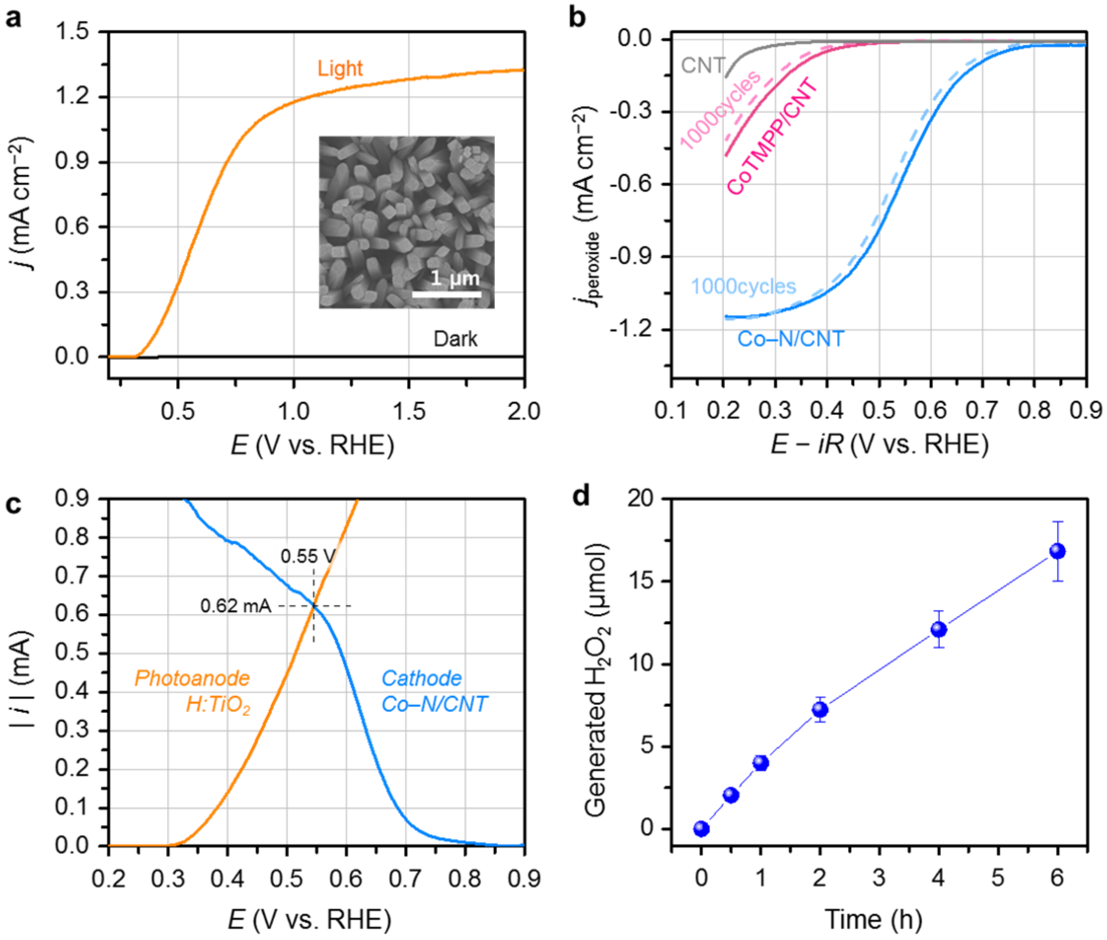

Revised Fig. 2 | Photo-electrocatalytic production of H_2O_2 in the integrated three-compartment reactor. a, Photocurrent density of the H:TiO_2 electrode under illumination and in the dark in 0.1 M phosphate borate solution at pH 4.5. Inset: SEM image of H:TiO_2. **b**, Linear sweep voltammetry curves of Co–N/CNT, CoTMPP/CNT, and CNT catalysts before and after potential cycling tests. The current densities for H_2O_2 production are shown and were obtained via rotating ring disk electrode measurements at an electrode rotation speed of 1,600 rpm in O_2-saturated 0.1 M phosphate borate solution at pH 4.5. **c**, *I-V* curves of H:TiO_2 photoanode under simulated 1 sun (AM1.5G) illumination and 0.1 M phosphate borate solution at pH 4.5 and *I-V* curves of Co–N/CNT cathode in O_2-saturated 0.1 M phosphate borate solution at pH 4.5. **d**, Amount of H_2O_2 generated over time in 0.1 M phosphate borate solution at pH 4.5 during the operation of the integrated three-compartment reactor

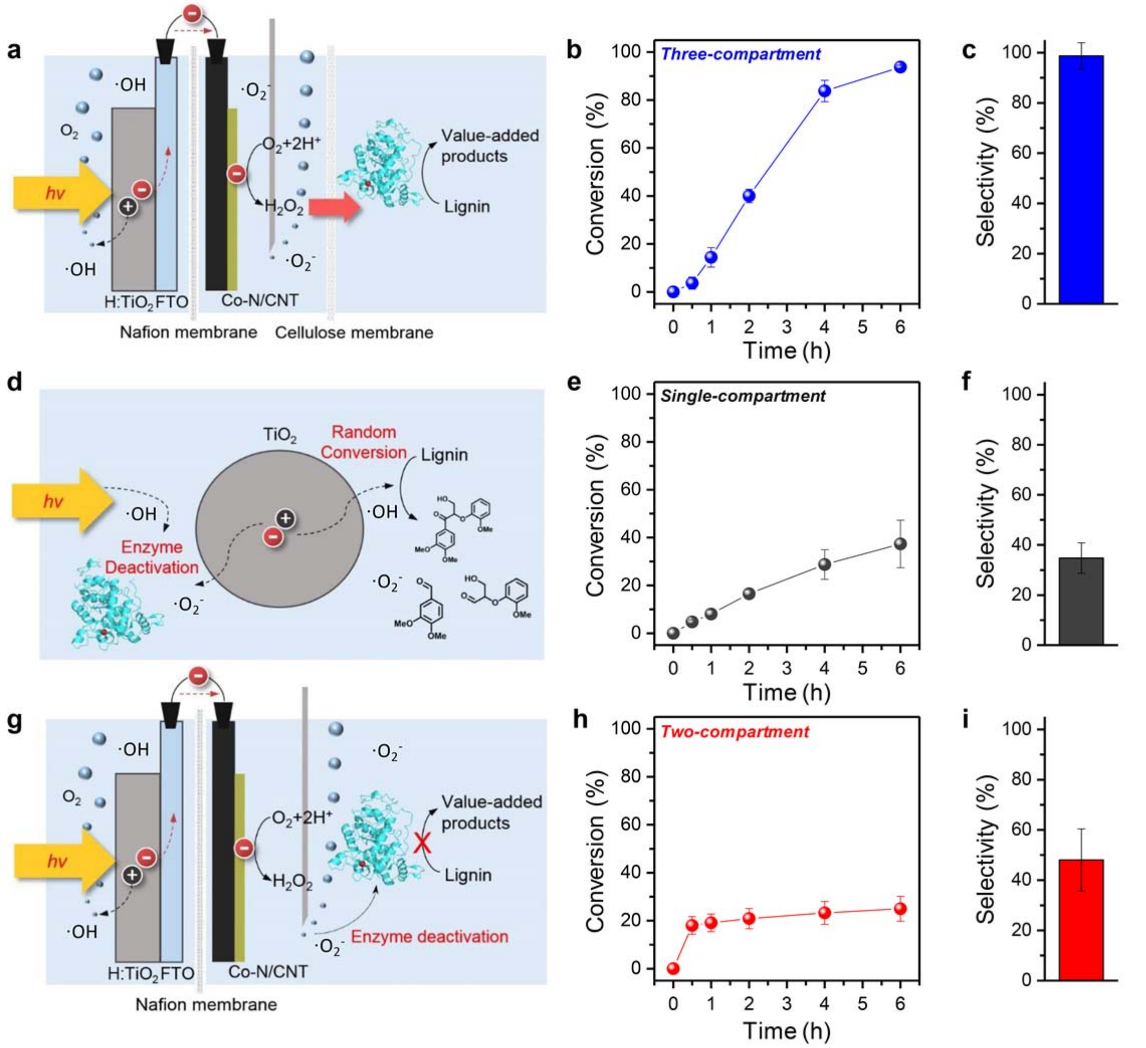

Revised Fig. 3 | Reactor configurations and photo-electro-biochemical lignin dimer conversion. a,**d**,**g**, Schematic representations of the three-compartment photo-electro-biochemical system (**a**), single-compartment powder-based bio-photochemical system (**d**), and two-compartment photo-electrochemical system (**g**) used for lignin dimer conversion. **b**,**e**,**h**, Conversion and **c**,**f**,**i**, selectivity of the three-compartment photo-electro-biochemical system (**b**,**c**), single-compartment powder-based bio-photochemical system (**e**,**f**), and two-compartment photo-electrochemical system (**h**,**i**).

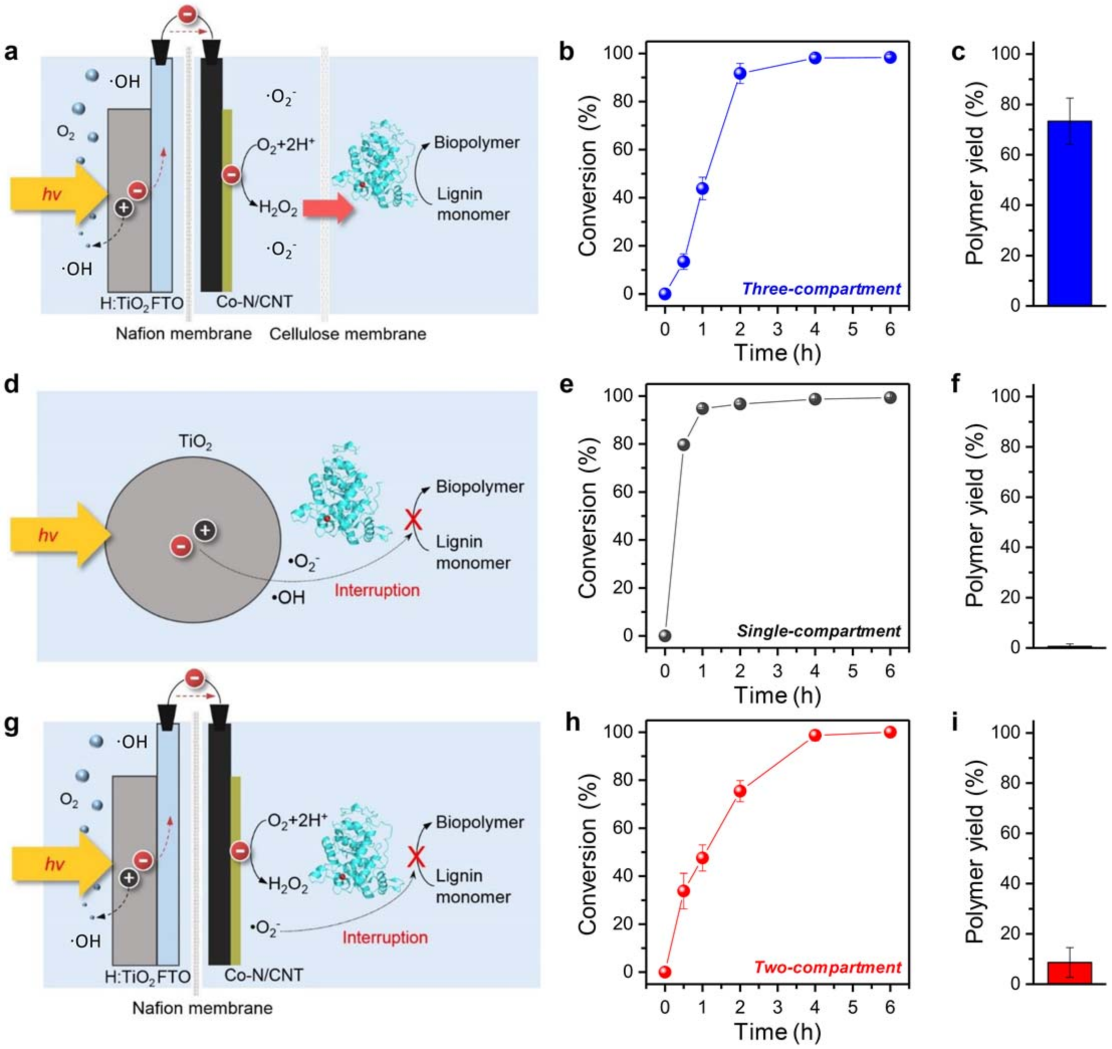

Revised Fig. 4 | Reactor configurations and photo-electro-biochemical synthesis of biopolymer from coniferyl alcohol. **a**,**d**,**g**, Schematic representations of the (**a**), three-compartment photo-electro-biochemical system, (**d**), single-compartment powder-based bio-photochemical system, and (**g**), two-compartment photo-electrochemical system used for biopolymer synthesis. **b**,**e**,**h**, Conversion percentage of coniferyl alcohol over time and **c**,**f**,**i**, polymer yield after 6 h of reaction of the three-compartment photo-electro-biochemical system (**b**,**c**), single-compartment powder-based bio-photochemical system (**e**,**f**), and two-compartment photo-electrochemical system (**h**,**i**).

Accordingly, all other experiments were performed again in the phosphate borate solution (revised Supplementary Fig. 8, 12, 14–17, 23, 24, and 34–37). Please refer to our response to the first question of reviewer #2.

Comment 2: In the manuscript authors claim that in their system lignin is converted efficiently, this cannot be claimed: only very low concentrations of the substrate are used, not lignin but "only" one model component is applied.

Response: We agree with the reviewer's comment and have toned down the expression of "lignin is converted efficiently" in the revised manuscript.

To convert a high concentration of substrate, the photo-electro-biochemical cell should deliver a higher current to generate a larger amount of H_2O_2. A simple way to enhance the amount of H_2O_2 production is by increasing the light intensity or scaling up the photoanode (Supplementary Fig. 11; also see our response to comment 4 below).

Lignin has a heterogeneous structure comprising a variety of linkage types. One of the major challenges in biomass feedstock for the production of organic chemicals is that identification of a green transformation method that can achieve high efficiency and selectivity.

In this study, we started with methylated lignin dimer comprised of the alkyl aryl ether unit linkage (β-O-4), which is one the most common structural units in the lignin structure (vary from 45 up to 84%, *Angew. Chem. Int. Ed.* **55**, 8164–8215 (2016)). In our work, the combination of photo-electrochemical system and biocatalysts in a newly designed three-compartment reactor has proven its capability for a selective transformation of β-O-4 lignin dimer with catalyst of lignin peroxidase isozyme H8 from *Phanerochaete chrysosporium* (LiPH8).

So far, we found that this enzyme selectively catalysed beta-O-4 bond cleavage, however, unfortunately, it does not work well for other bond linkages when the methylated authentic lignin was applied. 2D NMR spectroscopy revealed that the β-O-4 linkages present in the authentic methylated lignin are quantitatively cleaved upon depolymerisation in the presence of the biocatalyst LiPH8. LiPH8/H_2O_2 was found to catalyse the selective cleavage of β-O-4 linkages in lignin both poplar (Review Only Fig. 5A and B) and pine (Review Only Fig. 5C and D). Moreover, this study clearly suggested that there is no effect on other linkages, such as β-β, β-5 (Review only Fig. 5). We suggest that a cocktail of enzymes selectively catalysing different linkages can synergistically/efficiently transform lignin into value-added monomers. To our knowledge, this question has driven much interest to a broad spectrum of researchers having concerns in enzymatically-catalysed biomass utilisation.

We also verified that methylated lignin is successfully depolymerised into 3,4-dimethoxy benzoic acid, 3,4,5-trimethoxy benzoic acid, 3,4,5-trimethoxy benzaldehyde, and 3,4-dimethoxy benzaldehyde in the three compartmented photo-electro-biochemical cell, however further study is needed to identify the percentages β-O-4 linkages in the (methylated) lignin structure to estimate the exact conversion efficiency and the selectivity of lignin.

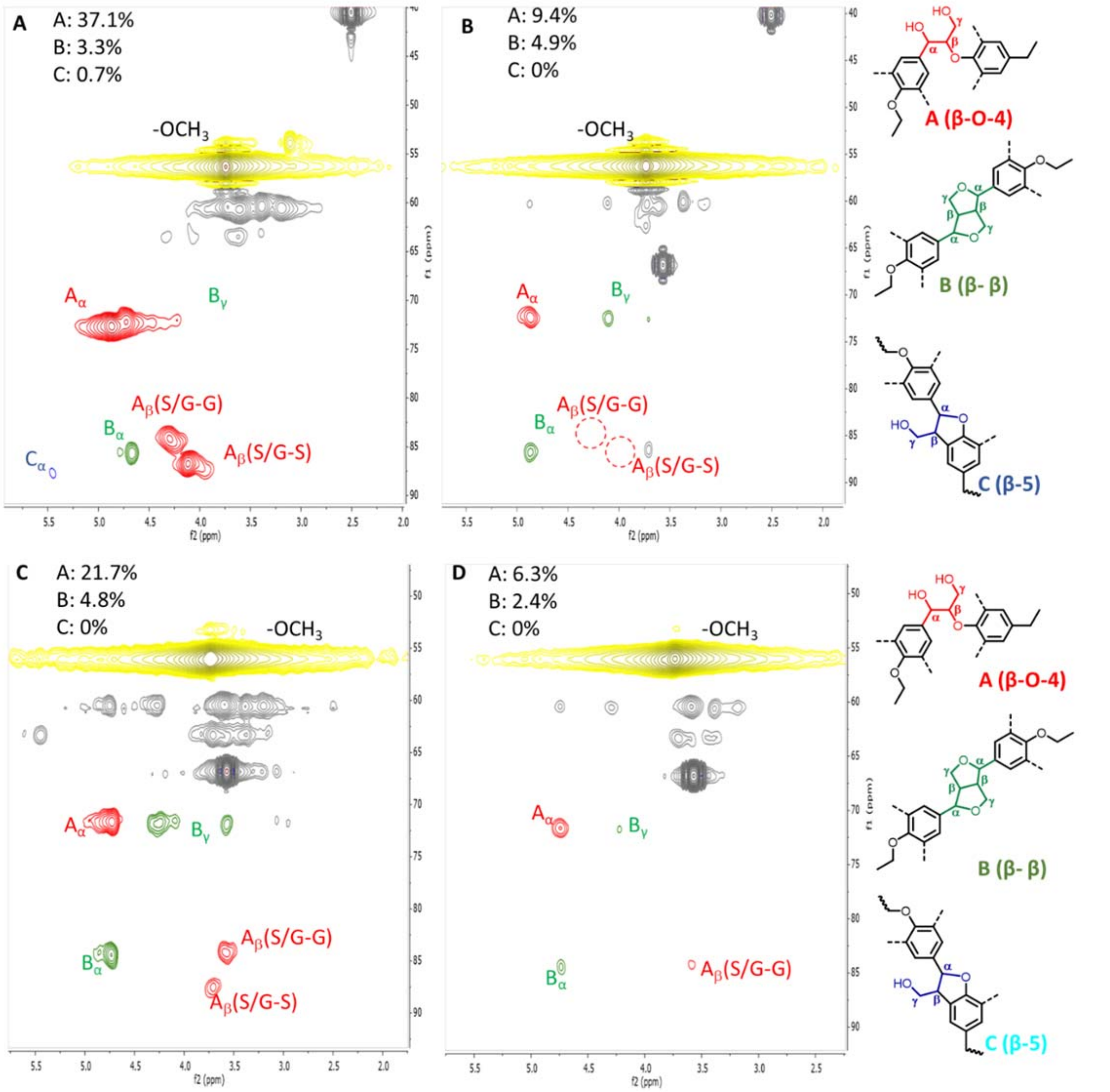

Review Only Fig. 5 2D NMR spectra of methylated lignin aliphatic regions (in DMSO-d$_6$) from poplar (A, B) and pine (C, D) in the absence and presence of the catalyst LiPH8/H_2O_2, respectively. 2D NMR spectra were recorded on 400 MHz instruments (Bruker) with a 5-mm BBO NMR probe. The lignin samples (25 mg) were dissolved in 0.5 ml of deuterated DMS6-d$_6$. Contour coloration matches with structure units shown on the right of the figure. The quantification values are from the relative area integration of aliphatic structures to total lignin aromatic units.

Comment 3: For a comparison with other methods, the entire process chain must be taken into account (here, for example, the synthesis and service life of the photo anode were not taken into account)

Response: Nanostructured titanium dioxide photocatalysts are largely available and are one of the most stable photocatalysts at wide pH ranges among other metal oxides (*Chem. Rev.* **114**, 9919 (2014); *C. R. Chim.* **9**, 750 (2006); *J. Photochem. Photobiol. C* **1**, 1 (2000); *Int. J. Hydrogen Energy* **27**, 991 (2002)). The stability of H:TiO_2 has been evaluated by a chronoamperometry test at a constant applied voltage of 1.23 V (vs. RHE) and is shown in Revised Supplementary Fig. 4b. It shows a stable photocurrent of around 1.2 mA cm^{-2} during 12 h.

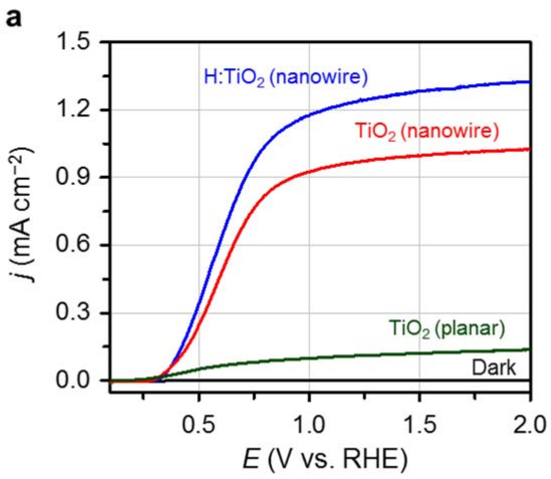

Revised Supplementary Fig. 4 | Photoelectrochemical measurement of photoanodes. a, *I-V* curves hydrogen-treated H:TiO_2/FTO nanowire photoanode (blue line), TiO_2/FTO nanowire photoanode (red line) and TiO_2/FTO planar photoanode (green line). **b**, Stability of the H:TiO_2/FTO photoanode measured at a constant applied voltage of 1.23 V (vs. RHE) in 0.1 M phosphate borate solution, pH 4.5 under simulated 1 sun (AM1.5G) illumination.

Comment 4: The scale-ability of the system must be addressed

Response: The scalability of this system is directly related to the performance of the large scale of PEC system. To show the scalability, we have performed H_2O_2 production with a 5 times larger reactor in volume, 6 times larger illumination area of photoanode, and 5 times larger size of cathode than the original reactor design. The scaled-up system successfully worked by giving an amount of H_2O_2 5.2 times greater than that that of the original reactor for 10 h of reaction, as shown in Supplementary Fig. 11. This result shows that our photo-electro-biochemical three-compartment system is scalable.

For successful scaling-up with the preserved overall efficiency, there are several more

considerations, which are generally insignificant in small-scale systems. These include spatial defects in an enlarged photoanode, increased total Ohmic resistance, energy loss from the membrane and chemical gradients (*e.g.* H^+) (*Chem. Soc. Rev.* **48**, 1908 (2019)). Many fabrication techniques have been actively developed for defect-free large-scale photoanodes *via* ambient-condition solution-based methods (*Chem. Rev.* **115**, 12839 (2015); *Appl. Catal. B* **202**, 388 (2017); *Nanoscale* **8**, 17623 (2016)) and *via* design of multi-electrode (*J. Power Sources* **398**, 224 (2018)). In addition, several strategies have been reported to reduce Ohmic loss by using metallic grid on a substrate (*Energy Environ. Sci.* **10**, 346 (2017); *Int. J. Hydrogen Energy* 36, 5262 (2011)) and to reduce energy loss by design of efficient cell architecture (*Angew. Chem. Int. Ed.* **55**, 12974 (2016); *Energy Environ. Sci.* **6**, 3713 (2013)). Therefore, there is a likely chance to scale up our photo-electro-biochemical lignin conversion system above laboratory level with the proper material, fabrication, and system optimisation.

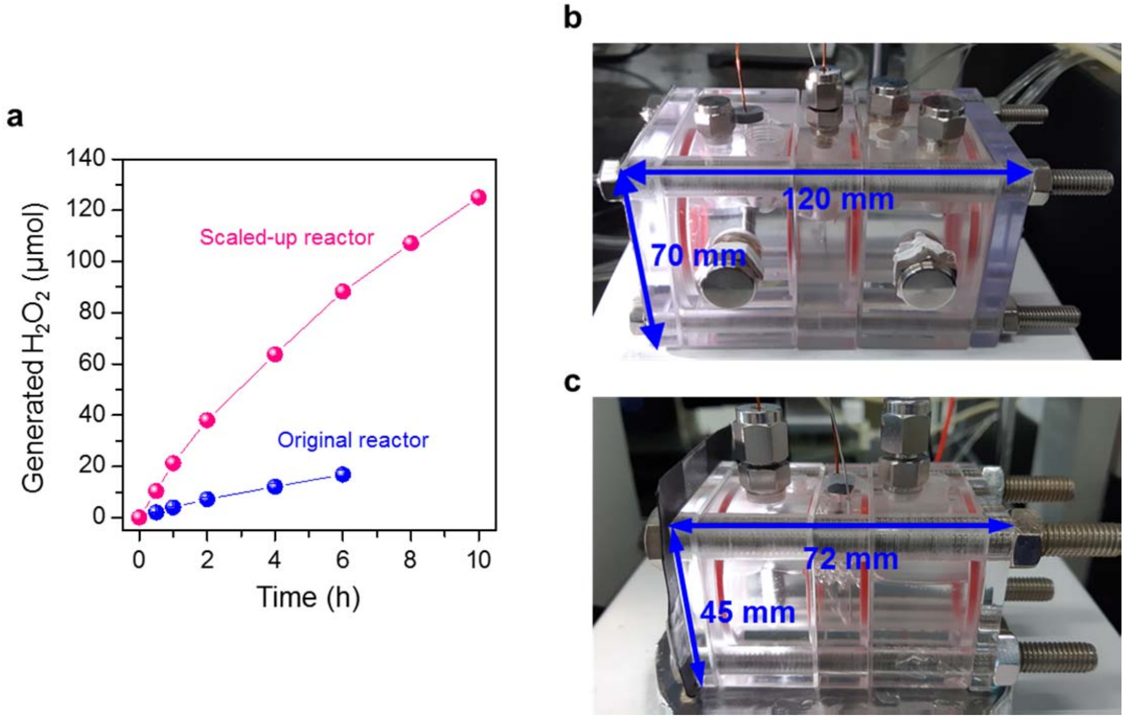

Supplementary Fig. 11 | Photo-electrocatalytic H_2O_2 production in the 5 times scaled-up reactor. a, Amount of H_2O_2 generated over time in 0.1 M phosphate borate solution, pH 4.5 under 1 sun (AM1.5G) illumination in different-sized reactors. **b**,**c**, Digital pictures of the scaled-up and original three-compartment reactors. Electrolyte volume: anode, cathode, and enzyme cells contained 40, 20, and 40 ml of electrolyte for the scaled-up reactor, respectively, while the original reactor contained 5 times lower amounts of electrolyte in each cell. Electrode areas: H:TiO_2 photoanode illuminated area was 8.0 cm^2 and Co–N/CNT cathode area was 10 cm^2 for the scaled-up reactor, while the original reactor had 6 and 5 times smaller photoanode and cathode, respectively. Light source: 300 W Xenon lamp (Newport, 66902) with AM1.5G filter for the scaled-up reactor, while the solar simulator (Abet Technologies, 10500) was used for the original reactor and 100 mW cm^{-2} (AM1.5G) of light intensity was

used for both cases.
We revised relevant contents as follows:

Manuscript text, Page no. 12-13

~~*We observed the stable and continuous H_2O_2 production over 6 h of reaction without signs of deactivation.*~~
→ *"We observed continuous production of H_2O_2 over 6 h of reaction, and found that the H_2O_2 production rate can be easily increased by scaling up the size of electrodes and the reactor (Supplementary Fig. 11)."*

Comment 5: Line 270 – the unspecific conversion seems to be much more efficient.

Response: As a result of new measurement with the acetate-free solution in the single-compartment system, the lignin dimer conversion and selectivity were 37% and 35%, respectively with LiPH8 enzyme. Without enzyme, the lignin dimer conversion and selectivity were 44% and 31%, respectively. As the reviewer commented, we revised and toned down the expression as follows:

Manuscript text, Page no. 16

"The obtained lignin dimer conversion and selectivity were 44.5% and 26.6%, respectively (Fig. 3e,f). Interestingly, a similar conversion and selectivity were obtained without LiPH8 (Supplementary Fig. 15), which indicates that the reaction mainly proceeds via direct photocatalytic lignin conversion by the TiO_2 photocatalyst."
→ *"The obtained lignin dimer conversion and selectivity were ~~44.5%~~ 37.3% and ~~26.6%~~ 34.8%, respectively (Fig. 3e,f). To investigate the role of the enzyme in the single-compartment system, we performed lignin conversion experiments in the absence of LiPH8. Interestingly, a similar range of conversion and selectivity were obtained without LiPH8 (Supplementary Fig. ~~15~~16), which indicates that the reaction mainly proceeds via direct photocatalytic lignin conversion by the TiO_2 photocatalyst."*

REVIEWERS' COMMENTS:

Reviewer #1 (Remarks to the Author):

I recommend the revised paper to publish now.

Reviewer #2 (Remarks to the Author):

The authors have in detail responded to my critizism in the original review and adressed all the issues by performing the requested additional experiments and analyses.

As mentioned in the original review, the novelty and impact are high and since all the issues have now been resolved I recommend the manuscript be published.
